# Genome-wide discovery and characterization of terpene synthases contributing to strawberry aroma metabolism

**DOI:** 10.1093/plphys/kiag292

**Published:** 2026-05-22

**Authors:** Mary A Madera, Farida Yasmin, Kristen L Becker, Jenevieve D Weissman, Randi A Famula, Marta Bjornson, Glenn S Cole, Mitchell J Feldmann, Steven J Knapp, Philipp Zerbe

**Affiliations:** Department of Plant Biology, University of California-Davis, Davis, CA 95616, United States; Department of Plant Sciences, University of California-Davis, Davis, CA 95616, United States; Department of Plant Biology, University of California-Davis, Davis, CA 95616, United States; Department of Plant Sciences, University of California-Davis, Davis, CA 95616, United States; Department of Plant Biology, University of California-Davis, Davis, CA 95616, United States; Department of Plant Sciences, University of California-Davis, Davis, CA 95616, United States; Department of Plant Sciences, University of California-Davis, Davis, CA 95616, United States; Department of Plant Sciences, University of California-Davis, Davis, CA 95616, United States; Department of Plant Sciences, University of California-Davis, Davis, CA 95616, United States; Department of Plant Sciences, University of California-Davis, Davis, CA 95616, United States; Department of Plant Biology, University of California-Davis, Davis, CA 95616, United States

## Abstract

Strawberry (*Fragaria × ananassa*) is a globally cultivated fruit crop appreciated by consumers for its unique flavor and aroma. Major groups of volatile organic compounds that define strawberry aroma include terpenes, fatty acid esters, furanones, and benzenoids, with complex terpene blends contributing characteristic fruity, citrus, and floral notes. Here, we report the genome-wide identification and functional analysis of the terpene synthase (TPS) family that governs terpene chemical diversity in strawberry. Mining of the allo-octoploid genome of the commercial “Royal Royce” cultivar identified 75 TPS gene candidates. Biochemical characterization of 33 mono-, sesqui- and di-TPS enzymes via recombinant protein assays and/or microbial co-expression studies demonstrated TPS activities involved in the biosynthesis of nearly two-thirds of known strawberry terpenes as well as products not previously described in octoploid strawberry. Complementary metabolomic and transcriptomic studies across a diversity panel of strawberry accessions illustrated substantial variation in the composition and abundance of more than 30 common and accession-specific terpene aroma metabolites. Analysis of 2 commercial accessions, “Royal Royce” and “Mara de Bois,” with contrasting aroma profiles further revealed accession-specific alterations of terpene metabolism during fruit ripening. These findings expand our understanding of the biosynthetic genes and pathways underlying strawberry terpene metabolism and provide resources to develop breeding strategies toward improving desirable strawberry aroma traits.

## Teaser

The identification of the strawberry terpene synthase family provides insights into the diversity of terpene-mediated fruit aroma.

## Introduction

Cultivated strawberry (*Fragaria* × *ananassa* Duchesne ex Rozier*, Fa*) originated in Europe in the early 18th century as an allo-octoploid species derived from the hybridization of North American *Fragaria virginiana* (*Fvr*) and South American *Fragaria chiloensis* (*Fc*) (Darrow 1966; Hardigan et al. 2021b). These octoploid progenitors evolved ∼1 million years ago through several polyploidization events involving 4 diploid species, *Fragaria vesca (Fv), Fragaria iinumae*, and 2 currently unidentified diploid progenitors (Njuguna et al. 2013; Tennessen et al. 2014; Edger et al. 2019; Hardigan et al. 2021b; Session and Rokhsar 2023). Over the past 300 yr, strawberry domestication has led to major breakthroughs in enhancing key horticultural traits, including fruit yield, berry size, firmness, and shelf life, and has largely expanded the range of available flavor and other quality traits (Fan et al. 2021b; Porter et al. 2023; Feldmann et al. 2024). These improvements have made strawberry a popular fruit crop among consumers worldwide (Darrow 1966; Ulrich and Olbricht 2016; Yan et al. 2018). Indeed, global strawberry production reached over 14M tons in 2023, with Asia, the Americas, and Europe being the primary producers (FAO 2023).

Closely associated with flavor that is largely driven by sugar and organic acid levels of the fruit (ie, the fleshy receptacle), aroma is a complex multifactorial trait that varies substantially across strawberry cultivars and is directly influenced by genotype, environment, and cultivation practices (Schwieterman et al. 2014; Ulrich and Olbricht 2016; Fan et al. 2021b; Porter et al. 2023). Strawberry aroma is characterized by complex bouquets of over 300 identified volatile organic compounds (VOCs) that include fruity esters, caramellic furans, peachy lactones, green aldehydes, and terpenes (Pyysalo et al. 1979; Bood and Zabetakis 2002; Schwieterman et al. 2014; Fan et al. 2021b). As major components of floral or fruity aroma notes, terpene VOCs have been an attractive target for aroma breeding (Schwieterman et al. 2014; Ulrich and Olbricht 2016; Fan et al. 2021b). Over 25 volatile monoterpenes (C_10_) and sesquiterpenes (C_15_) are known to contribute to strawberry aroma profiles (Zabetakis and Holden 1997; Negri et al. 2015; Urrutia et al. 2017; Duan et al. 2018; Yan et al. 2018). Especially, the fruity, floral, and jasmine-like notes of linalool, terpineol, and nerolidol have been positively associated with sensory quality and flavor intensity (Schwieterman et al. 2014; Ulrich and Olbricht 2016; Fan et al. 2021a). Indeed, strawberry cultivars are typically dominated by linalool, nerolidol, and α-terpineol. By contrast, wild octoploid and diploid strawberry accessions often feature higher abundance of terpenes with more pungent floral, woody, or minty aromas, including α- and β-pinene, limonene, citronellol, myrtenol, myrtenyl acetate, and β-phellandrene (Aharoni et al. 2004; Bianchi et al. 2014; Negri et al. 2015; Ulrich and Olbricht 2016; Urrutia et al. 2017; Duan et al. 2018).

Research over the past 2 decades has provided insights into the biosynthesis of aroma-related VOCs and advanced the breeding of strawberry aroma profiles (Aharoni et al. 2004; Beekwilder et al. 2004; Raab et al. 2006; Chambers et al. 2012; Cumplido-Laso et al. 2012; Zorrilla-Fontanesi et al. 2012; Cruz-Rus et al. 2017; Pillet et al. 2017; Barbey et al. 2021; Fan et al. 2021a, 2022; Oh et al. 2021; Porter et al. 2023). Key enzymes in the generation of terpene chemical diversity are terpene synthases (TPSs) that convert a handful of conserved prenyl diphosphate precursors (ie, geranyl diphosphate [C_10_, GPP], farnesyl diphosphate [C_15_, FPP], and geranylgeranyl diphosphate [C_20_, GGPP]) through complex, electrophilic cyclization and carbon-carbon rearrangement reactions (Chen et al. 2011; Karunanithi and Zerbe 2019). These diverse TPS functions have evolved through repeated events of gene and genome duplication and subsequent sub- and neo-functionalization (Zi et al. 2014; Karunanithi and Zerbe 2019). Therefore, it can be hypothesized that the polyploidization events that led to modern octoploid strawberry have been accompanied by an expansion of the TPS gene family (Njuguna et al. 2013; Tennessen et al. 2014; Edger et al. 2019; Session and Rokhsar 2023). Presently known TPS functions in strawberry include 3 functional nerolidol synthases, namely *F. vesca* NEROLIDOL SYNTHASE 1 (FvNES1), and *F.* × *ananassa* FaNES1 and FaNES2, which feature a dual activity, producing both the sesquiterpene nerolidol and the monoterpene linalool (Aharoni et al. 2004; Fan et al. 2022). However, gene expression of only *FaNES1* was shown to be high enough to quantitatively contribute to strawberry aroma. Although displaying lower transcript abundance, a near-identical *FaNES1t* gene has been correlated with a broader product range, including α-terpineol, myrcene, and (*E*)-β-farnesene (Fan et al. 2022). In addition, *F. vesca* PINENE SYNTHASE (FvPINS) was identified as a monoterpene synthase, producing α-pinene, β-phellandrene, and myrcene found to be abundant in wild, ripe *F. vesca* berries (Aharoni et al. 2004), and a TPS predicted to form germacrene D, FaTPS1, was identified in pathogen-stressed strawberry fruits (Zhang et al. 2022).

To expand our understanding of terpene metabolism in octoploid strawberry, we here describe the identification of the strawberry TPS gene family. Biochemical characterization of 33 TPSs revealed common and accession-specific enzyme products. Combined transcriptomics and metabolomics analyses across a diverse panel of wild and domesticated accessions highlight that differences in TPS functional specificity and gene family composition contribute to variations in terpene aroma profiles in strawberry.

## Results

### Discovery of the TPS gene family in allo-octoploid strawberry

To identify the terpene-metabolic network in octoploid strawberry we mined a haplotype-phased genome assembly of the *F.* × *ananassa* “Royal Royce” cultivar (FaRR1; https://phytozome-next.jgi.doe.gov/info/FxananassaRoyalRoyce_v1_0) (Hardigan et al. 2021a) for candidate genes of the TPS gene family as well as genes of the upstream mevalonate (MVA) and methylerythritol phosphate (MEP) pathways that provide the conserved GPP, FPP, and GGPP precursors (Bergman et al. 2024). First, BLAST searches against curated protein databases (Zerbe et al. 2013; Tiedge et al. 2020) identified a total of 62 candidates with best matches to MEP and MVA pathway genes (Fig. S1, Table S1). Apart from *isopentenyl monophosphate kinase* (*ipk*) and *4-hydroxy-3-methylbut-2-enyl diphosphate synthase* (*hds*) genes—which lacked homoeologous gene copies on chromosome 3C—all identified MEP and MVA pathway gene candidates were represented by 4 syntenic copies in the reference genome (Figs. S2 to S4, Table S2). Notably, 8 gene copies were identified for predicted *acetoacetyl-CoA thiolases* (*aact*), *3-hydroxy-3-methylglutaryl-CoA reductases* (*hmgr*), as well as *FPP synthases* and *GPP* or *GGPP synthases*, which appear in 2 groups of 4 syntenic genes each (Figs. S2 to S4).

**Figure 1:**
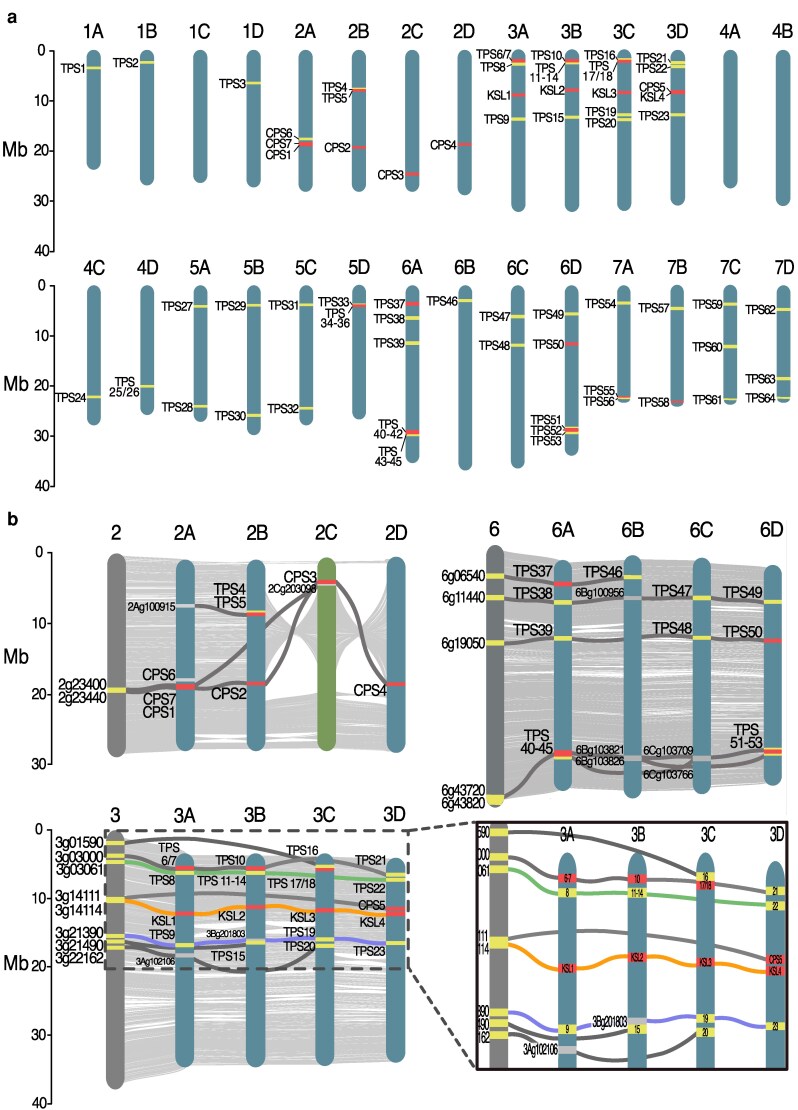
Genomic localization of identified TPS genes in the strawberry genome. a) Relative location of functionally characterized and computationally annotated full-length TPS genes in the *F.* × *ananassa* cultivar “Royal Royce” (FaRR1) genome (Hardigan et al. 2021a, 2021b). CPS, copalyl diphosphate synthase; KSL, kaurene synthase-like. b) Synteny plots of FaRR1 chromosomes 2 (top left), 6 (top right), and 3 (bottom) with TPS genes identified in the diploid *F. vesca* genome accession “Hawaii-4” (gray) (Edger et al. 2018). Gray lines show syntenic relationships between TPS genes. Syntenic pseudogenes are shown in gray. Chromosome 2C is highlighted to depict whole chromosome inversion. Gene-dense region of chromosome 3 shown in detail; lines depict synteny matches across all 5 chromosomes.

Next, BLAST searches of the FaRR1 genome against a dedicated TPS protein database (Zerbe et al. 2013) predicted 104 TPS gene candidates, 75 of which represented full-length transcripts (Fig. 1, Tables S3 and S4). Based on the presence of characteristic DDxxD, DDxD, and RRx_8_W sequence motifs, these 75 TPS candidates were classified as mono- or sesqui-TPSs, Class II diTPSs (copalyl diphosphate synthase, CPS), or Class I diTPSs (kaurene synthase-like, KSL) (Fig. 1). This included the previously characterized NESs, FaNES1, FaNES1t, and FaNES2 (Aharoni et al. 2004; Fan et al. 2022), FvPINS (Aharoni et al. 2004), and FaTPS1 (Zhang et al. 2022). To improve consistency in TPS nomenclature, we here adopted the nomenclature suggested for maize and other species (Köllner et al. 2023) and designated the identified TPS genes as *FaTPS1-64*, *FaCPS1-7*, and *FaKSL1-4*. Accordingly, we propose a renaming of the previously characterized TPSs as *FaTPS17/NES1*, *FaTPS18/NES1t*, *FaTPS11/NES2*, *FvTPS50/PINS*, and *FaTPS26/TPS1*. Notably, *FaTPS17/NES1* and *FaTPS18/NES1t* were identified at the same gene locus (*Fxa3Cg100266.1*), consistent with previous findings (Fan et al. 2022). Six additional TPS genes were present in the FaRR1 genome as sequences substantially longer than known TPS genes and likely represent misassembled or misannotated genes (Fig. S5).

The identified TPS genes were broadly distributed across the FaRR1 genome, with most genes located on chromosomes 3 and 6 (Fig. 1). Although several chromosomes featured closely co-localized TPS genes, no predicted terpene-metabolic gene clusters were identified. Gene synteny analysis of the identified TPSs using the diploid *F. vesca* genome for accession “Hawaii-4” (Edger et al. 2018) and the octoploid FaRR1 genome illustrated that 20 TPSs were present with 4 homoeologous copies across each FaRR1 subgenome and in the *F. vesca* genome, forming 5 syntenic groups with *F. vesca* genes *FvH4_3g23440* on chromosome 2, *FvH4_3g14116* on chromosome 3, *FvH4_5g06470* on chromosome 5, and *FvH4_7g03260* and *FvH4_7g33640* on chromosome 7 (Fig. S6). The remaining TPS genes lacked homoeologous copies on at least one of the FaRR1 subgenomes and were present mostly in syntenic groups of 2 or 3 copies. This included *FvTPS50/PINS*, *FaTPS17/NES1*, *FaTPS18/NES1t* (Aharoni et al. 2004; Fan et al. 2022), and NES-like candidates found in the transcriptomes of *F. chiloensis* and *F. virginiana* (see below) and pinene synthase-like candidates from both *F. virginiana* and *F. vesca* (Figs. S6 to S8). *FaTPS3-5*, *11-15*, *25*, *33*, *41-45*, *53*, and *63* did not feature syntenic equivalents in the diploid *F. vesca* genome, suggesting that they either evolved after polyploidization or originated from one of the other 3 diploid progenitor genomes (Fig. S6; Table S3).

To identify TPSs relevant for aroma metabolism beyond the “Royal Royce” accession, we performed transcriptome analysis of field-grown fruits of “Royal Royce” and 16 additional cultivars, ecotypes, or accessions selected to encompass a range of wild species and diverse breeding materials available in the UC Davis strawberry germplasm collection (Table S5). Transcripts were assembled de novo as well as aligned to the FaRR1 genome and identified 27 full-length transcripts matching TPS genes identified in the FaRR1 genome, including *FaTPS10*, *FaTPS55*, *FaKSL1-4*, *FaCPS5*, *FaTPS17/NES1*, and *FaTPS18/NES1t*. In addition, we identified TPS candidates that featured lower (93% to 95%) sequence identity and may represent distinctive TPSs present in individual cultivars such as *FaTPS7* from the “Primella” cultivar and *FaTPS36* from “Direktor Paul Wallbaum.” Notably, we identified *FaTPS35* and *FaTPS42* in the “Royal Royce” transcriptome, which shared only ∼93% sequence identity with TPSs present in the FaRR1 genome and, therefore, could not be unambiguously matched to any gene identified in the FaRR1 genome. Furthermore, 14 additional TPSs were identified in the transcriptomes of *F. chiloensis*, *F. virginiana*, and *F. vesca* that were apparently distinct from those found in *F.* × *ananassa*. These TPSs were named according to the highest protein sequence similarity to TPSs identified in the FaRR1 genome: *F. chiloensis* ecotype “Ambato” (Amb) *FcKSL5* and *FcTPS18*; *F. chiloensis* ecotype “Isle De Lemuy 02A White” (ILE) *FcTPS40*, *FcTPS52*, and *FcTPS58*; *F. virginiana* accession “NC_96-35-2” (NC) *FvrCPS5*, *FvrTPS17*, and *FvrTPS55*; *F. virginiana* accession “Harris Springs” (HS) *FvrTPS5* and *FvrTPS40*; and *F. vesca* accession “UC06” *FvTPS6*, *FvTPS35*, *FvTPS37*, and *FvTPS50* (Tables S3 and S4). Collectively, analysis of the FaRR1 genome and the obtained transcriptome data revealed 75 putative mono- and sesqui-TPSs and 13 Class II and Class I diTPSs (Table S4).

**Figure 2:**
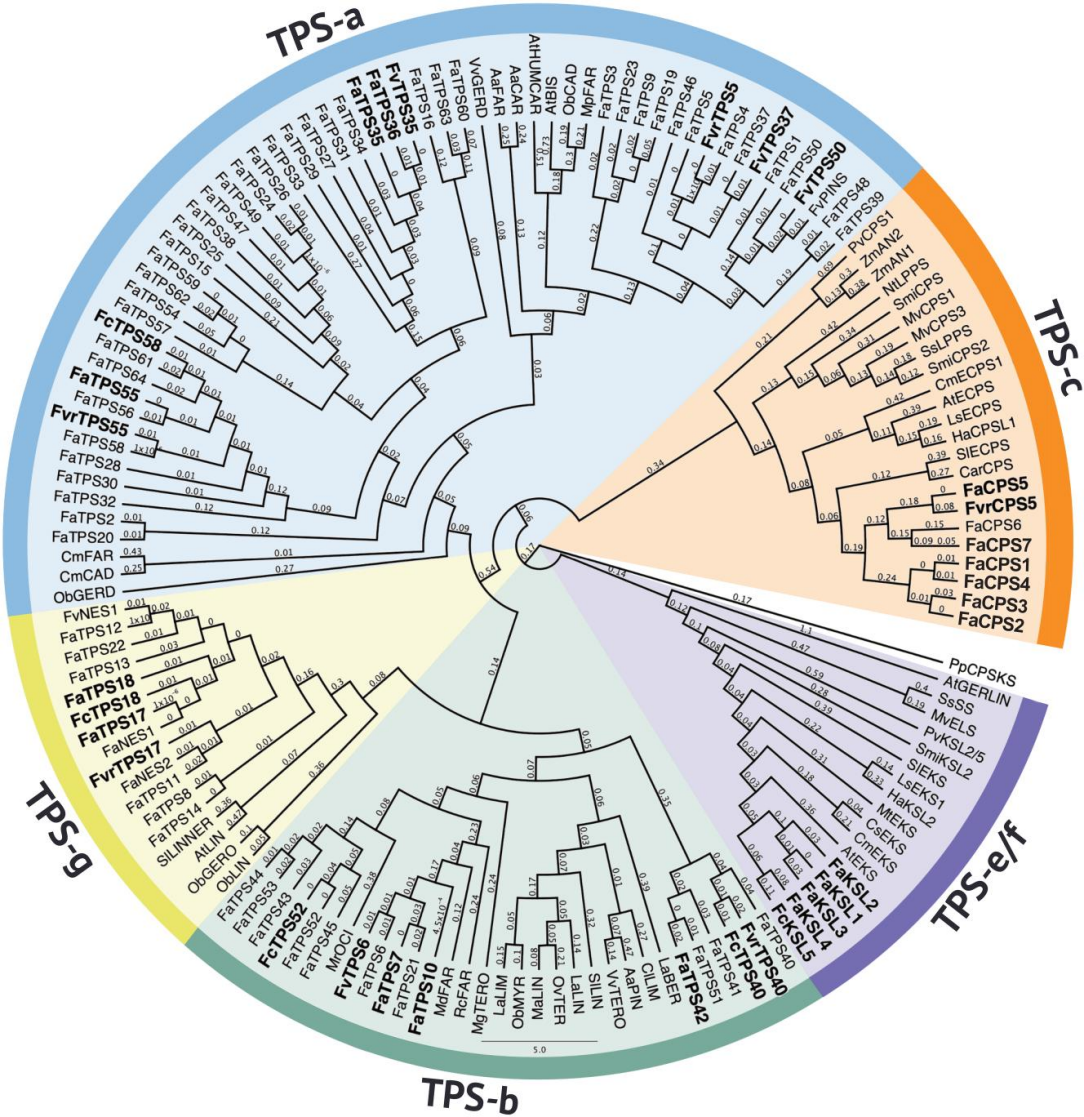
Maximum-likelihood phylogeny of strawberry TPSs and selected TPSs of the TPS-c, TPS-e/f, TPS-b, TPS-g, and TPS-a subfamilies from related plant species. Tree rooted with the *ent*-CPP/*ent*-kaurene synthase from *Physcomitrium patens* (PpCPSKS). Enzyme candidates biochemically characterized in this study are highlighted in bold. Branch support values are average amino acid substitutions per site (500 bootstrap repetitions). Accession numbers and protein sequences are given in Table S4.

Phylogenetic analysis of the identified TPS candidates was performed to substantiate functional annotations. Consistent with the presence of a βα-domain structure, a plastidial transit peptide, and a characteristic RRX_8_W motif (Chen et al. 2011; Karunanithi and Zerbe 2019), 16 TPSs were placed together with known mono-TPSs of the TPS-b subfamily (Fig. 2). Sharing a βα-domain structure but lacking a transit peptide and RRX_8_W motif, 50 TPSs clustered with sesqui-TPSs of the TPS-a subfamily. Ten TPSs showed a close phylogenetic relationship with FaNES1, FaNES2 and related enzymes of the TPS-g subfamily, suggesting similar catalytic functions.

In addition to mono- and sesqui-TPSs with predicted functions in strawberry aroma metabolism, 13 TPS candidates were identified as putative diTPSs. Of these FaCPS1-7 and FvrCPS5 (accession “NC_96-35-2”) were placed within the TPS-c subfamily and featured a DxDD motif characteristic for Class II diTPSs (Zerbe and Bohlmann 2015, Fig. 2, Table S3). Except for *FaCPS5* on chromosome 3D, all CPSs are localized on chromosome 2 and share >96% protein sequence similarity (Fig. S7, Table S6). Presence of a characteristic H-N dyad in the N-terminal catalytic domain of FaCPS1-4 and FaCPS6-7 suggested catalytic functions as diTPSs forming *ent*-CPP (Potter et al. 2014, 2016; Zi et al. 2014, Fig. S9). FaCPS6 featured an N-terminal truncation, lacking part of the γ-domain likely rendering the protein inactive. FaCPS5 and FvrCPS5 shared only 70% sequence identity with the other CPSs and did not feature the H-N dyad or a H501 residue conserved in *syn*-CPP synthases (Potter et al. 2014, 2016; Zi et al. 2014), thus suggesting a distinct function (Figs. S7 and S9). Five KSLs were identified that featured the conserved, catalytic DDxxD and NSE/DTE motifs and formed a separate group within the TPS-e/f subfamily of Class I diTPSs (Figs. 2 and S9). *FaKSL1-3* co-localized on chromosome 3A-C, whereas *FaKSL4* was localized adjacent to *FaCPS5* on chromosome 3D (Figs. 1 and S7). In addition, we identified *FcKSL5* in *F. chiloensis* ecotype “Ambato,” which most closely matches *FaKSL4* in the FaRR1 genome. However, *FaKSL4* represents a probable misassembly rendering the gene over 3 times the expected length of a typical Class I diTPS (Fig. S5), thus preventing further comparative analysis of these diTPSs. Notably, FcKSL5 adopts a βα-domain architecture known only in KSL enzymes involved in specialized diterpenoid metabolism (Zerbe and Bohlmann 2015; Karunanithi and Zerbe 2019, Fig. S9).

### Strawberry mono- and sesqui-TPSs produce known and previously unrecognized aroma metabolites

Based on their predicted functions in terpene aroma metabolism, we selected 33 TPSs for biochemical characterization. Enzyme functional studies were conducted via in vitro enzyme assays using recombinant, purified proteins and commercial GPP and *E*,*E*-FPP substrates and, where further enzyme characterization was needed, in vivo co-expression assays using an *Escherichia coli* platform engineered for terpene production (Cyr et al. 2007; Morrone et al. 2010; Murphy et al. 2019). Enzyme products were analyzed via GC-MS and identified based on comparison to authentic standards or, where standards could not be obtained, annotation based on best matches to mass spectral databases and Kovats retention indices (Figs. S10 to S12; Tables S7 to S9).

**Figure 3:**
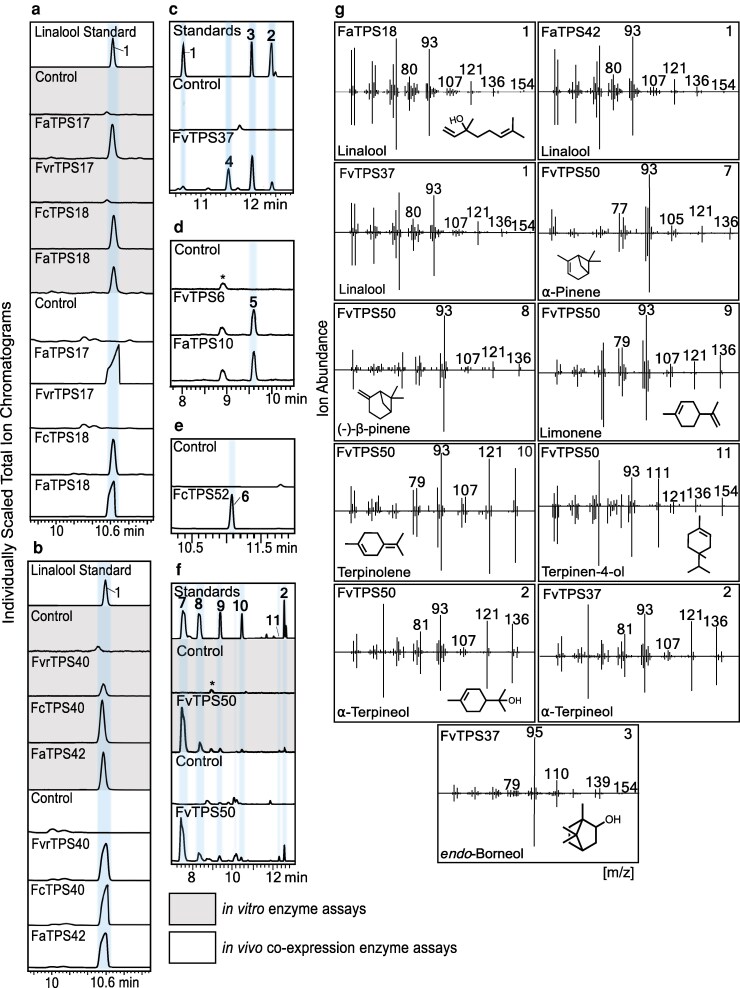
Functional characterization of monoterpene synthases. a to f) GC-MS total ion chromatograms of products resulting from either in vitro enzyme assays of individual recombinant TPSs with GPP as a substrate (gray) or co-expression assays of individual TPSs and an *A. grandis* GPP synthase in *E. coli* (white). g) Mass spectra of enzyme products identified by comparison to authentic standards. FaTPS17, TPS18, and TPS42 taken from *F.*×*ananassa (Fa)* cultivar “Royal Royce”; FvrTPS17 *F. virginiana (Fvr)* accession “NC_96-35-2”; FcTPS18 *F. chiloensis (Fc)* ecotype “Ambato”; FvrTPS40 accession “Harris Springs”; FcTPS40 and FcTPS52 ecotype “Isle de Lemuy”; FvTPS6, FvTPS37 and FvTPS50 *F. vesca (Fv)* diploid accession “UC06”; FaTPS10 cultivar “EarliMiss.” Identified compounds: 1, Linalool; 2, α-terpineol; 3, endo-borneol; 7, α-pinene; 8, (-)-β-pinene; 9, limonene; 10, terpinolene; 11, terpinen-4-ol.

GC-MS analysis of TPS enzyme products revealed catalytic activities for all tested TPSs except for FvrTPS5 (accession “Harris Springs”) and FvrTPS17 (accession “NC_96-35-2”). In vitro and in vivo enzyme assays with GPP as a substrate confirmed the formation of linalool **1** by FaTPS17/NES1 and FaTPS18/NES1t as previously reported (Aharoni et al. 2004; Fan et al. 2021a). Furthermore, we identified FaTPS42 (“Royal Royce”), FcTPS18 (ecotype “Ambato”), FvrTPS40 (“Harris Springs”), and FcTPS40 (ecotype “Isle de Lemuy”) as additional linalool synthases present in strawberry (Fig. 3). In vitro assays of FvTPS37 and FvTPS6 (accession “UC06”), FaTPS10 (cultivar “EarliMiss”), and FcTPS52 (“Isle de Lemuy”) did not show detectable enzyme activity. However, *E. coli* co-expression studies of these TPSs with an *Abies grandis* GPP synthase identified FvTPS37 as a multi-product enzyme, forming α-terpineol **2**, borneol **3**, and *trans*-thujanol **4** as major products with small amounts of linalool **1** as based on comparison to mass spectral databases and Kovats indices (Figs. 3 and S10). In addition, FaTPS10 and FvTPS6 produced β-(*E*)-ocimene **5** and FcTPS52 formed β-(*Z*)-ocimene **6** based on closest database matches (Figs. 3 and S10). FvTPS50 (“UC06”) was identified in vitro and in vivo as a multi-product mono-TPS, forming α-pinene **7** as a major product as well as small quantities of β-pinene **8**, limonene **9**, terpinolene **10**, terpinen-4-ol **11**, α-terpineol **2**, and *trans*-thujanol **4** (Figs. 3 and S10).

**Figure 4:**
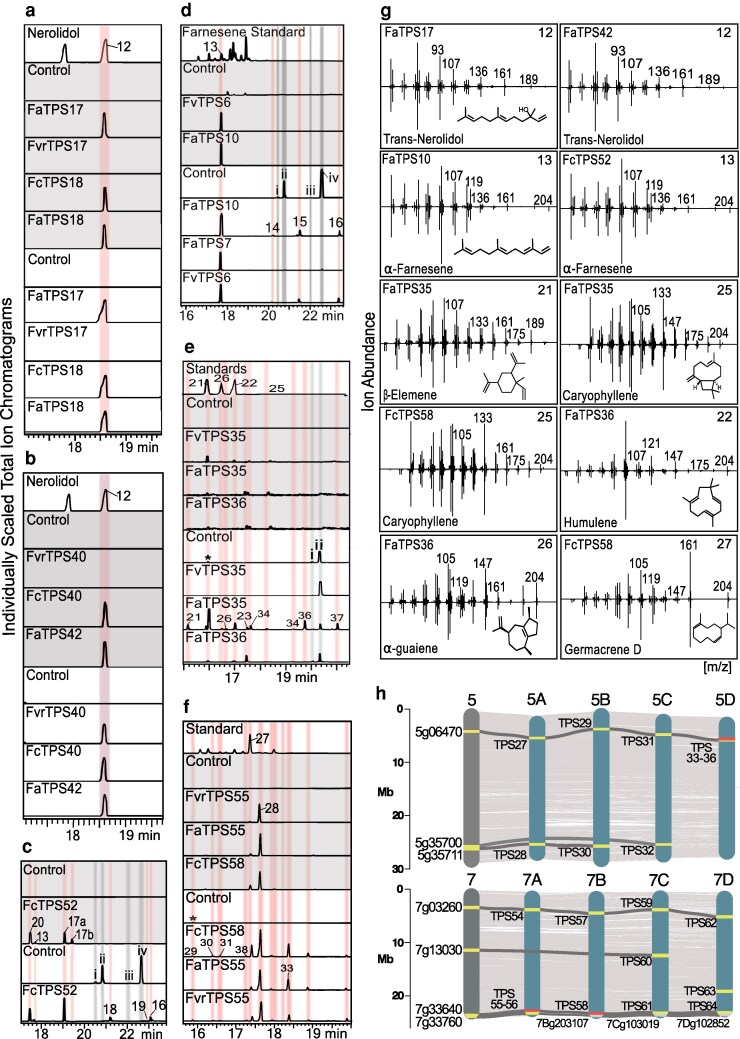
Functional characterization of sesquiterpene synthases. a to f) GC-MS total ion chromatograms of products resulting from either in vitro enzyme assays of individual recombinant TPSs with farnesyl diphosphate (*E*,*E*-FPP) as a substrate (gray) or co-expression assays of individual TPSs and a FPP synthase in *E. coli* (white). g) Mass spectra of enzyme products identified by comparison to authentic standards. h) Synteny plots of FaRR1 chromosomes 5 and 7 with *F. vesca.* The following compounds are likely degradation products of FPP produced in *E. coli* cultures: (i) *cis-trans*-farnesene; (ii) farnesol; (iii) 2,3-dihydro farnesyl acetate; (iv) *trans*-farnesyl acetate. FaTPS17, TPS18, TPS35, and TPS42 from *F.* × *ananassa (Fa)* “Royal Royce”; FvrTPS17 and FvrTPS55 *F. virginiana* (*Fvr*) “NC_96-35-2”; FcTPS18 and FcTPS58 *F. chiloensis* (*Fc*) “Ambato”; FvrTPS40 “Harris Springs”; FcTPS40 and FcTPS52 “Isle de Lemuy”; FvTPS6, FvTPS35, and FvTPS50 *F. vesca* (*Fv*) “UC06”; FaTPS7 “Primella”; FaTPS10 “EarliMiss”; FaTPS55 “Madame Moutot.” Identified compounds: 12, *trans*-nerolidol; 13, α-farnesene; 14, bulnesol; 15, β-acoradienol; 16, 8,13-cedranediol; 21, β-elemene; 22, humulene; 23, β-bisabolene; 24, β-acoradiene; 25, caryophyllene; 26, α-guaiene; 34, cyperene; 35, longifolol; 36, eudesm-7(11)-en-4-ol acetate; 37, hinesol acetate.

Additional assays with *E*,*E*-FPP as the substrate showed that the identified linalool synthases, FaTPS17/NES1, FvrTPS17, FcTPS18, FaTPS18/NES1t, FvrTPS40, FcTPS40, and FaTPS42, also produced *trans*-nerolidol **12** (Fig. 4). This confirms the previously reported activities of FaTPS17/NES1 and FaTPS18/NES1t (Aharoni et al. 2004; Fan et al. 2022) and demonstrates that the strawberry genome contains additional dual function linalool/nerolidol synthases. In vitro enzyme assays identified FaTPS10, FaTPS7 (cultivar “Primella”) and FvTPS6 as α-farnesene **13** synthases (Fig. 4). Additional in vivo *E. coli* co-expression assays of FaTPS10 and FvTPS6 with a maize FPP synthase further detected small quantities of bulnesol **14**, β-acoradienol **15**, and 8,13-cedranediol **16** annotated based on database references and Kovats indices. These products may represent additional TPS products or oxygenated derivatives thereof formed by endogenous *E. coli* enzymes, considering that these compounds were observed in in vivo co-expression studies but not in vitro enzyme assays (Figs. 4 and S11). Furthermore, FcTPS52 produced (*Z*,*E*)-α-farnesene **20** and bisabolene **17** as major products, as well as small amounts of khusimol **18** and several other sesquiterpenes such as α-chenopodiol **19**, 8,13-cedranediol **16**, and α-farnesene **13** (Figs. 4 and S11). Protein yields generated for recombinant FvTPS35 (“UC06”), FaTPS35 (“Royal Royce”), and FaTPS36 (“Direktor Paul Wallbaum”) were consistently low. However, in vitro assays with *E*,*E*-FPP as a substrate resulted in the formation of β-elemene **21** and humulene **22** in addition to trace amounts of additional sesquiterpenes, likely including β-bisabolene **23**, β-acoradiene **24**, and caryophyllene **25** as based on best database and Kovats index matches (Figs. 4 and S12).

Additional co-expression assays confirmed the production of β-elemene **21** and humulene **22** by FaTPS35 and FaTPS36, respectively, alongside a range of minor sesquiterpene products such as α-guaiene **26**, whereas co-expression of FaTPS36 showed β-bisabolene **23** as the primary product. In vitro assays of FvrTPS55 (“NC_96-35-2”), FaTPS55 (cultivar “Madame Moutot”), and FcTPS58 (“Ambato”) identified germacrene D **27** and α-muurolene **28** as major products as based on an authentic standard and mass spectral database matches, respectively (Figs. 4 and S12). Complementary co-expression assays confirmed these enzyme activities and showed additional lower abundant byproducts, including viridiflorene **29**, germacrene A **30**, macrocarpene **31**, eremoligenol **32,** globulol **33**, and several other byproducts (compounds **34-41)** as based on Kovats indices and mass spectral databases (Fig. S12). Additional products that were present in trace amounts following TPS co-expression assays and control samples possibly represent isomerization or metabolism products of FPP, such as *cis*-*trans*-farnesene **i**, dauca-4(11),7-diene **ii**, 2,3-dihydro farnesyl acetate **iii**, and *trans-*farnesyl acetate **iv** (Fig. S11**)**.

### Pairwise activity of strawberry diterpene synthases produces specialized diterpenoids

**Figure 5:**
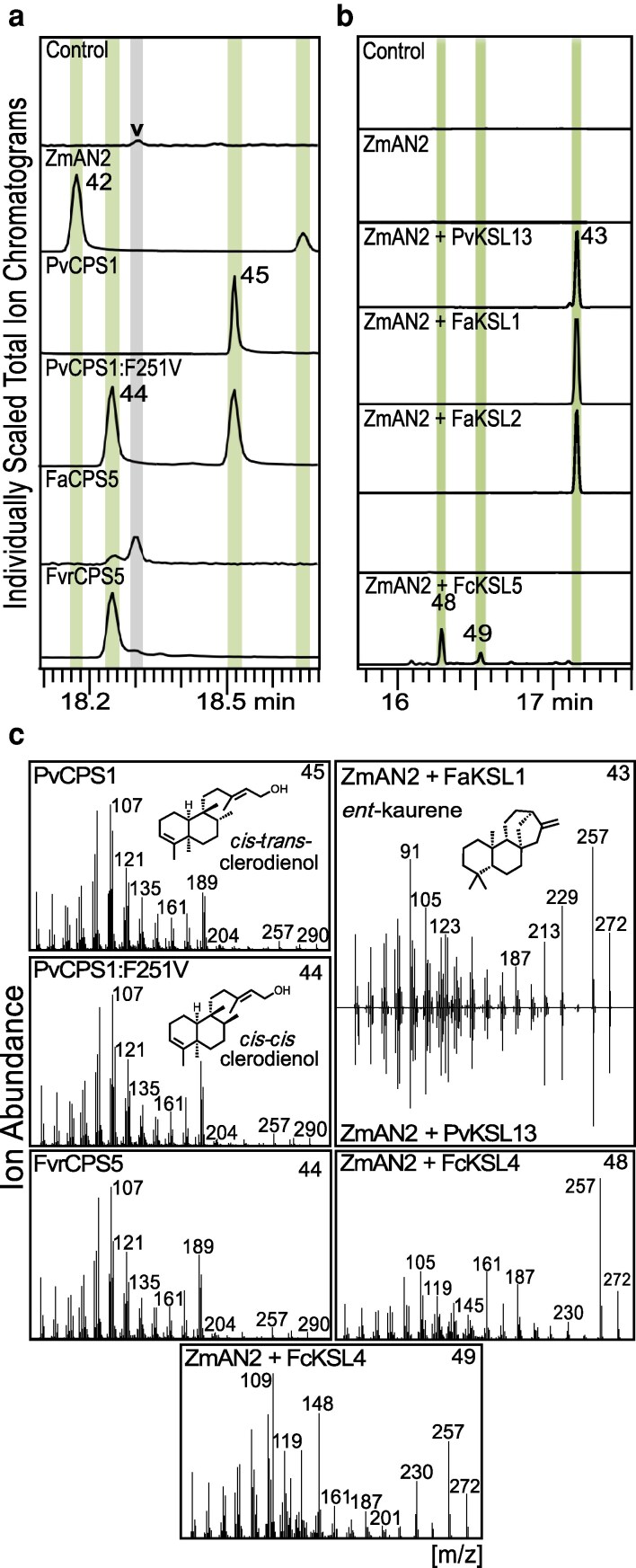
Functional characterization of diterpene synthases (diTPSs). a) GC-MS total ion chromatograms of products resulting from co-expression assays of FaCPS5 or FvrCPS5 when compared with authentic standards produced by the *ent*-CPP synthase *Z. mays* AN2 (ZmTPS38/CPS2/AN2; Harris et al. 2005), the *cis-trans*-CLPP synthase *P. virgatum* CPS1 (PvCPS1; Pelot et al. 2018) and the PvCPS1 variant *PvCPS1:F251V* producing *cis-cis*-CLPP and *cis-trans*-CLPP. b) GC-MS traces of products resulting from co-expression assays of FaKSL1-4 and FcKSL5 with ZmTPS38/CPS2/AN2. c) Mass spectra of enzyme products identified by comparison to enzyme-produced standards. FaCPS5, and FaKSL1-4 taken from *F.* × *ananassa (Fa)* “Royal Royce”; FvrCPS5 *F. virginiana* (*Fvr*) “NC_96-35-2”; FcKSL5 *F. chiloensis* (*Fc*) “Ambato.” Identified compounds: 42, *ent*-CPP; 43, *ent*-kaurene; 44, *cis-cis*-clerodienyl diphosphate (CLPP); 45, *cis-trans*-CLPP; 46/47, 2 pimaradiene-type diterpenoid products; 48, clerodienol; 49, kolavenool.

Although not expected to contribute to strawberry aroma, we biochemically characterized selected diTPS candidates to assess the biosynthetic network underlying diterpenoid metabolism in strawberry. Of the identified Class II diTPSs, *FaCPS1-4* and *FaCPS7* featured a characteristic H-N dyad (Fig. S9), suggesting *ent*-CPP synthase functions. Biochemical characterization of these diTPSs via *E. coli* co-expression of FaCPS1-4 with an *A. grandis* GGPP synthase verified the production of *ent*-CPP **42**, albeit at very low product abundance. However, additional co-expression with a known *ent*-kaurene synthase from switchgrass (*Panicum virgatum*), PvKSL13 (Pelot et al. 2018) resulted in the production of *ent*-kaurene **43 (**a direct derivative of diTPS-catalyzed *ent*-CPP), thus verifying the *ent*-CPP synthase activity of FaCPS1-4 (Fig. S13). By contrast, FaCPS7 showed no detectable enzyme activity. Biochemical analysis of additional Class II diTPS candidate, FvrCPS5, resulted in the formation of *cis-cis*-clerodienyl diphosphate (CLPP) **44** as identified by comparison to the previously reported products of the switchgrass PvCPS1 forming *cis-trans*-CLPP **45**, and a PvCPS1:F251V variant that forms *cis-cis*-CLPP **44** (Pelot et al. 2018, 2019) (Figs. 5 and S14). Next, co-expression assays of FaKSL1-4 or FcKSL5 with an *ent*-CPP synthase from *Zea mays* (ZmTPS38/CPS2/AN2) and the *A. grandis* GGPP synthase were performed. No product formation was detected when co-expressing FaKSL3 or FaKSL4 (Figs. 5 and S13). By contrast, co-expression of ZmTPS38/CPS2/AN2 with FaKSL1 or FaKSL2 yielded *ent*-kaurene **43** as verified by an authentic standard. In addition, pairwise activity of ZmTPS38/CPS2/AN2 with FcKSL5 resulted in 2 pimaradiene-type diterpenoid products **46/47** based on mass spectral comparison to known pimaradiene scaffolds (Figs. 5 and S14). Further co-expression of FcKSL5 or FaKSL1 with PvCPS1 or PvCPS1:F251V yielded clerodienol **48** and kolavenool **49** derivatives. However, low abundance of the above KSL products prevented further purification and structural verification by nuclear magnetic resonance analysis.

### Terpene metabolite profiles underwent substantial changes throughout strawberry domestication

**Figure 6:**
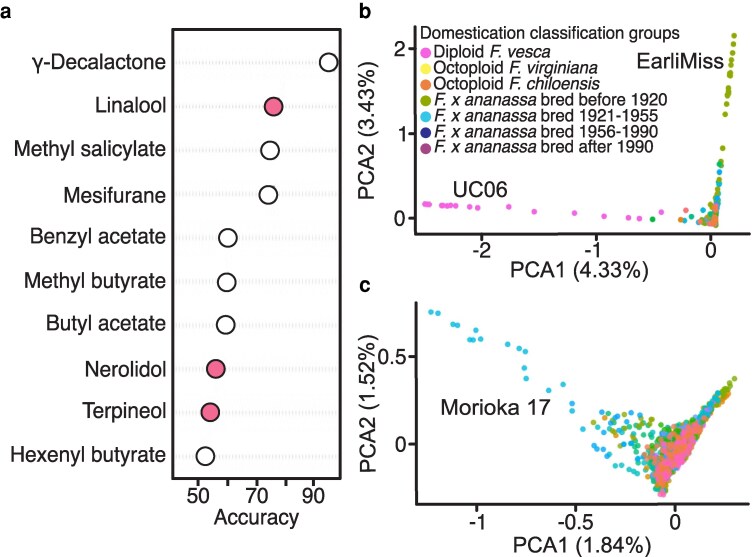
VOC analysis of ripe berries harvested from 96 different, field-grown accessions. a) Scree plot of mean decrease accuracy values from random forest analysis depicting the top distinguishing VOCs among the 96 accessions used for quantitative metabolite profiling via SPME-GC-MS analysis. Terpene VOCs are highlighted. b) PCA plot of VOC profiles from samples harvested in 2021 and 2022. c) PCA plot of VOC profiles from samples harvested in 2021 and 2022, excluding samples from the cultivar “EarliMiss” and diploid accession “UC06.” Information on the 96 field-grown accessions used for this study are given in Table S7.

To assess the diversity of terpene aroma compounds in strawberry we selected a panel of 96 strawberry accessions, cultivars, and wild ecotypes that have been curated and developed by the UC Davis Strawberry Breeding Program and represent a diverse range of aroma profiles across the germplasm (Table S10). Ripe fruits were harvested from field-grown plants during the 2021 and 2022 seasons and analyzed via SPME-GC-MS. A total of 171 VOCs were detected, which included 31 terpene metabolites, as well as a broad variety of short- and medium chain acetic, butyl, and hexyl acid esters such as methyl and hexyl butyrate, alcohols and aldehydes such as 2-hexanol and 2-hexenal, mesifurane, and γ-decalactone (Fig. S15, Data S1, Table S7). Notably, a random forest analysis of the 171 identified metabolites demonstrated that linalool **1**, nerolidol **12**, and α-terpineol **2** ranked among the 10 most relevant metabolites defining the metabolic differences across the analyzed strawberry accessions, alongside other key strawberry aroma compounds such as γ-decalactone and mesifurane (Fig. 6). Principal components analysis (PCA) based upon the volatile random forest data showed that most samples cluster around 0 with *F. vesca* “UC06” samples spreading out across PCA1, and cultivar “EarliMiss” stretching across PCA2, which explain 4.3% and 3.4% of the variance, respectively (Fig. 6).

When excluding “EarliMiss” and “UC06” from the analysis, samples of 1 additional cultivar, “Morioka 17,” were identified as distinctive (Fig. 6). “UC06” featured the highest abundance of terpenes among all tested samples with regard to terpene composition, consistent with its metabolic distinctiveness (Data S1). For instance, “UC06” was the only accession consistently containing 1-*R*-myrtenal **50** and both α- **7** and β-pinene **8** and was 1 of 5 accessions for which different combinations of β-myrcene **51**, β-phellandrene **52**, β-selinene **53**, humulene **22**, *trans*-pinocarvyl acetate **54**, and (−)-myrtenol **55** were detected. “UC06” further contained ≥165 times higher amounts of myrtenyl acetate **61** when compared with all other tested accessions (Data S1). In addition, cultivars “EarliMiss” and “Morioka 17” differed from other accessions in the presence of *E*-β-farnesene **56** and D-limonene **9** and the lack of several fatty acid esters commonly detected in strawberry.

**Figure 7:**
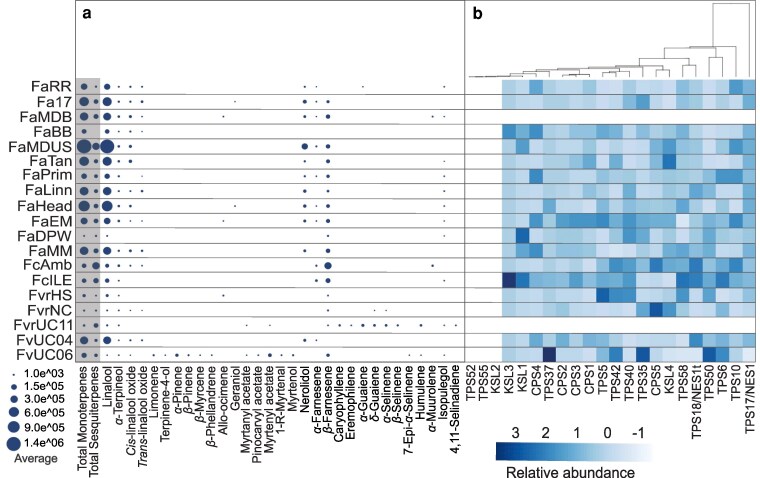
Abundance of terpene metabolites and relevant TPS genes in field-grown strawberry fruits. a) Dot plot representing average normalized peak area of terpenes identified via SPME-GC-MS analysis of metabolites extracted from ripe fruit of field-grown strawberry plants. b) Hierarchical cluster analysis performed on gene expression data of select *TPS* genes analyzed in this study. *TPSs* were identified based on mapping functionally characterized TPSs against the FaRR1 genome (Hardigan et al. 2021a). Scaled gene expression data are based on 3 biological replicates. *F.* × *ananassa (Fa)* cultivars “Royal Royce” (RR), “17C224P011” (17), “Mara des Bois” (MDB) for which no field-grown transcriptome data was available, “Beaver Belle” (BB), “MDUS 5130” (MDUS), “Tangi” (Tan), “Primella” (Prim), “Linn”, “Headliner” (Head), “EarliMiss” (EM), “Direktor Paul Wallbaum” (DPW), and “Madame Moutot” (MM). *F. chiloensis* (*Fc*) “Ambato” (Amb) and “Isle de Lemuy 02A White” (ILE). *F. virginiana* (*Fvr*) “Harris Springs” (HS), “NC_96-35-2” (NC), “UC11”. Diploid *F. vesca* (*Fv*) “UC04” and “UC06'”

Linalool **1** represented the most abundant terpene in most tested accessions, especially in domesticated *F.* × *ananassa* cultivars. However, across the 2021 and 2022 sample collections, the average abundance of linalool **1** and nerolidol **12** showed high variation both within samples of individual accessions and across accessions (Fig. 7, Data S1). In particular, modern cultivars “17C224P011,” “MDUS 5130,” and “Headliner” showed the highest abundance of linalool **1** and nerolidol **12**. By contrast, several wild or older domesticated accessions, including *F. chiloensis* “Isle de Lemuy,” *F.* × *ananassa* “Direktor Paul Wallbaum,” *F. virginiana* accessions “Harris Springs” and “NC_96-35-2,” and *F. vesca* “UC06” featured overall low terpene diversity and abundance, including no or low abundance of linalool **1** and nerolidol **12**. Additional abundant terpenes in the tested accessions were annotated based on best database matches and Kovats indices along with mass spectra, including α-terpineol **2**, *cis*- **57** and *trans*-linalool oxide **58**, nerolidol **12**, and α- **13** and (*E*)-β-farnesene **56**. By contrast, several terpene metabolites were only detected in individual accessions, including allo-ocimene **59** (“EarliMiss,” “Mara des Bois” and *F. virginiana* “Harris Springs”), geraniol **60** (“Headliner” and “17C244P011”), and α-muurolene **28** (*F. chiloensis* “Ambato” and “Mara des Bois”). Two wild accessions showed a particularly high diversity of terpene metabolites. The diploid *F. vesca* “UC06” displayed a diverse monoterpene profile, featuring α-pinene **7**, myrtenyl acetate **61**, limonene **9**, terpinen-4-ol **11**, β-pinene **8**, β-myrcene **51**, β-phellandrene **52**, *trans*-pinocarvyl acetate **54**, 1-*R*-myrtenal **50**, (-)-myrtenol **55**, (*E*)-β-farnesene **56**, 4,11-selinadiene **62**, humulene **22**, and isopulegol **63** as based on authentic standards or best matches to reference mass spectra (Figs. 7 and S15). By contrast, the wild accession *F. virginiana* “UC11” was low in monoterpenes but contained a range of sesquiterpenes not abundant in other accessions, including caryophyllene **25**, eremophilene **64**, α- **26** and δ-guaiene **65**, α- **66** and β-selinene **53**, 4,11-selinadiene **62**, and humulene **22**. Additionally, small differences in the VOC profiles were detected when fruits from the same plants were processed fresh or after flash freezing (Figs. 7 and S16). For example, allo-ocimene **59** was detected in higher amounts in flash frozen *F. virginiana* “Harris Springs” samples and 2 other accessions harvested for transcriptomic and VOC analysis, yet it was not detected in the corresponding fruits harvested and processed fresh. Although freshly processed samples contained more terpenes, several terpenes, γ-dihydro-terpineol, carvomenthol, and γ-bisabolene, were detected only in flash frozen tissues.

To complement metabolite profiles, we used the generated transcriptome data of field-grown, ripe fruits of 2 diploid *F. vesca*, 2 *F. chiloensis*, 2 *F. virginiana* accessions, and 12 *F.* × *ananassa* accessions (ranging from 1910 and 2017) (Table S5). Overall, the gene expression levels of identified TPSs did not show substantial correlation with the terpene metabolite profiles (Fig. 7). However, select TPSs displayed transcript abundance in alignment with accession-specific terpene profiles. For example, high expression of the pinene synthase *FaTPS50/PINS*, terpinolene synthase *FaTPS37*, ocimene synthase *FaTPS6*, and NES *FaTPS42* in diploid accession “UC06” was aligned with the predominant presence of monoterpenes.

Genotypes containing higher amounts of α-/β-farnesene **13**, **45** also showed higher abundance of the farnesene synthases *FaTPS6* and *FaTPS10*, including cultivars “MDUS 5130,” “Primella,” and *“*EarliMiss,” and *F. chiloensis* “Ambato” and “Isle de Lemuy.” However, *FaTPS6* or *FaTPS10* were also abundant in cultivars producing no or low levels of farnesene such as “Royal Royce” and “Madame Moutot.” Similarly, with the exception of *F. vesca* “UC06” that showed relatively high abundance of *FaTPS18/NESt*, genotypes abundant in linalool **1** and nerolidol **12** did not show high gene expression of NESs *FaTPS17/NES1*, *FaTPS18/NESt*, *FaTPS40*, and *FaTPS42*.

### Alteration of terpene metabolism during fruit ripening

**Figure 8:**
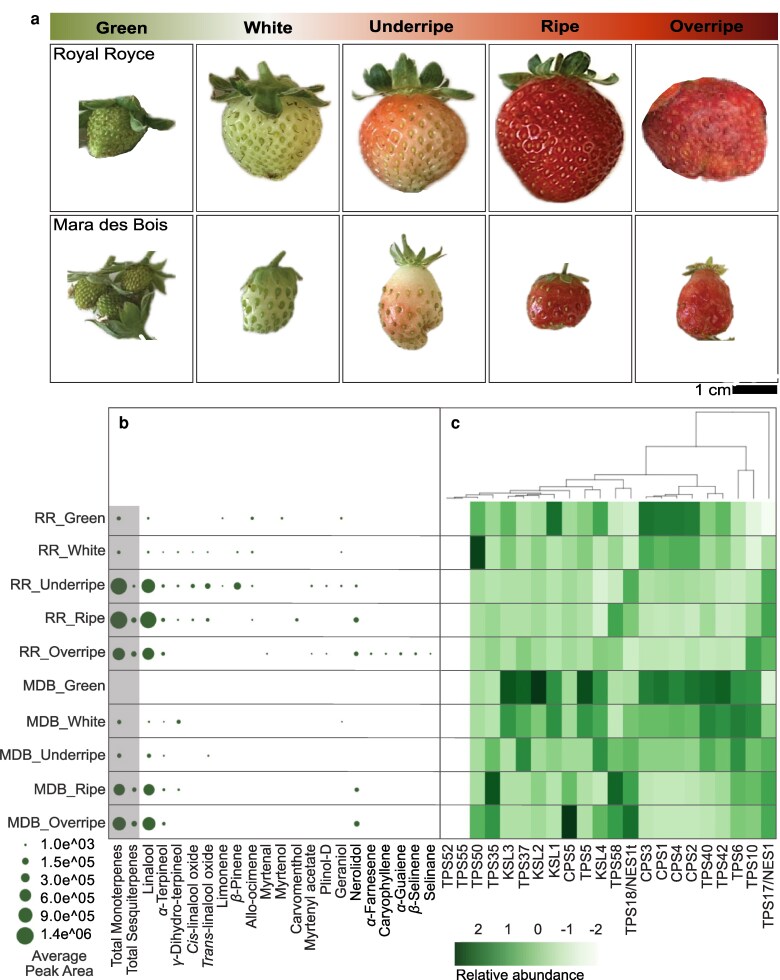
Alterations in terpene metabolism during fruit ripening. a) Images of representative *F.* × *ananassa* “Royal Royce” (RR) and “Mara De Bois” (MDB) berries at the green, white, underripe, ripe, and overripe developmental stages. b) Dot plot showing average normalized peak area of terpenes identified via SPME-GC-MS analysis of metabolites extracted from berry tissue. Note that no MDB tissue was available for metabolite profiling of green fruit due to limited berry production in this cultivar. c) Hierarchical cluster analysis performed on gene expression data of select TPS genes analyzed in this study using the same berry tissues. *TPSs* were identified based on mapping functionally characterized TPSs against the FaRR1 genome (Hardigan et al. 2021a). Scaled gene expression data are based on 3 biological replicates.

To assess changes in terpene abundance during fruit ripening, 2 modern *F.* × *ananassa* cultivars, “Royal Royce” and “Mara des Bois,” which feature contrasting fruit properties and are frequently used for breeding efforts centered around aroma, fruit quality, and yield, were grown in the greenhouse and fruit samples were harvested at green, white, underripe, ripe and overripe stages as described previously (Jiménez et al. 2024). Headspace VOC analysis via SPME-GC-MS identified 23 volatile terpenes, the majority of which matched compounds identified in field-grown, ripe fruits (Fig. 8). Consistent with field-grown fruit, linalool **1** was the most abundant terpene, followed by nerolidol **12**, *cis*/*trans*-linalool oxide **57/58**, and β-pinene **8**. During fruit ripening of “Royal Royce” berries, accumulation of linalool **1** and nerolidol **12** were the dominant changes with a 677-fold increase in linalool and a 2,367-fold accumulation of nerolidol **12** during the development from green to ripe fruits (Data S2). This accumulation was followed by a decrease of both metabolites as evidenced by lower linalool **1** and nerolidol **12** abundances in overripe fruits. “Mara des Bois” fruits displayed a similar pattern, with a 709-fold- increase in linalool **1** and a 2,798-fold increase in nerolidol **12** during fruit ripening. While “Mara des Bois” fruit contained a less diverse terpene blend when compared with “Royal Royce,” both cultivars showed low terpene abundance in green and white fruit. “Royal Royce” fruit showed an increase in linalool **1** as well as linalool oxide **57/58** and β-pinene **8** at the underripe stage. In ripe fruit, monoterpene abundance except for linalool **1** was lower, while an increase in nerolidol **12** was observed. This trend continued in overripe fruit that showed a higher abundance in several sesquiterpenes. Although lower in terpene abundance, a similar pattern was observed in “Mara des Bois” berries with an increase in linalool **1** and nerolidol **12** in ripe and overripe fruit.

Transcriptome analysis of the same fruit tissues revealed that known rate-limiting genes of the MEP pathway, eg, *deoxyxylulose 5-phosphate synthase* (*dxs*) and *deoxyxylulose 5-phosphate reductoisomerase* (*dxr*), showed highest transcript abundance in green fruit in either cultivar with a continued decrease in gene expression during ripening (Fig. S17).

In addition, *4-hydroxy-3-methylbut-2-enyl diphosphate reductase 1 (hdr1)* and *hdr3* showed slightly higher expression in “Mara des Bois” fruits through ripening whereas *hdr2* and *hdr4* expression gradually increased in “Royal Royce” fruits during ripening with the highest normalized counts detected in overripe fruits. By contrast, *hmg-coa reductase* (*hmgr*), as a key rate-limiting gene of the MVA pathway, increased in transcript abundance during ripening with highest expression levels in overripe fruit. Gene expression patterns of TPSs did not show a clear correlation with terpene abundance (Fig. 8). However, a moderate increase in the transcript abundance of the NESs, *FaTPS17/NES1* and *FaTPS18/NES1t*, was observed during fruit ripening in both cultivars that is aligned with an accumulation of linalool **1** and nerolidol **12**. In addition, expression of *FaTPS50* was high in white “Royal Royce” fruit possibly associated with the increase in β-pinene **8** in underripe fruit. Notably, gene expression of *FaCPS1-4* and *FaKSL1-4* was highest in green fruit of “Royal Royce” and/or “Mara des Bois.” In contrast, transcript abundance of *FaCPS5* with a proposed function in specialized diterpenoid metabolism was highest in overripe “Mara des Bois,” but not “Royal Royce,” fruit.

## Discussion

Species-specific blends of volatile terpene metabolites serve as chemical cues in various plant-environment interactions, including roles in long-distance chemical defenses, plant-microbiome interactions, and pollinator interactions (Tholl 2015). Beyond their physiological importance, terpene VOCs are core constituents of aroma traits in many fruit crops including strawberry (Nieuwenhuizen et al. 2013; Ilc et al. 2016; Ulrich and Olbricht 2016; Fan et al. 2021b). Leveraging advanced omics and synthetic biology approaches now enables a detailed investigation of the diversity of crop aroma and the underlying gene and pathway networks. The characterization of the strawberry TPS gene family provides a genomic atlas for the biosynthesis of aroma-relevant terpenes. The presence of 75 full-length *TPS* gene candidates in the octoploid *F.* × *ananassa* “Royal Royce” genome (FaRR1) places the strawberry TPS family at the higher end of the TPS families identified in other species of the rosid clade, including eucalyptus (*Eucalyptus grandis*) with 113 TPSs (Külheim et al. 2015), grape (*Vitis vinifera*) with 39 TPSs (Martin et al. 2010), apple (*Malus domestica*) with 55 TPSs (Nieuwenhuizen et al. 2013), and almond (*Prunus dulcis*) with ∼30 predicted TPSs (Nawade et al. 2019). Notably, the diploid genome of the *F. vesca* progenitor contains a smaller TPS family of 43 predicted members (Zhang et al. 2022), 25 of which have orthologs in the octoploid FaRR1 genome. Additionally, the presence of TPSs (ie, *FaTPS3-5*, *11-15*, *25*, *33*, *41-45*, *53*, and *63*) in the FaRR1 genome that lack syntenic equivalents in *F. vesca* support the expansion of the TPS gene family in octoploid strawberry through a combination of polyploidization and tandem gene duplication events (Fig. 1). For instance, closely co-localized TPS genes are present on the distal ends of chromosome 3 homoeologs, including the β-farnesene synthases *TPS6*, *TPS7*, and *TPS10*; the NESs *FaTPS17/NES1* and *FaTPS18/NES1t*, as well as *FaTPS11/NES2* and *FaTPS12-14* co-located on chromosome 3B, suggesting that they arose through repeated gene duplication events.

**Figure 9:**
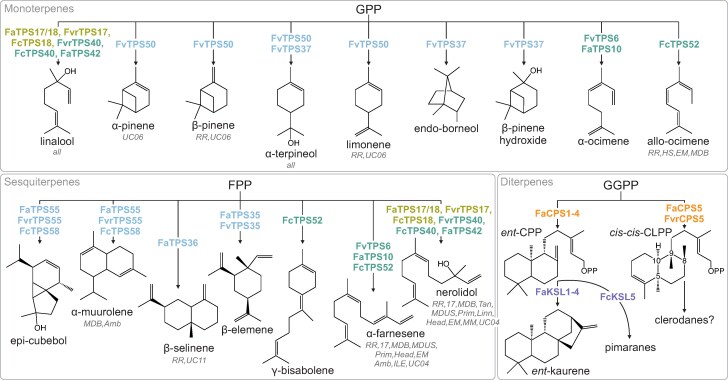
Current model of the strawberry terpene-metabolic network. Shown are major terpene products formed by the 27 TPS enzymes functionally analyzed in this study. TPS enzymes are colored corresponding to TPS clades assigned in Fig. 2. GGPP, geranylgeranyl diphosphate; GPP, geranyl diphosphate; FPP, farnesyl diphosphate; CPP, copalyl diphosphate; CPS, copalyl diphosphate synthase; KSL, kaurene synthase-like; RR, “Royal Royce”; 17, “17C224P011”; MDB, “Mara des Bois”; MDUS, “MDUS 5130”; Tan, “Tangi”; Prim, “Primella”; Head, “Headliner”; EM, “EarliMiss”; Amb, “Ambato”; ILE, “Isle de Lemuy 02A White”; HS, Harris Springs’; MM, “Madame Moutot” (MM).

In addition to the previously reported NESs, FaTPS17/NES1, FaTPS18/NES1t, and FaTPS11/NES2, biochemical characterization showed that TPS40 and FaTPS42 also form nerolidol **12** and linalool **1** (Figs. 3 and 4). However, TPS40 and FaTPS42 are phylogenetically distant from known NES enzymes within the TPS-b family (Fig. 2), highlighting that the strawberry genome contains a larger group of NESs (Fig. 9), thus providing genetic redundancy for the biosynthesis of these major terpenes in strawberry and likely contributing to the high abundance of these aroma compounds in most strawberry cultivars and accessions (Fig. 7). Previous studies showed a shared QTL on chromosome 3 for several aroma terpenes such as linalool **1**, α-farnesene **13**, and nerolidol **12**, which contained *FaTPS17/NES1*, thus supporting a major role in strawberry aroma metabolism (Barbey et al. 2021). However, a higher gene expression of especially *FaTPS40* and *FaTPS42* in early cultivars and wild relatives when compared with modern cultivars detected in this study, supports that additional TPSs contribute to nerolidol **12** and linalool **1** accumulation (Fig. 7). Interestingly, subcellular localization prediction supports a plastidial localization of TPS17 and TPS18, whereas TPS40 and TPS42 are predictably cytosolic, further suggesting that these TPS contribute differently to linalool **1** and nerolidol **12** biosynthesis at the cellular level.

Beyond the identification of additional NES enzymes, biochemical analysis of 20 mono- and sesqui-TPSs selected for their predicted functions and expression in strawberry fruits revealed the detection and biosynthesis of 47 terpene metabolites (Fig. 9), including known strawberry terpenes and metabolites that, to the best of our knowledge, have not previously been reported in strawberry such as α- **26** and δ-guaiene **65**, α-muurolene **28**, β-selinene **53**, and eremophilene **64**, while volatile profile analysis of field grown fruits newly detected isopulegol **63**, 7-epi-α-selinane **67**, *trans*-pinocarvyl acetate **54**, and 4,11-selinadiene **62** (Figs. 4, 7, 9, and S15). The identified TPS products reveal the biosynthetic origin of over half of the terpene metabolites detected in the strawberry accessions analyzed in this study (Fig. 7), notably including linalool **1**, α-terpineol **2**, limonene **9**, α-/β-pinene **7/8**, terpinen-4-ol **11**, allo-ocimene **59**, nerolidol **12**, α-farnesene **13**, caryophyllene **25**, humulene **22**, α-/δ-guaiene **26/65**, β-selinene **53**, eremophilene **64**, and α-muurolene **28**. Notably, some TPS enzymes, such FaTPS35, FvTPS35, and FaTPS6 showed differences in their product profile when tested in vitro and in vivo. Presumably, additional products observed in in vivo co-expression assays result from a combination of possible factors, including differences in protein expression, substrate availability, and conversion by endogenous *E. coli* enzymes. The functional identification of *FvTPS6*, *FaTPS7*, and *FaTPS10* as genes encoding β-farnesene synthases that form a syntenic grouping with *FaTPS21* in the FaRR1 genome provides biochemical evidence for previously reported functional predictions on the basis of eQTL analyses (Barbey et al. 2021). Although only limited correlation of individual TPS gene expression with terpene abundance was detected (Fig. 8), presence of *FvTPS6*, *FaTPS7*, and *FaTPS10*, alongside *FaTPS21* and *FaNES1/TPS17*, on the shared QTL on chromosome 3 for linalool **1**, nerolidol **12**, and α-farnesene **13** among other terpenes (Barbey et al. 2021), indicates a functional role of these TPSs in producing these aroma metabolites. In addition to FaTPS6, 7 and 10, FcTPS52, identified in *F. chiloensis* “Isle de Lemuy,” also produced α-farnesene **13** but represented a multi-product TPS also forming other sesquiterpenes such as β-acoradienol **15**, α-farnesene **13**, (*E*)-γ-bisabolene **17**, thus possibly contributing to the diversity of strawberry aroma profiles (Figs. 4 and S11).

Production of α-pinene **7** and several low abundant byproducts by FvTPS50/PINS confirms earlier studies that demonstrated the function of FvTPS50/PINS, and a downstream-acting cytochrome P450 monooxygenase and alcohol acyltransferase, in the biosynthesis of myrtenol and myrtenyl acetate (Aharoni et al. 2004). Transcript and metabolite profiling of the diploid accession “UC06” showed significant correlation between high *FaTPS50* transcript abundance and accumulation of α-/β-pinene **7/8**, myrtenol **55**, and myrtenyl acetate **61**, suggesting that this pathway is particularly productive in this accession. In addition to FvTPS50/PINS, formation of linalool **1**, α-terpineol **2**, and several other monoterpenes by FvTPS37 represents another enzymatic source of consumer-preferred aroma metabolites in strawberry. Differential gene expression of *FvTPS37* across the breeding material may contribute to differences in terpene aroma metabolism across different accessions (Figs. 6 and 7). In addition, production of as many as 23 different sesquiterpene products by FaTPS35, FaTPS36, TPS55, and FcTPS58 reveals the genetic basis for the biosynthesis of more complex terpene blends in some of the tested accessions such as *F. virginiana “*UC11” and “NC_96-35-2” and diploid “UC06” (Fig. S12). Collectively, the identified TPS functions demonstrate that catalytic redundancy and functional diversity within the strawberry TPS family provide the biochemical basis for the complex aroma profiles in different cultivars and accessions, especially in wild and diploid strawberries that often feature a higher diversity of terpenes (Aharoni et al. 2004; Bianchi et al. 2014; Negri et al. 2015; Ulrich and Olbricht 2016; Urrutia et al. 2017; Duan et al. 2018).

In addition to a large family of mono- and sesqui-TPS enzymes, genome mining identified 10 diTPS candidate genes in the FaRR1 genome (Fig. 9). Biochemical analysis of FaCPS1-4 showed enzyme catalytic redundancy also in strawberry diterpenoid metabolism with all enzymes producing *ent*-CPP **42**, as well as *ent-*kaurene **43** in the pairwise activity with the Class I diTPSs FxaKSL1-2, thus suggesting functions in GA phytohormone metabolism as well as possibly specialized diterpenoid pathways similar to related enzymes identified in other plant species (Peters 2010; Karunanithi and Zerbe 2019). By contrast, FvrCPS5 “NC_96-35-2” and FaCPS5 “Royal Royce” were predicted as specialized diTPSs and indeed produced *cis-cis*-clerodienyl diphosphate (CC-CLPP) **44**, a member of the group of clerodane diterpenoids that are abundant in species of mint and a few monocot crops where they have probable functions in stress response mechanisms (Peters 2010; Pelot et al. 2017; Johnson et al. 2019a, 2019b; Muchlinski et al. 2021; Tiedge et al. 2022). Co-localization of *FaCPS5* with a predicted Class I diTPS, *FaKSL4*, on chromosome 3D suggested a possible pairwise activity of the encoded enzymes in clerodane biosynthesis. However, co-expression studies in *E. coli* did not reveal any coupled reaction products, thus requiring more expansive future studies to elucidate the diterpenoid-metabolic network in strawberry.

In addition to the terpene diversity across different strawberries, expectedly, alterations in terpene metabolism were also observed during fruit ripening. Consistent with their dominance in most tested accessions, linalool and nerolidol accumulated throughout fruit ripening (Fig. 8). High gene expression of the NESs *FaTPS17/NES1* and *FaTPS18/NES1t* in underripe and ripe fruits indicates a likely involvement of these genes in driving metabolite production during fruit ripening. Likewise, increase in linalool oxides **57/58**, limonene **9**, myrtenol **55** and β-pinene **8** in green or white “Royal Royce” fruits correlated with increasing transcript abundance of *FaTPS50*, supporting a possible biosynthetic relationship. Furthermore, high transcript abundance of the predicted *ent*-CPP synthases, *FaCPS1-4*, in green fruit suggests possible functions in GA phytohormone biosynthesis at the onset of fruit development and ripening (Csukasi et al. 2011; Liao et al. 2018). However, given the specialized function of FaCPS5 and the role of *ent*-CPP as a specialized diterpenoid precursor in other plant species (Peters 2010; Pelot et al. 2017; Johnson et al. 2019a, 2019b; Muchlinski et al. 2021; Tiedge et al. 2022), possible roles in stress response mechanisms in developing fruits cannot be excluded.

Much diversity in both comparative abundance and complexity of terpene profiles exists across the diversity panel analyzed in this study as exemplified by linalool amounts varying by a factor of more than 500 between the highest and lowest linalool **1** producers. Congruent with past research, terpene-derived fruit aromas in *F.* × *ananassa* accessions are dominated by linalool **1**, nerolidol **12**, and α-terpineol **2**, whereas wild diploid accessions are characterized by olefinic monoterpenes such as α-/β-pinene **7/8** (Aharoni et al. 2004; Chambers et al. 2012, Fig. 7). This reflects recent breeding efforts having selected for high linalool **1** cultivars based upon known positive correlations with consumer preferences (Schwieterman et al. 2014; Ulrich and Olbricht 2016; Fan et al. 2021a). Similarly, significant differences in nerolidol **12** abundance were detected between most non-*F.* × *ananassa* accessions and more modern breeding lines such as “EarliGlow” and “17C224P001” (Data S1). The functional characterization of previously reported and unidentified TPSs provides a better understanding of the functional range of the strawberry TPS family and its contribution to terpene variation across different cultivars and accessions, thus providing a resource to link biochemically verified gene functions with rapidly expanding genomic and genetic insights into the metabolic network driving terpene aroma profiles. A broader knowledge of the metabolic diversity across the existing germplasm may further aid the identification of strawberry lines for breeding of advanced, consumer-preferred strawberry aromas.

## Materials and methods

### Plant material

A panel of 96 strawberry (*F*. × *ananassa*) accessions comprising a selection of both ecotypes and modern cultivars derived from the UC Davis Strawberry Breeding Program collection were grown under established commercial conditions at Wolfskill Experimental Orchards in Winters, California in 2021 and UC Davis Vegetable Crop Fields in Davis, CA in 2022 (Table S10, Petrasch et al. 2022). Ripe fruits from each accession were harvested between 8 and 10 AM throughout the growing season every 2 to 3 wk between April and June and stored at 4 °C with 70% relative humidity until processing. Twice in early May during the middle of the 2021 season, additional ripe fruits from 17 accessions which span the range of strawberry domestication and were expected to harbor a diversity of volatile profiles were harvested for transcriptome analysis (Table S5). Upon harvest, berries were immediately sliced, frozen in liquid N_2_, transported on dry ice, and stored at −80 °C until use for metabolite and transcriptome analysis. In addition to field harvests, 2 cultivars, “Royal Royce” and “Mara de Bois,” were grown at UC Davis greenhouses. Two plants of each cultivar were planted into pots in Fall 2020 and kept throughout the winter so that berries were harvested as needed throughout the first week of March 2021 through the first week of June 2021. Fruits were harvested fully green, white, turning or underripe, fully ripe, and overripe and processed as described above for further transcriptomic and aroma analysis (Jiménez et al. 2024).

### Metabolite analysis

For metabolite profiling of field-grown plants, 3 to 9 fully ripe berries (depending on different fruit production across the tested accessions) were harvested and pooled from 4 individual plants of each accession. Berries were then divided into 3 technical replicates and halved for subsequent metabolite or transcriptome analysis. For metabolite analysis, the samples were weighed and supplemented with an equal amount (w/v) of 20% NaCl and 50 pp γ-undecalactone (final concentration, in methanol; Sigma-Aldrich, St. Louis, MO, USA) as an internal standard (Jetti et al. 2007). Samples were homogenized in a blender (SharkNinja, Needham, MA, USA) and 5 mL of the solution transferred to pre-weighed 20 mL glass crimp-cap SPME vials (Agilent Technologies, Santa Clara, CA, USA). Samples were capped, labeled, and stored at −20 °C.

Samples were analyzed using solid-phase microextraction-gas chromatography-mass spectrometry (SPME-GC-MS) on an Agilent Intuvo 9000 GC coupled with a 5977b MS with a 350 XTR Electron Ionization (EI) source (Agilent Technologies). Samples were incubated at 30 °C for 5 min during SPME fiber adsorption with a 50/30 *µ*m DVB/Carboxen-WR/PDMS SPME fiber (Supelco, Bellefonte, PA, USA), followed by injection and 6 min desorption into the inlet at 240 °C using helium as a carrier gas at a flow rate of 1.2 mL/min. Compound separation was achieved on a DB-WAX UltraInert column (30 m × 0.25 mm × 0.25 *µ*m column; Agilent Technologies). Oven settings were held at 40 °C for 2 min, 4 °C/min to 70 °C, 6 °C/min to 180 °C, 15 °C/min to 220 °C, and held at 220 °C for 2 min. Mass ranges were recorded from 40 to 400 *m*/*z* with an EI energy of 70 eV and detector temperature set to 230 °C. Compounds were identified based on available authentic standards (Table S8) or matches to reference mass spectral databases of the National Institute of Standards and Technology (NIST, version 17.1). Where product identification on the basis of authentic standards was not possible, Kovats indices were calculated. Compound abundance was calculated based on relative peak areas and integration using MassHunter Unknowns Analysis software with default deconvolution settings (Agilent Technologies). Metabolite abundance values were normalized against the internal standard and sample dry weight after drying samples for 48 h at 100 °C, and subtraction of saline buffer content and vial weight. The same total ion chromatograms were secondarily analyzed against 8 terpene standard curves and normalized against the same dried berry weights used above to report compound concentrations in nanograms per gram dry berry weight. Relative quantification of 8 quantified metabolites were performed using MassHunter Quantification and Unknown Analysis software (Agilent Technologies) and are shown in Table S8 with equations supplied for linear regression calibration curves for all 8 standards following previous protocols (Jetti et al. 2007). Cleaned, normalized GC-MS data were analyzed using the randomForest package (Liaw and Wiener 2002) in the R Statistical Software v.4.4.0 (R Core Team 2021) with default parameters of ntree 5000. The square root of total variables entered were tested at each split. Thus, for the analysis of all 171 detected VOCs 13 randomly chosen aroma metabolites were tested at each split. Mean decreased accuracy, or the level of accuracy lost at each tree node when a particular metabolite is omitted, were extracted from each random forest analysis to estimate metabolite impact within sample aroma profiles. Principal component analyses and multidimensional scaling plots were generated within the randomForest package and R Statistical Software.

### RNA isolation and transcriptome analysis

The second half of harvested flash frozen berries (see above) were ground into a fine powder in liquid N_2_ using mortars and pestles. Approximately 2 g of ground tissue was mixed with 6 mL warm extraction buffer (2% v/v CTAB, 2% PVP, 100 mm Tris, 40% NaCl, 25 mm EDTA, 0.2 g spermidine, 2% mercaptothion) and incubated at 65 °C for 5 min (Blanco-Ulate et al. 2013). Equal volumes of 24:1 chloroform:isoamyl alcohol was added, mixed, and centrifuged at 4,000 rpm for 30 min at 4 °C. The supernatant was collected, supplemented with 24:1 chloroform:isoamyl alcohol and centrifuged again. The supernatant was then supplemented with 10% 1 M Potassium Acetate, centrifuged at 4,000 rpm for 20 min at 4 °C, and the resulting supernatant added to a ¼ volume of 10 M Lithium Chloride. The sample was mixed gently and precipitated overnight at −20 °C before centrifugation at 4,000 rpm for 45 min at 4 °C. The resulting pellet was resuspended in 60 *µ*L RNAse-free water and cleaned using the Zymo Research Quick-RNA Mini-Prep Kit and DNase1 treatment (Zymo Research Corporation, Irvine, CA, USA) as per manufacturer's instructions.

Library preparation and transcriptome sequencing was performed by the NovoGene Corporation Inc. using NovaSeq 6000 paired-end short read sequencing using the NEBNext and AMPure XP suite of reagents and protocols (NEB, USA). Transcripts were cleaned using the HTStream suite to remove adapters, polyA tails, and reads under 50 bp. FastQC and MultiQC were used to assess RNA read quality before proceeding (Andrews 2010). The remaining high-quality reads (20 to 29.6 M 150 bp reads with an average of ∼24 M reads x2 per sample) were aligned to the FaRR1 genome and read counts were obtained using the STAR package with default parameters (Dobin et al. 2013; Petersen et al. 2015). Additionally, to identify transcripts that did not align to the FaRR1 genome, a Trinity de novo assembly (Grabherr et al. 2011; Haas et al. 2013) was performed with default parameters and mapped to the assembled de novo transcriptomes using bowtie2 for all reads of each genotype (Langmead and Salzberg 2012). Cleaned reads were normalized using the TMM function with default parameters in the edgeR software package (Robinson et al. 2010) and averaged across replicates for each genotype, after which any contigs or genes with fewer than 10 counts for at least one sample were discarded. Cleaned de novo transcripts were also compared with the FaRR1 genome to distinguish any transcripts that had <95% sequence identity with any of the 75 full-length FaRR1 genomic TPS candidates (Tables S3 and S4). Gene counts were normalized and analyzed in R using the edgeR (Robinson et al. 2010) and heatmap (Perry 2024) packages.

### Gene identification, synthesis, and construct design

To identify TPS gene candidates and core genes of the MEP and MVA pathways, reciprocal BLAST searches were performed on the FaRR1 genome (Hardigan et al. 2021a) and the transcriptome data generated in this study against a curated database of protein sequences that represent the functional space of TPSs and MEP/MVA enzymes across higher plants (Zerbe et al. 2013). The obtained transcripts were manually curated for presence of characteristic catalytic domains, sequence completeness, and phylogenetic relationships as described previously (Zerbe et al. 2013). The resulting 104 possible TPS were narrowed further based on sequence length: mono- and sesqui-TPS candidates with a length of 450 to 750 amino acids and diTPS with a length of 550 to 850 amino acids were selected for further analysis, resulting in 27 full-length TPS genes (Table S4). Transcripts featuring expression counts lower than 10 or lacking de novo transcripts were also not considered for further study.

Full-length or N-terminally truncated (removal of the predicted plastidial transit peptides; Table S4) genes were obtained via DNA synthesis and codon optimized for expression in *E. coli* (Twist Biosciences, South San Francisco, CA, USA). For protein purification and in vitro enzyme assays, TPS genes were inserted into the pET28a(+) expression vector (EMD Millipore, Burlington, MA, USA) in frame with the N-terminal poly-His tag. For in vivo co-expression analysis in *E. coli*, synthesized TPS genes were inserted into the pACYC vector (EMD Millipore) containing previously confirmed functional copies of either *A. grandis* (Grand Fir) GPP synthase, *Z. mays E*,*E*-FPP synthase (*ZmFPPS4*), or the *A. grandis* GGPP synthase in the second multiple cloning site.

### In vitro enzyme assays

Synthesized TPS constructs in the pET28a(+) vector were expressed in *E. coli* BL21DE3-C41 cells (Sigma-Aldrich) and Ni^2+^-NTA affinity purified as follows: 100 mL cultures were induced with 1 mm isopropyl-β-D-1-thiogalactopyranoside (IPTG; Sigma-Aldrich) and grown for 24 h at 16 °C. Cultures were pelleted at 4 °C and resuspended in 40 mL of wash buffer (20 mm Tris Base, 50 mm KCl, pH 7) then resuspended in 4 mL of phosphate-buffered saline lysis buffer (50 mm H_2_NaPO_4_, 200 mm NaCl, 10% glycerol, 15 mm Imidazole, pH 7) with freshly added 0.5 mm phenylmethylsulfonyl fluoride (Sigma-Aldrich), and 1 mm dithiothreitol (DTT; Sigma-Aldrich) and sonicated in an ice bath at 20% amplitude for a total of 2 min. Crude lysates were pelleted and stored at −80 °C. Protein was purified at 4 °C using equilibrated 750 *µ*L Ni-NTA agarose. Lysates were added to Ni-NTA beads with 4 mL of lysis buffer and batch bound for 1 h. Lysates were allowed to settle for 30 min before they were washed with 2 mL of wash buffer 1 (50 mm H_2_NaPO_4_, 300 mm NaCl, 5% glycerol, 25 mm Imidazole, pH 7) and followed with 4 mL of wash buffer 2 (50 mm H_2_NaPO_4_, 300 mm NaCl, 5% glycerol, 35 mm imidazole, pH 7). Protein was eluted using Elution Buffer (50 mm H_2_NaPO_4_, 300 mm NaCl, 350 mm Imidazole, pH 7). Presence of all purified proteins were confirmed via Western Blot using the BioRad TransBlot and Alkaline-Phosphatase system from BioRad Technologies (Hercules, CA, USA) and Alkaline Phosphatase Anti-6x His tag antibody (AD1.1.10; Abcam Limited, Cambridge, UK).

Single-vial mono- and sesqui-TPS enzyme assays were performed as previously described (Martin et al. 2004). In brief, 100 *μ*g of recombinant proteins were combined with 15 *μ*m GPP or *E*,*E*-FPP (Sigma-Aldrich) as substrates in a total volume of 1 mL of assay buffer (25 mm HEPES, pH 7.2, 100 mm KCl, 10 mm MnCl_2_, 10% glycerol, 5 mm DTT) and overlaid with 600 *µ*L of hexane (Thermo Fisher Scientific) to trap terpene products during the assays. Each sample was supplanted with 0.03 ppm isobutylbenzene (Sigma-Aldrich) as internal standard. After 2 h incubation at room temperature, enzyme products were extracted by vortexing and analyzed via GC-MS as described below.

### In vivo enzyme co-expression analysis

For co-expression assays of selected TPSs, *E. coli* BL21DE3-C41 cells were co-transformed with individual TPS constructs in the pACYC vector, as well as plasmids carrying either *A. grandis* GPP synthase, *Z. mays* FPP synthase (*ZmFPP4*), or the *A. grandis* GGPP synthase (pGG plasmid) and key enzymes of the MEP pathway (pIRS plasmid) as described previously (Cyr et al. 2007; Morrone et al. 2010). For combinatorial expression of diTPSs, Class I diTPSs were cloned into the pET28a(+) plasmid and Class II diTPSs were inserted into the pACYC vector to maintain replication complementarity. For all combinations, co-expression assays were performed as described previously (Pelot et al. 2017). In brief, cultures were grown at 37 °C and 180 rpm in 50 mL of Terrific Broth medium to an OD_600_ of ∼0.6 before cooling the cultures to 16 °C and induction with 1 mm IPTG for 72 h with the addition of 1.25 mL 1 M Sodium pyruvate and 50 *μ*L MgCl_2_. Products were extracted with 50 mL of hexane containing 17.5 ng/*μ*L 1-eicosene (Sigma-Aldrich) as internal standard, air dried, and resuspended in 1 mL of *n*-hexane for GC-MS analysis.

### GC-MS analysis of TPS products

Analysis of extracted enzyme products was conducted via liquid-injection GC-MS on an Agilent 7890B GC coupled with a 5977A Extractor EI MS (Agilent Technologies). Samples were injected into the inlet at 250 °C using Helium as a carrier gas at a flow rate of 1.2 mL/min. Compound separation was achieved on a HP-5MS column (30 m × 0.25 mm × 0.25 *µ*m column; Agilent Technologies). Oven settings for analysis of the mono- and sesqui-terpenes were held at 50 °C for 2 min, 8 °C/min to 200 °C, 25 °C/min to 250 °C, and held at 250 °C for 1 min. Oven settings for analysis of diterpene products were held at 50 °C for 3 min, 15 °C/min to 300 °C and held at 300 °C for 4 min. Mass ranges were recorded after a solvent delay of 6 min for mono- and sesqui-terpenes or 13 min for diterpenes with a mass range of 40 to 400 *m*/*z*, an EI energy of 70 eV, and the detector temperature set to 230 °C. Total ion chromatogram peaks were integrated using MassHunter Unknowns Analysis software (Agilent Technologies) and compounds were identified based on comparison to available authentic standards (Table S8; Sigma-Aldrich) or best matches to reference mass spectral databases of the NIST, version 17.1. Where product identification on the basis of authentic standards was not possible, Kovats indices were calculated (Table S9). For Kovats index analysis, data were collected by GC-MS as described above using a C8-C20 alkane standard mixture (Sigma-Aldrich). Kovats indices (KI) were calculated using the following formula: KI = 100*×n* + *x* (*n* = carbon number of alkene eluting immediately before the focal compound; *x* = relative position as based on retention time (RT) as per the below formula) (Vandenpool and Kratz 1963; Kovats 1965).


$$\begin{array}{rl} X\;= & 100\times (RT_{\mathrm{sample}}\text{–}RT_{\mathrm{alkene}\;\mathrm{before}\;\mathrm{sample}\;\mathrm{RT}})/\\ & \left(RT_{\mathrm{alkene}\;\mathrm{after}\;\mathrm{sample}\;\mathrm{RT}}\text{–}RT_{\mathrm{alkene}\;\mathrm{before}\;\mathrm{sample}\;\mathrm{RT}}\right) \end{array}$$

### Heat map and hierarchical cluster analysis

Gene candidates of the MEP and MVA pathways were plotted across the FaRR1 genome using Chromplot in R (Oróstica and Verdugo 2016, Table S1). Heatmaps, and hierarchical cluster analysis on selected transcriptome data was analyzed using the heatmap package in R (Perry 2024).

### Phylogenetic analysis

For phylogenetic studies, protein sequences of the identified TPSs and known TPSs from other Rosaceae and plant species were aligned using the MUSCLE package with neighbor joining parameters (Edgar 2004) and cleaned with BMGE default parameters (Criscuolo and Gribaldo 2010). A maximum likelihood phylogenetic tree was generated with the NGphylogeny.fr user interface PHYML with Smart Model Selection, Subtree Pruning and Regrafting, minimum theoretical information criterion (AIC) and 500 bootstraps (Guindon et al. 2010; Lefort et al. 2017; Lemoine et al. 2018, 2019). The tree was visualized using Newick displays (Junier and Zdobnov 2010) and formatted using Geneious (www.geneious.com).

## Supplementary Material

kiag292_Supplementary_Data

## Data Availability

The RNA-sequencing data were submitted to the National Center for Biotechnology Information Sequence Read Archive (NCBI SRA; ncbi.nlm.nih.gov/sra), BioProject: PRJNA1172127. Nucleotide sequences for functionally characterized transcripts reported in this study were submitted to the NCBI GenBank (SUB14747557) with the following accession numbers: PQ433313 (*FcKSL5*); PQ433314 (*FcTPS18*); PQ433315 (*FcTPS40*); PQ433316 (*FcTPS52*); PQ433317 (*FcTPS58*); PQ433318 (*FvTPS6*); PQ433319 (*FvTPS35*); PQ433320 (*FvTPS37*); PQ433321 (*FvTPS50*); PQ433322 (*FvrCPS5*); PQ433323 (*FvrTPS5*); PQ433324 (*FvrTPS17*); PQ433325 (*FvrTPS40*); PQ433326 (*FvrTPS55*); PQ433327 (*FaCPS5*); PQ433328 (*FaKSL1*); PQ433329 (*FaKSL2*); PQ433330 (*FaKSL3*); PQ433331 (*FaKSL4*); PQ433332 (*FaTPS7*); PQ433333 (*FaTPS10*); PQ433334 (*FaTPS17*); PQ433335 (*FaTPS18*); PQ433336 (*FaTPS35*); PQ433337 (*FaTPS36*); PQ433338 (*FaTPS42*); PQ433339 (*FaTPS55*).
