# Supplementary material for: Genome-wide discovery and characterization of terpene synthases contributing to strawberry aroma metabolism
Source: Plant Physiol. 2026 May 22;201(3):kiag292. doi: 10.1093/plphys/kiag292 (PMC13368613; doi:10.1093/plphys/kiag292)

**Supplementary Fig. S1** Genome localization of upstream genes of the MVA and MEP terpene metabolism pathway (black). *AACT, Acetoacetyl-CoA Thiolase; CMS, 4-diphosphocytidyl-2-C-methyl-D-erythritol synthase; DXR, 1-deoxy-D-xylulose 5-phosphate reductoisomerase; DXS, 1-deoxy-D-xylulose 5-phosphate synthase; HDS, 4-hydroxy-3-methylbut-2-enyl diphosphate synthase; FPPS, farnesyl diphosphate synthase; HMGR, 3-hydroxy-3-methylglutaryl-CoA reductase; HMGS, 3-hydroxy-3-methylglutaryl-CoA synthase; IDI, isopentenyl diphosphate isomerase; IDS, 4-hydroxy-3-methylbut-2-enyl diphosphate reductase; IPK, isopentenyl monophosphate kinase; PT, Prenyltransferase (Geranyl Diphosphate Synthase/Geranylgeranyl diphosphate synthase).*

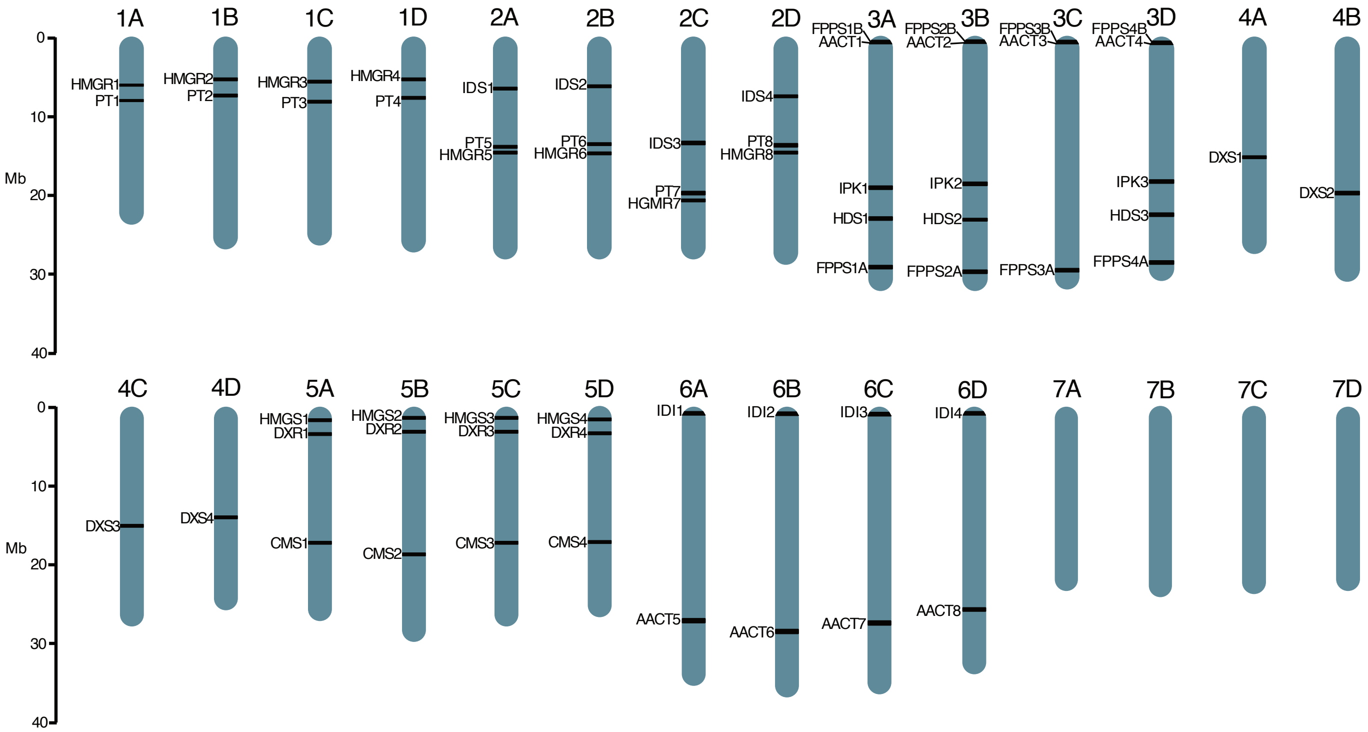

**Supplementary Fig. S2** Prenyltransferase gene synthesis analysis. Microsynteny plots of diploid *F. vesca* and FaRR1 Royal Royce of (**A-D**) prenyltransferases, (**E**) Isopentenyl diphosphate isomerase and (**F**) Isopentenyl monophosphate kinase. Genes of interest are highlighted in red.

**A** Geranyl/Geranylgeranyl Diphosphate Synthase

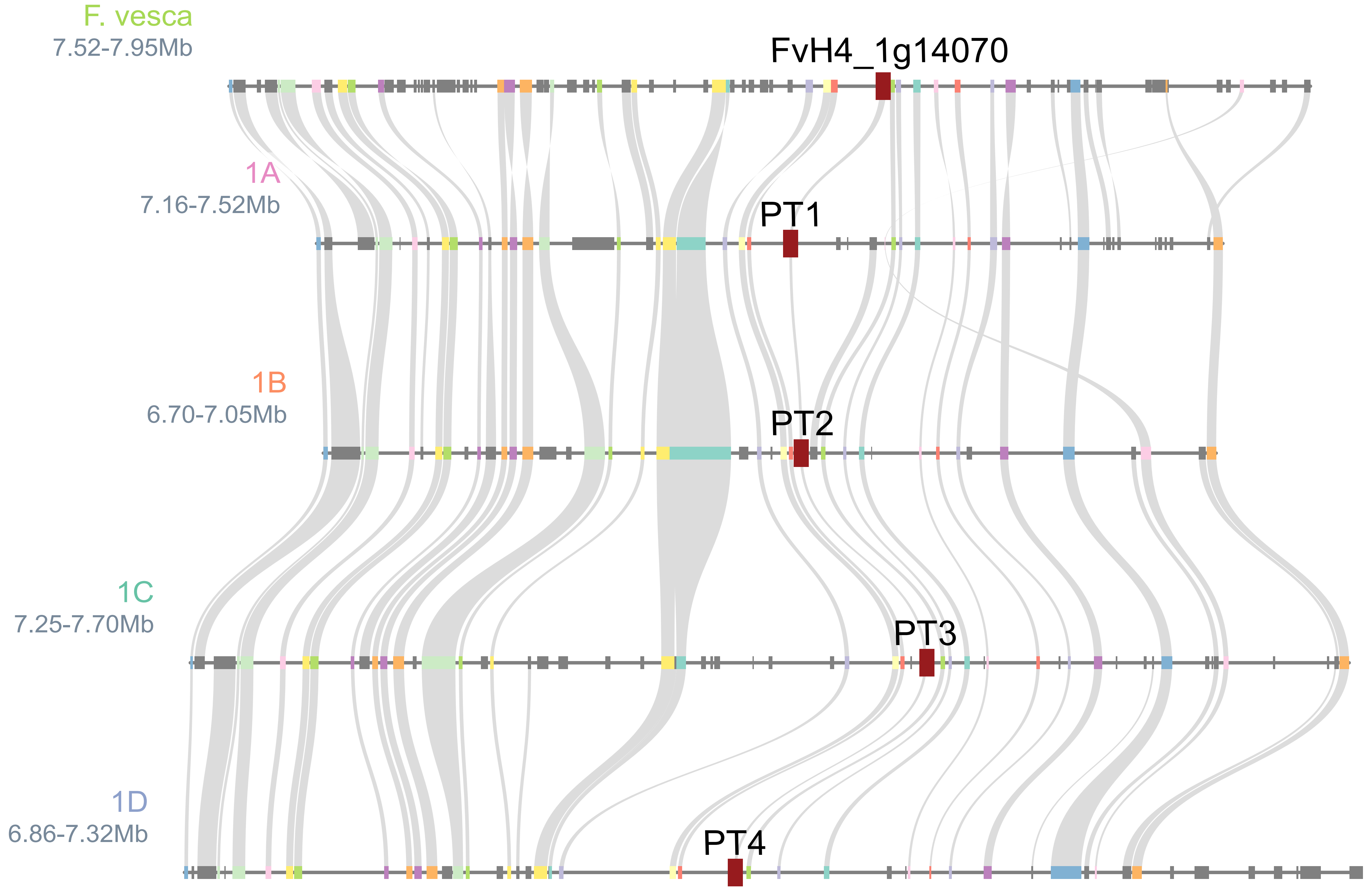


**B** Geranyl/Geranylgeranyl Diphosphate Synthase

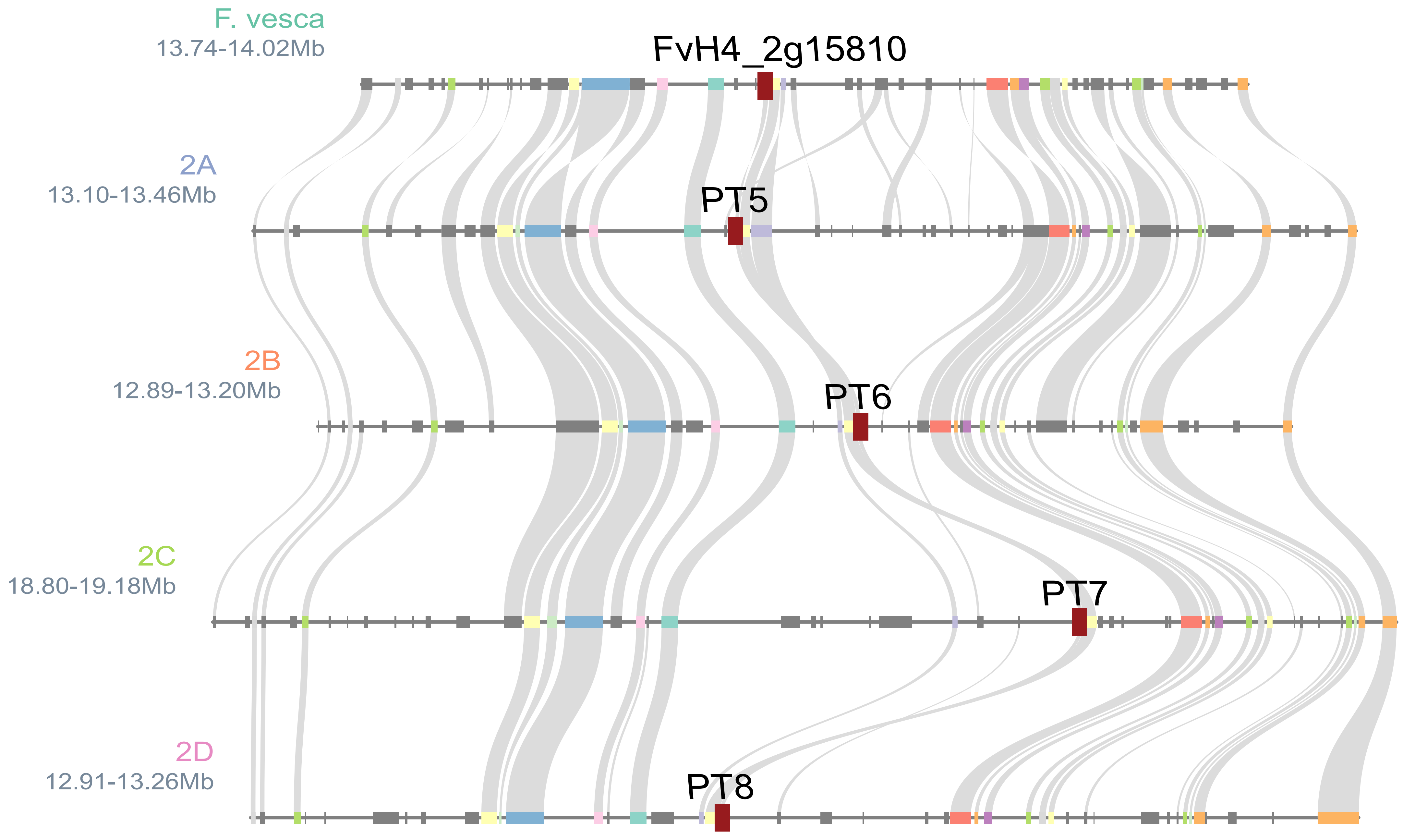

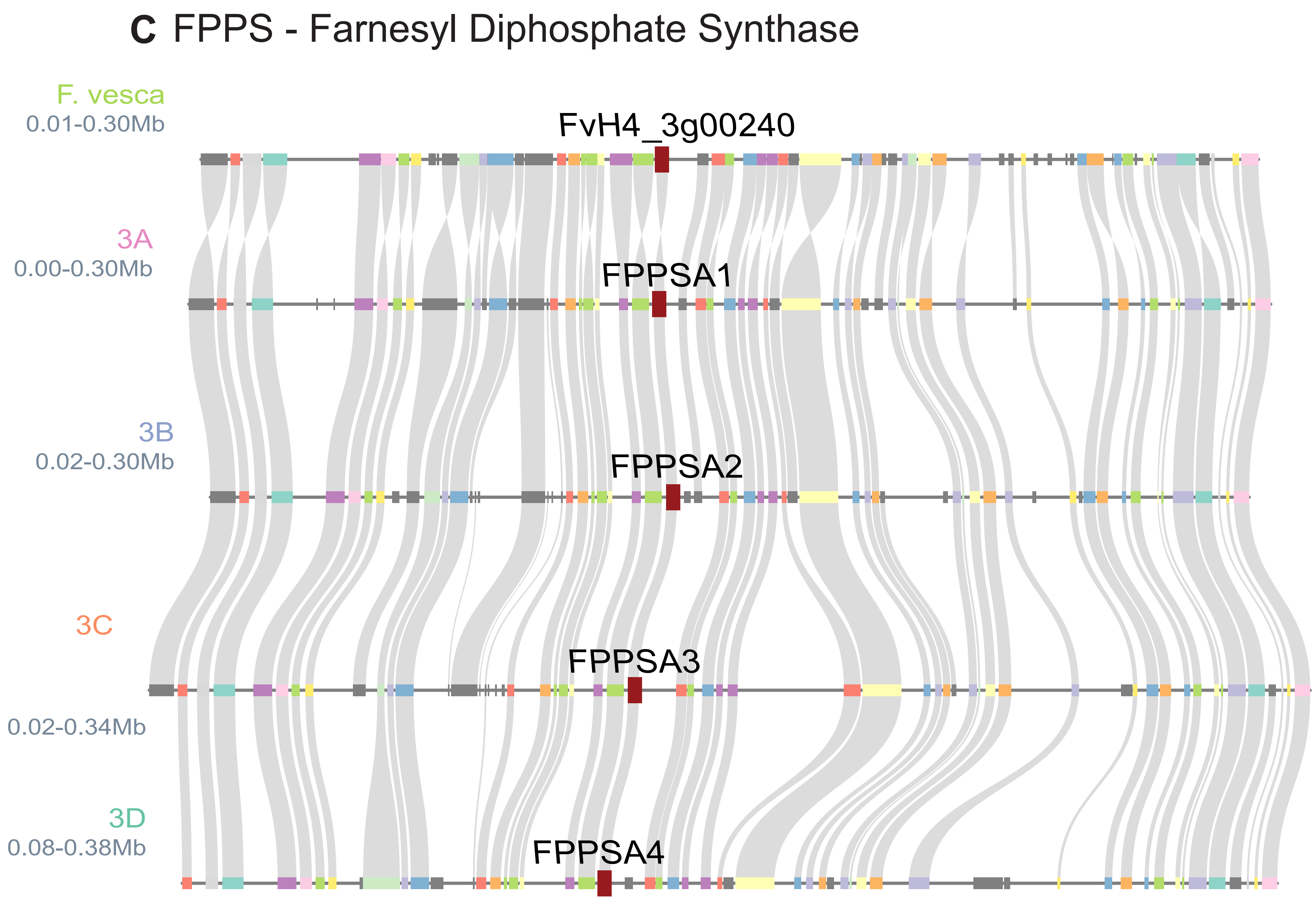
C FPPS - Farnesyl Diphosphate Synthase
F. vesca
0.01-0.30Mb
FvH4_3g00240
3A
0.00-0.30Mb
FPPSA1
3B
0.02-0.30Mb
FPPSA2
3C
0.02-0.34Mb
FPPSA3
3D
0.08-0.38Mb
FPPSA4

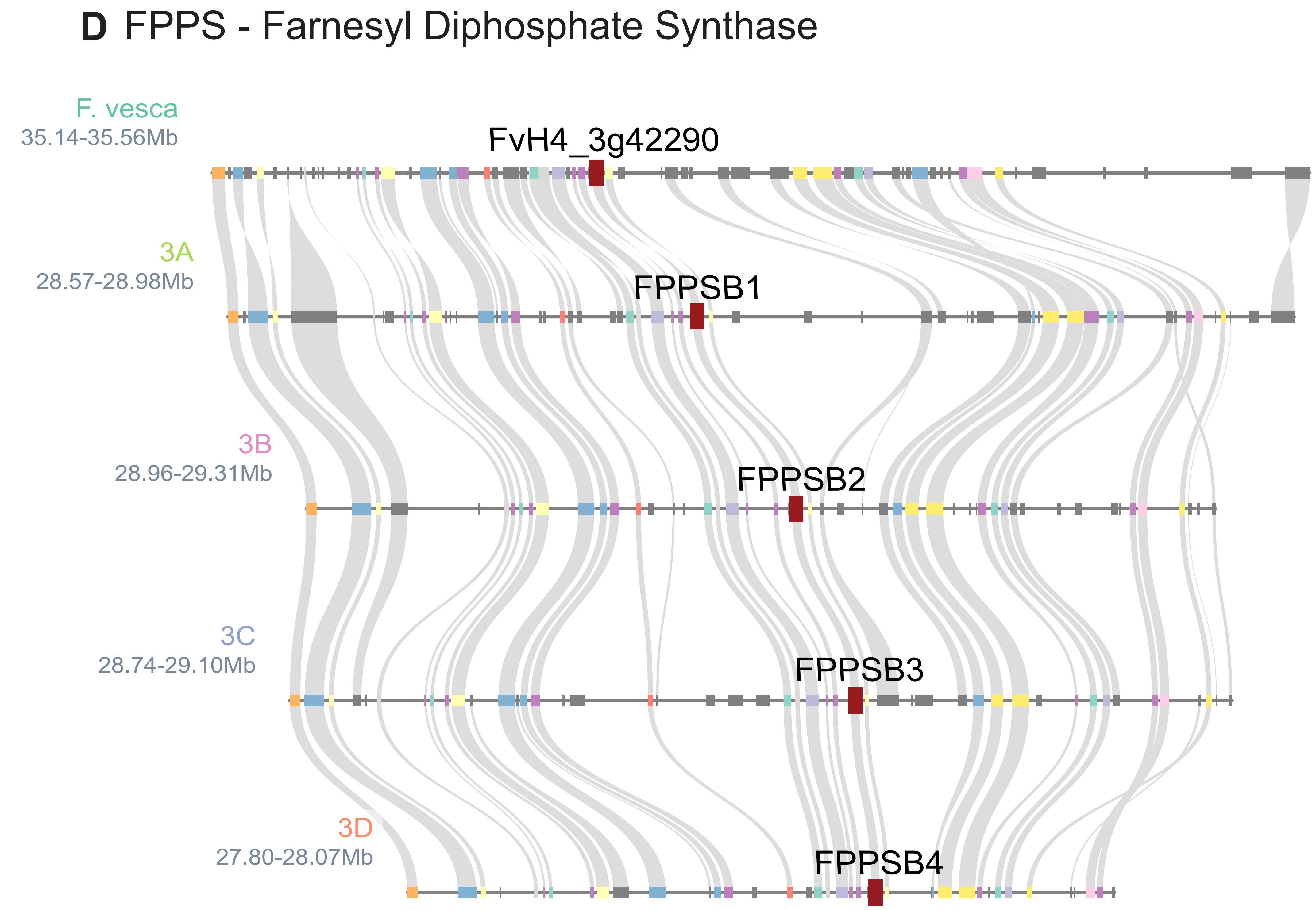
D FPPS - Farnesyl Diphosphate Synthase
F. vesca
35.14-35.56Mb
FvH4_3g42290
3A
28.57-28.98Mb
FPPSB1
3B
28.96-29.31Mb
FPPSB2
3C
28.74-29.10Mb
FPPSB3
3D
27.80-28.07Mb
FPPSB4

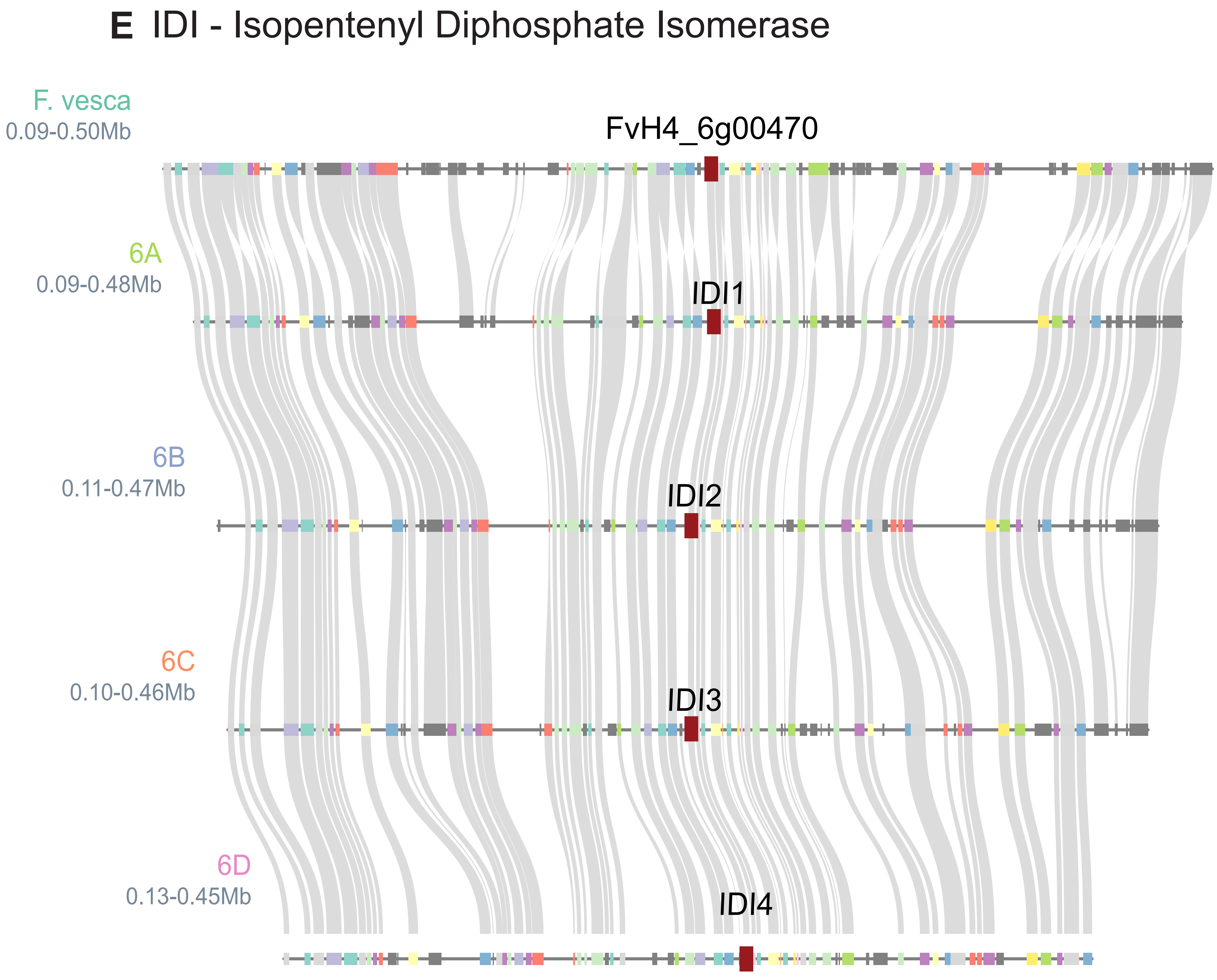
E IDI - Isopentenyl Diphosphate Isomerase
F. vesca
0.09-0.50Mb
FvH4_6g00470
6A
0.09-0.48Mb
IDI1
6B
0.11-0.47Mb
IDI2
6C
0.10-0.46Mb
IDI3
6D
0.13-0.45Mb
IDI4

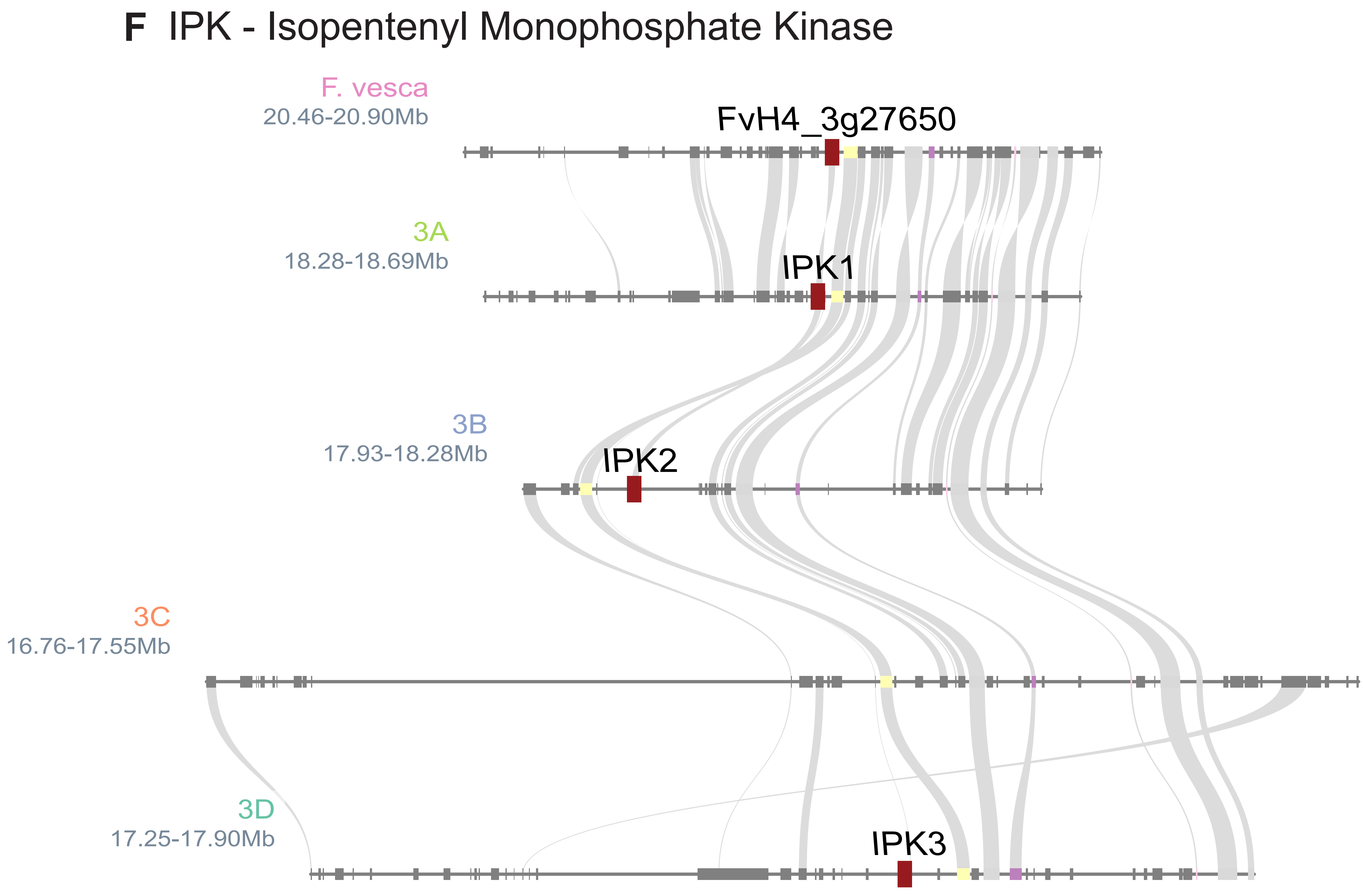
F IPK - Isopentenyl Monophosphate Kinase
F. vesca
20.46-20.90Mb
FvH4_3g27650
3A
18.28-18.69Mb
IPK1
3B
17.93-18.28Mb
IPK2
3C
16.76-17.55Mb
3D
17.25-17.90Mb
IPK3

**Supplementary Fig. S3** Microsynteny plots of diploid *F. vesca* and FaRR1 Royal Royce of MEP pathway genes. Genes of interest are highlighted in red.

**A** DXS - 1-deoxy-D-xylulose 5-phosphate synthase

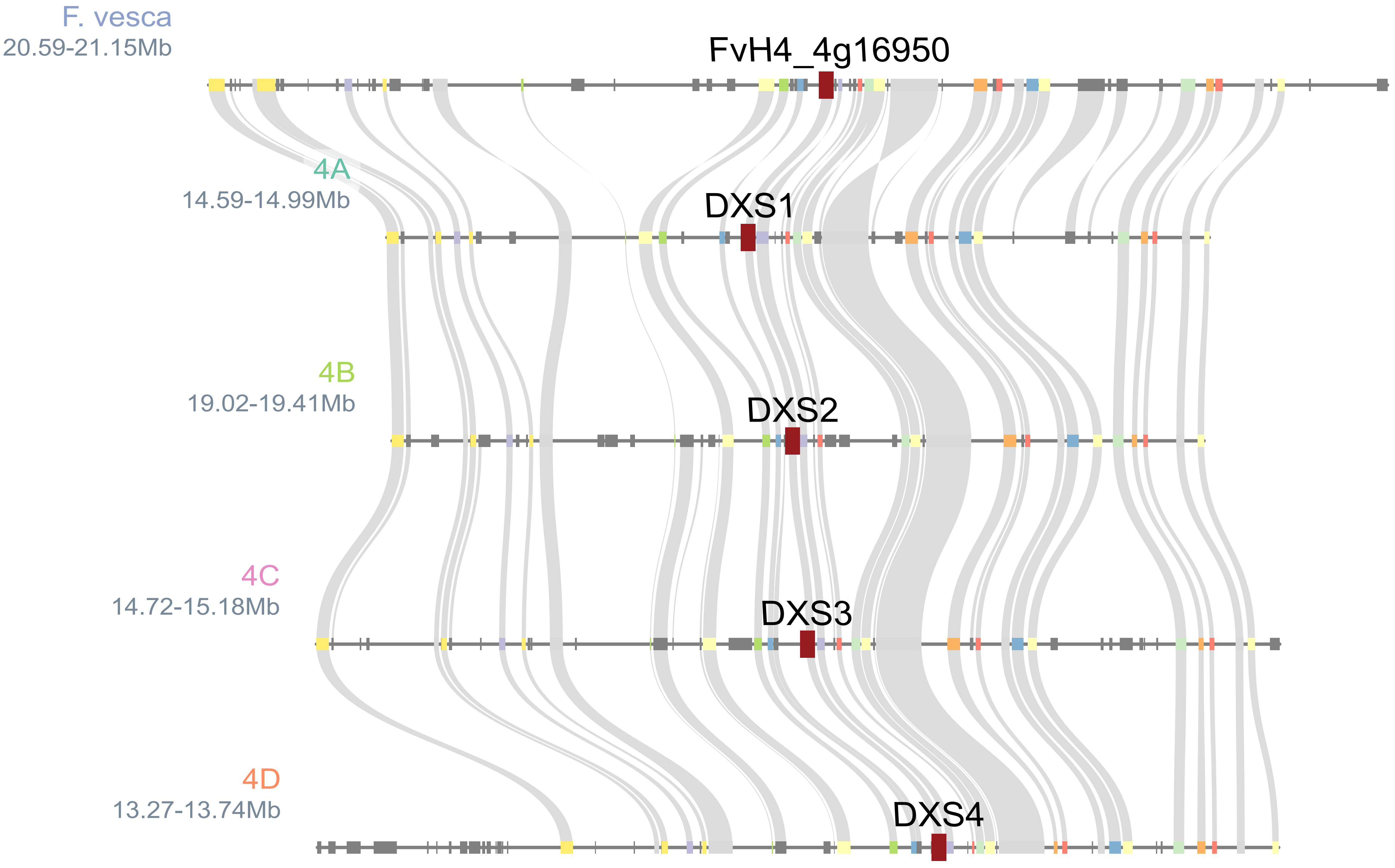


**B** DXR - 1-deoxy-D-xylulose 5-phosphate reductoisomerase

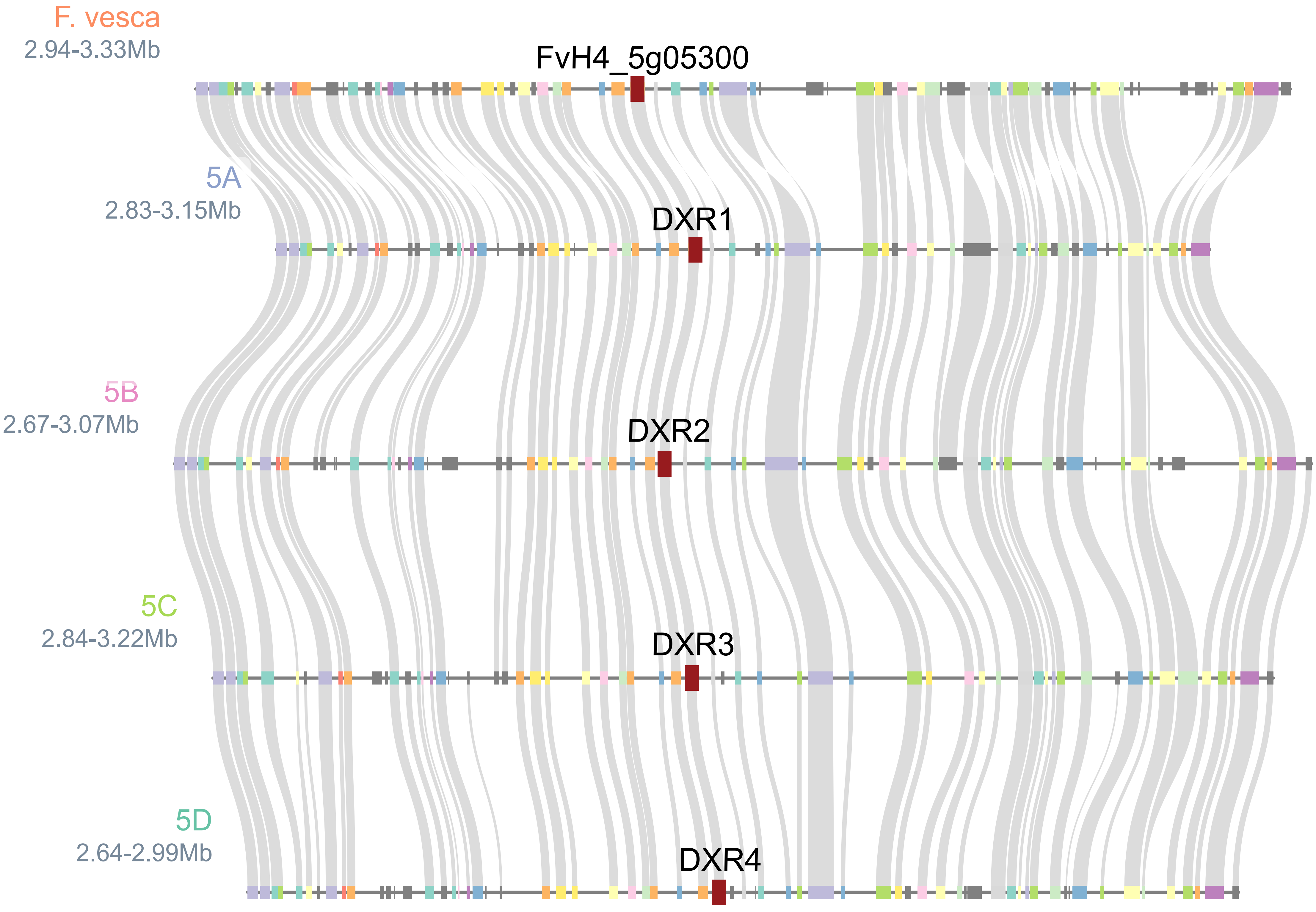

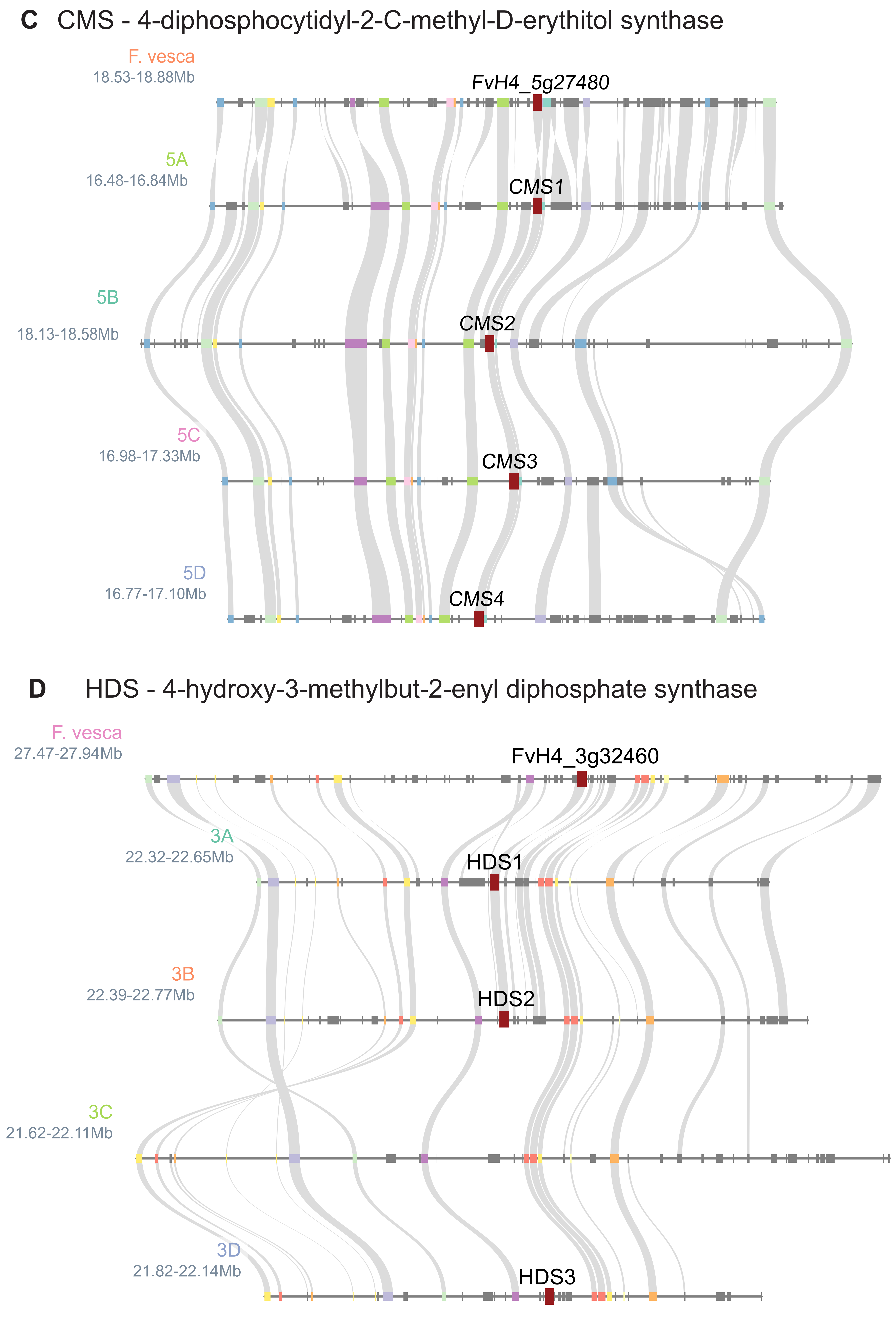
C CMS - 4-diphosphocytidyl-2-C-methyl-D-erythitol synthase
F. vesca
18.53-18.88Mb
FvH4_5g27480
5A
16.48-16.84Mb
CMS1
5B
18.13-18.58Mb
CMS2
5C
16.98-17.33Mb
CMS3
5D
16.77-17.10Mb
CMS4
D HDS - 4-hydroxy-3-methylbut-2-enyl diphosphate synthase
F. vesca
27.47-27.94Mb
FvH4_3g32460
3A
22.32-22.65Mb
HDS1
3B
22.39-22.77Mb
HDS2
3C
21.62-22.11Mb
3D
21.82-22.14Mb
HDS3

**E** HDR - 4-hydroxy-3-methylbut-2-enyl diphosphate reductase

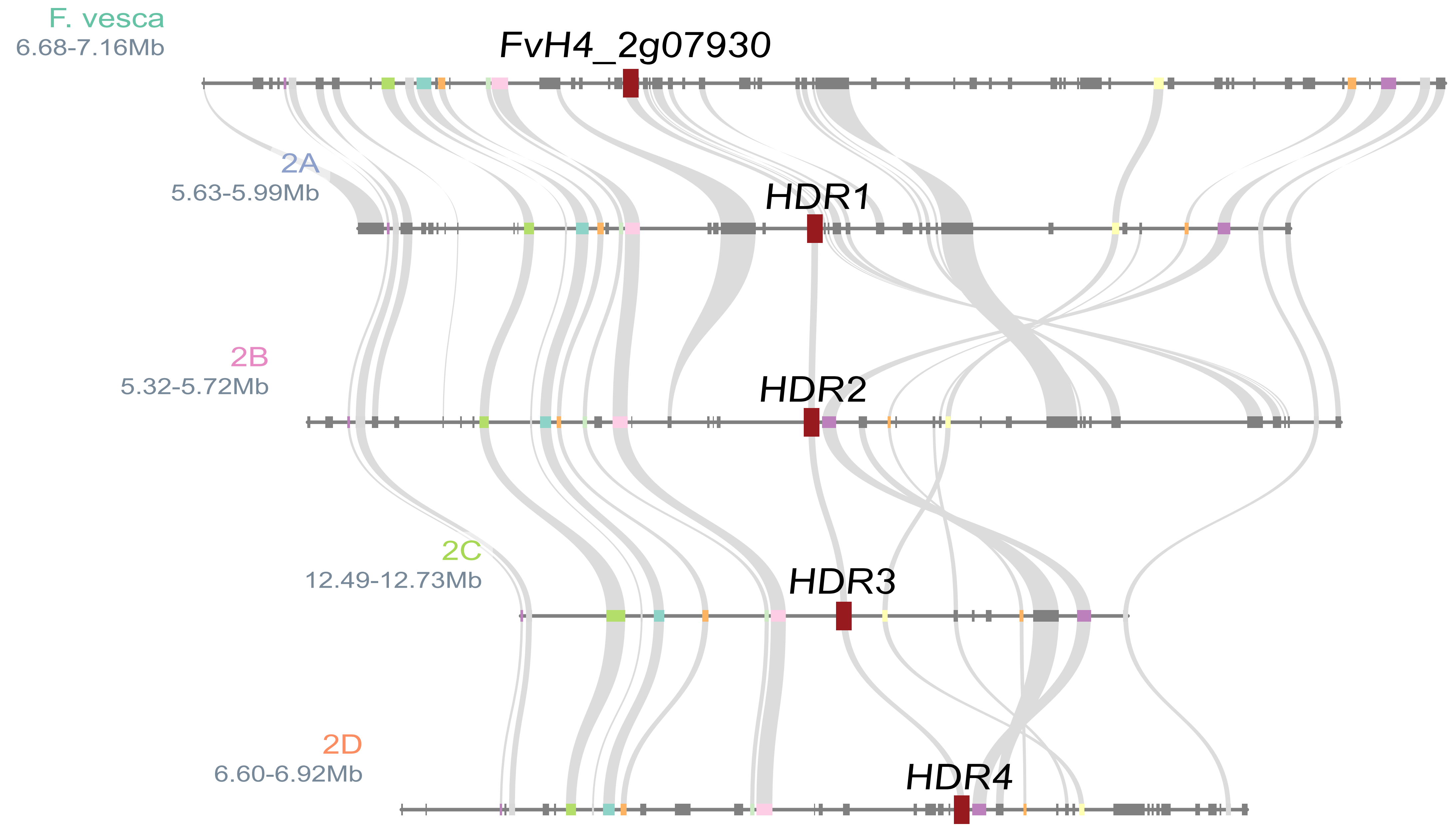

**Supplementary Fig. S4** Microsynteny plots of diploid *F. vesca* and FaRR1 Royal Royce of Mevalonate pathway genes. Genes of interest are highlighted in red.

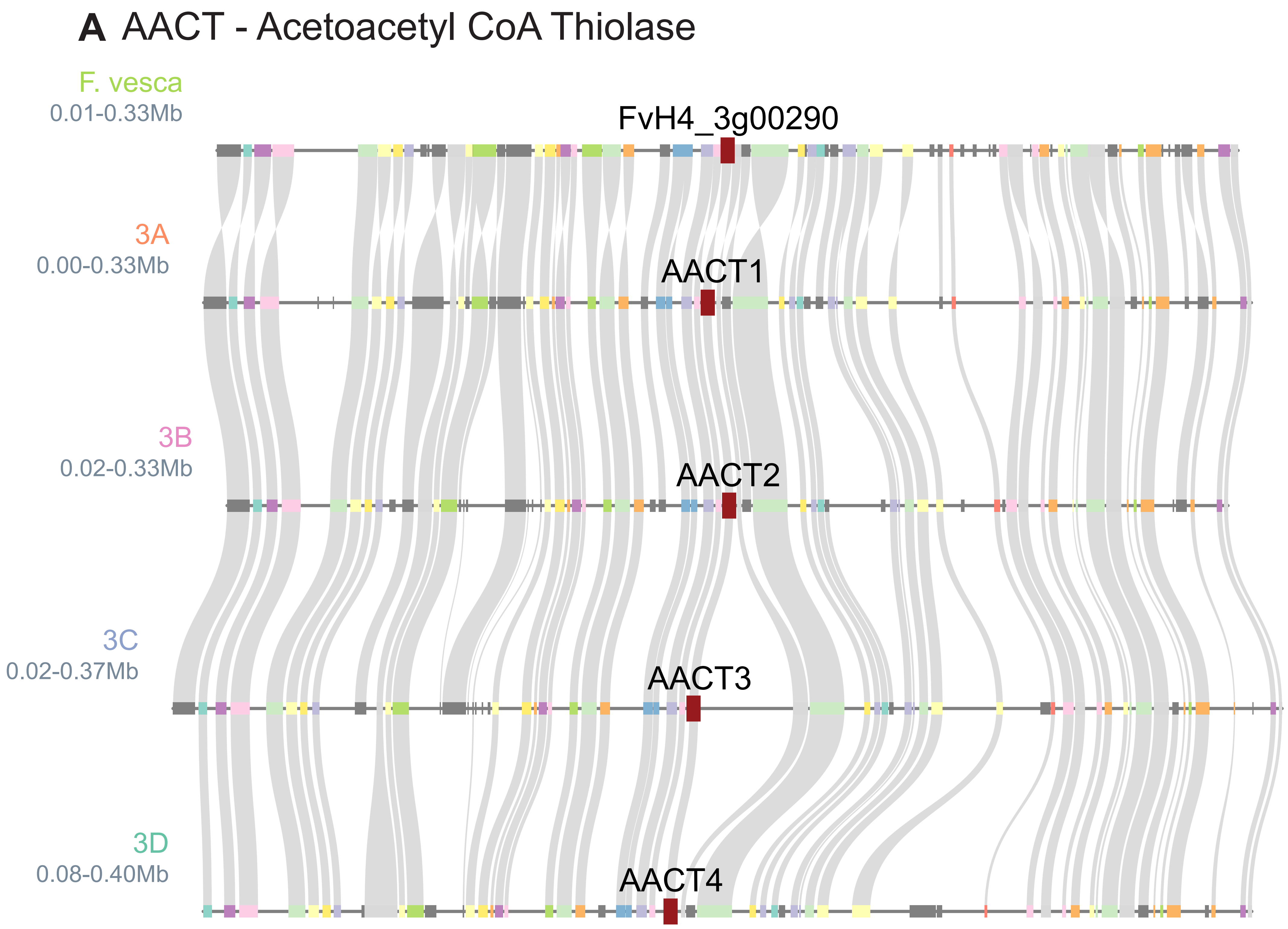


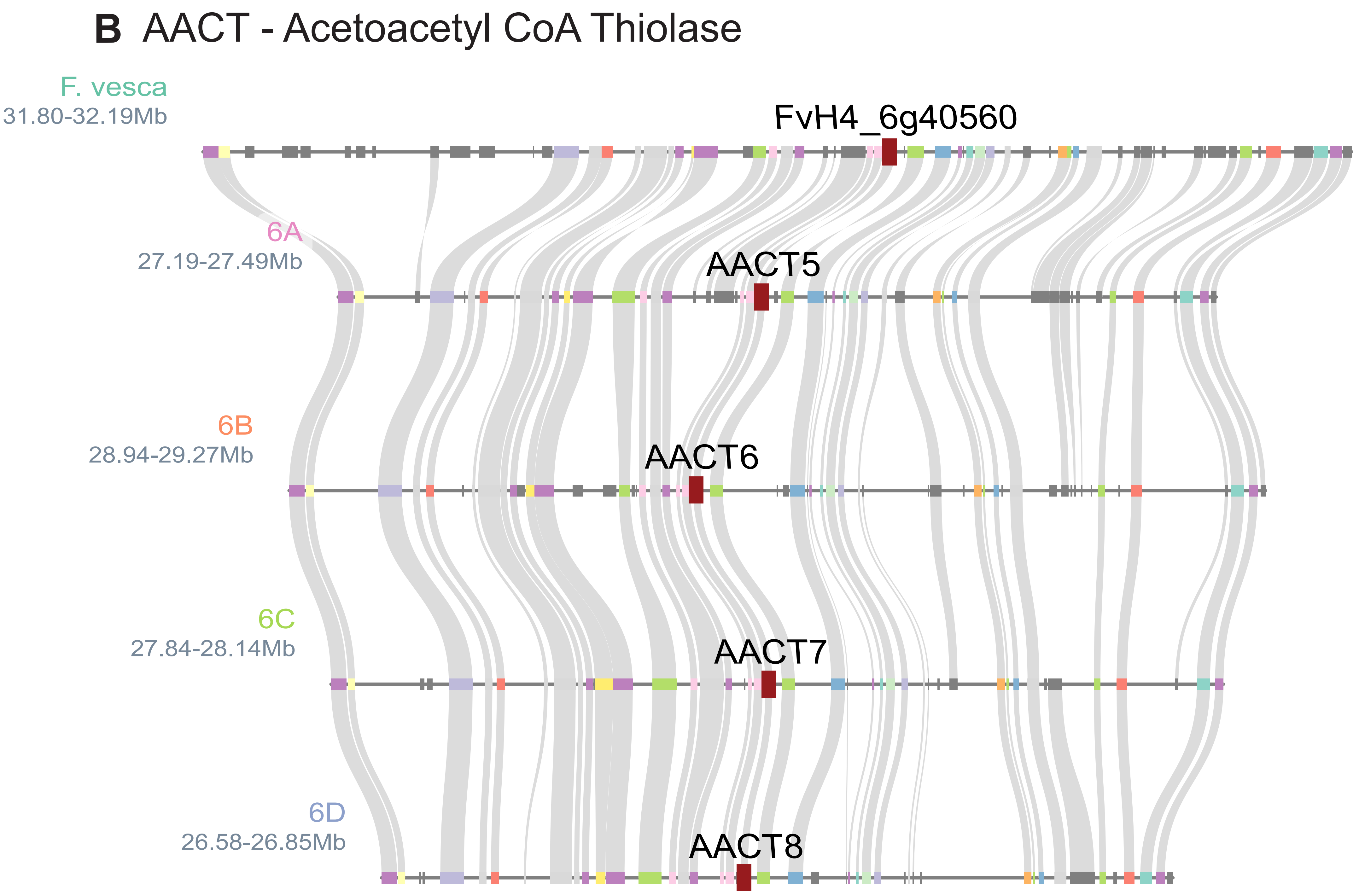

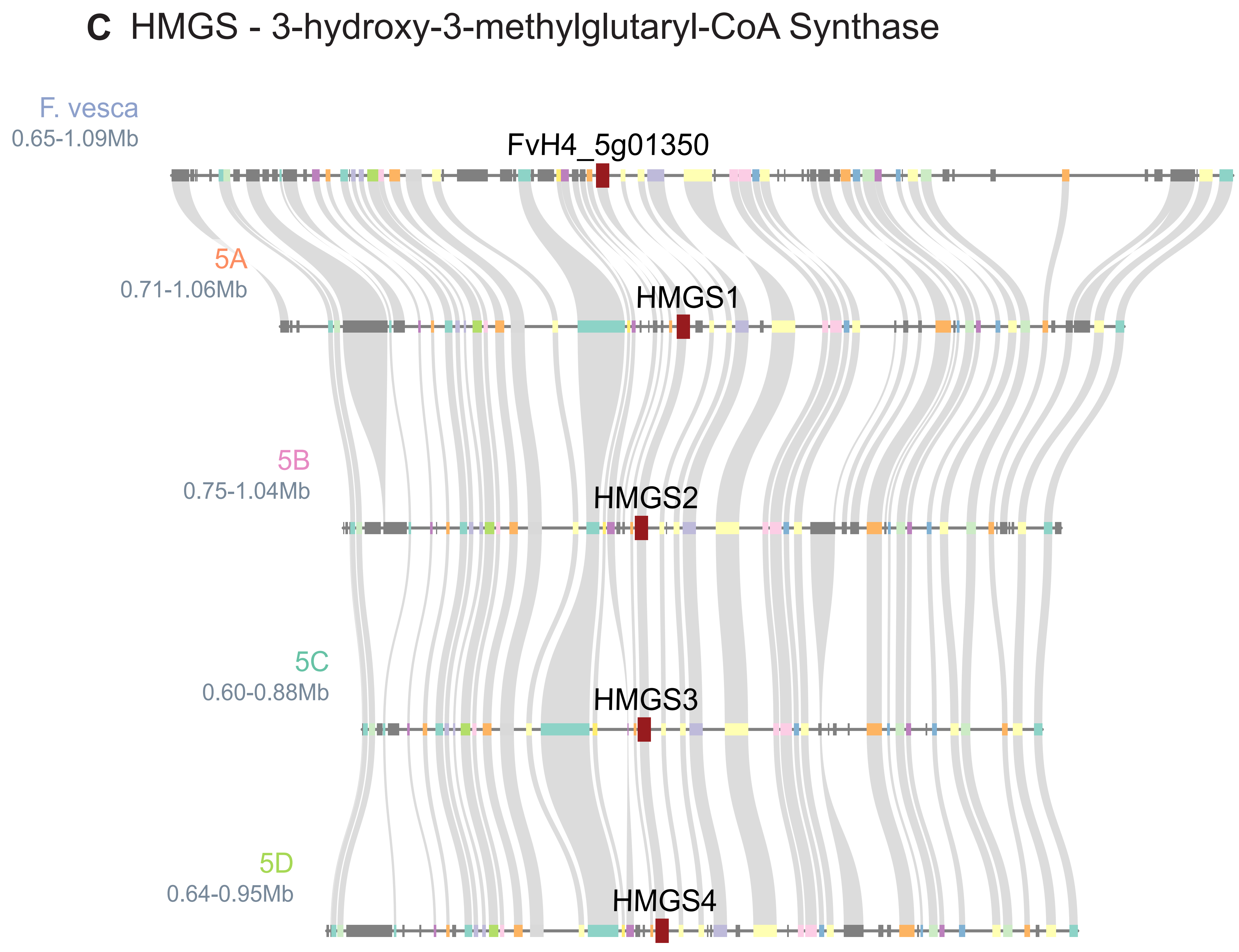
C HMGS - 3-hydroxy-3-methylglutaryl-CoA Synthase
F. vesca
0.65-1.09Mb
FvH4_5g01350
5A
0.71-1.06Mb
HMGS1
5B
0.75-1.04Mb
HMGS2
5C
0.60-0.88Mb
HMGS3
5D
0.64-0.95Mb
HMGS4

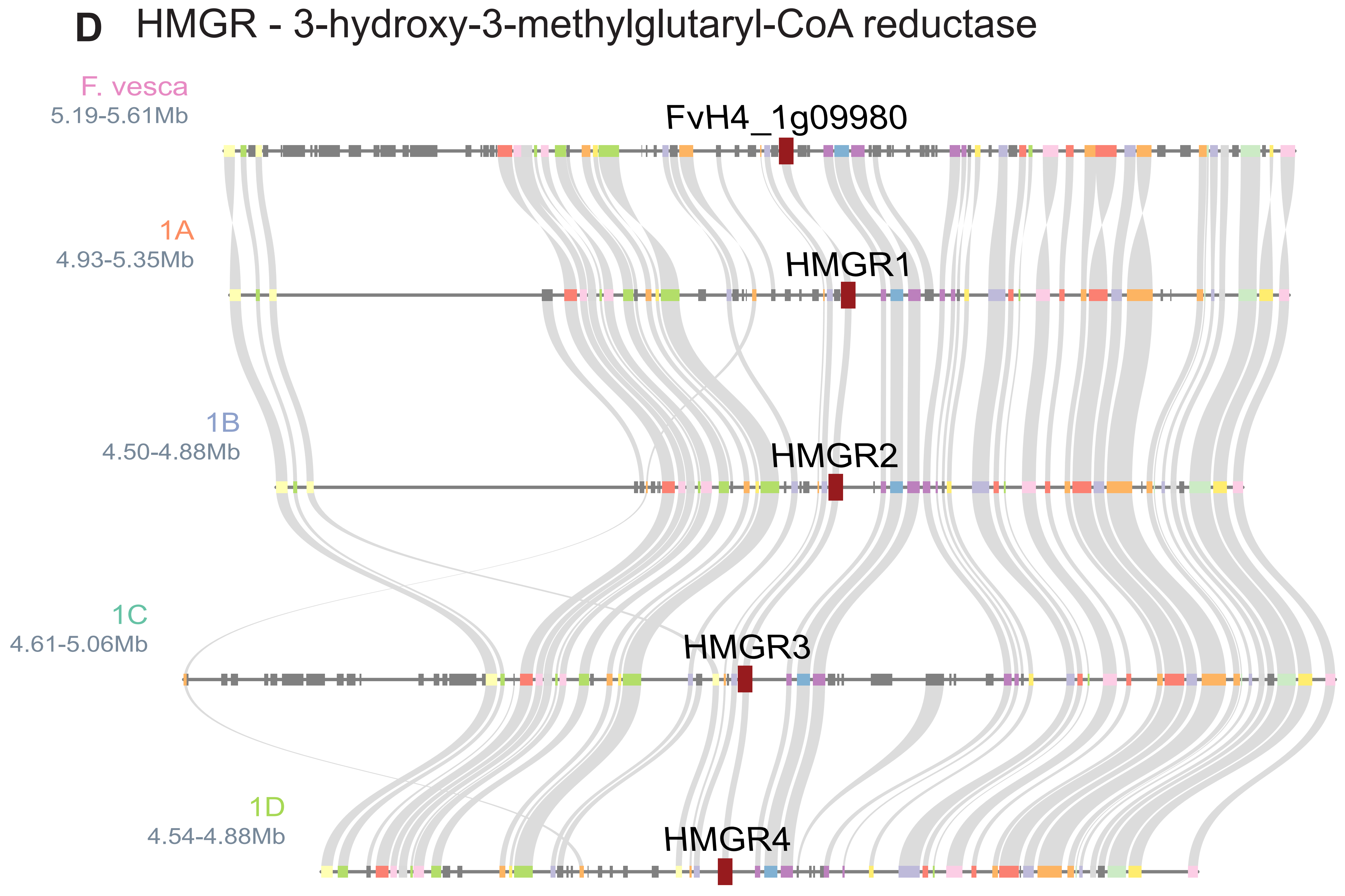
D HMGR - 3-hydroxy-3-methylglutaryl-CoA reductase
F. vesca
5.19-5.61Mb
FvH4_1g09980
1A
4.93-5.35Mb
HMGR1
1B
4.50-4.88Mb
HMGR2
1C
4.61-5.06Mb
HMGR3
1D
4.54-4.88Mb
HMGR4

**E** HMGR - 3-hydroxy-3-methylglutaryl-CoA reductase

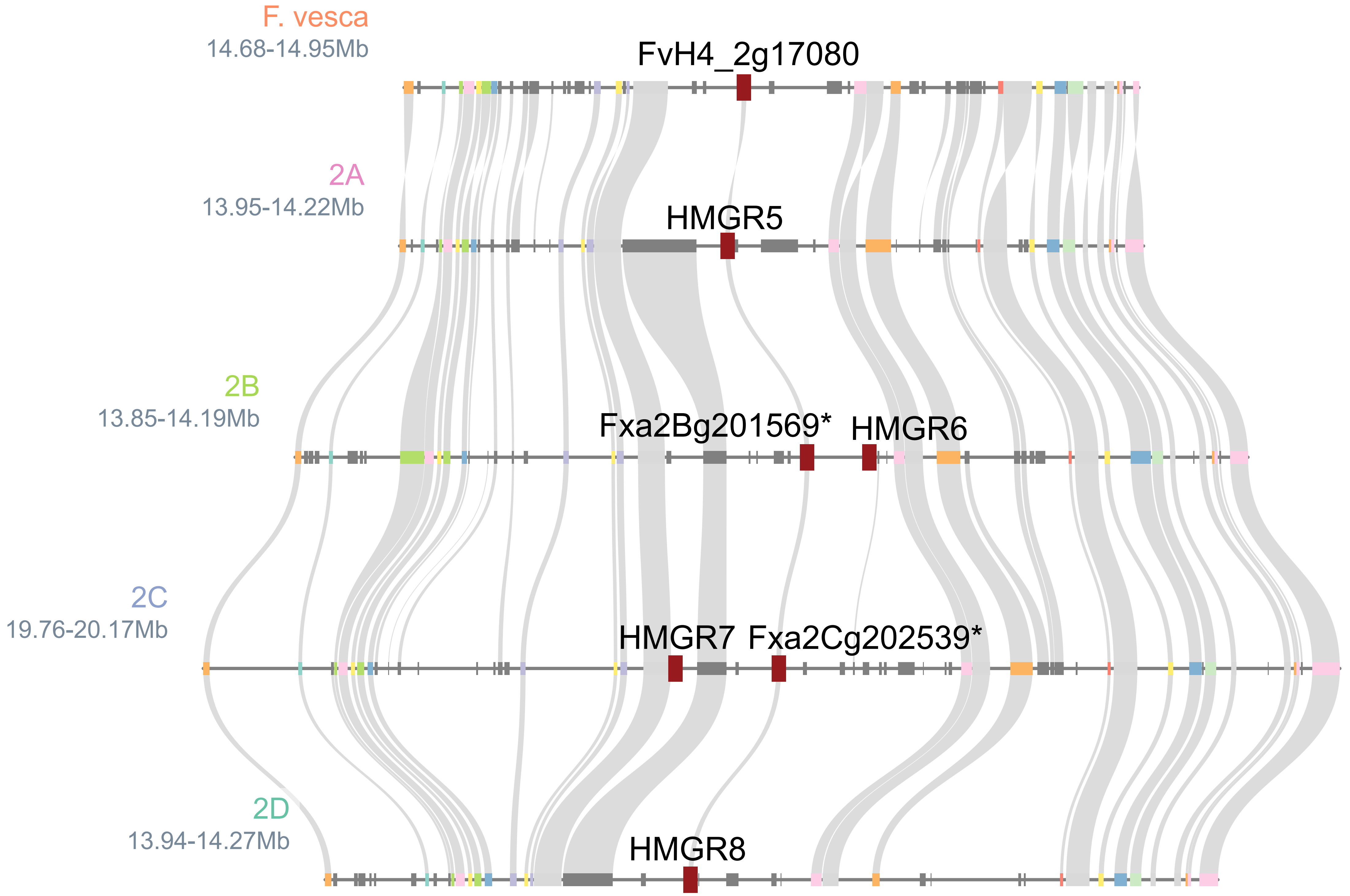

**Supplementary Fig. S5** Amino acid sequence alignments of the six putatively misassembled TPS sequences with the closest matches to either previously identified genes or those phylogenetically most closely related. Color coding: Black = missing regions in the alignment; brown = amino acid differences; DDxxD motif = yellow; DTAW motif = green; RRx8W motif = purple. Misassembled KSL4 (*Fxa3Dg201124.1*) (**A**) and NES (*Fxa3Cg100266.1*) (**B**) before sequence correction for gene synthesis are shown with other strawberry KSL and NES candidates, respectively. (**C, D, F**) Four TPS-a clade candidates *TPS9, TPS19, TPS24, TPS56*. (**E**) TPS-b clade *TPS43*.

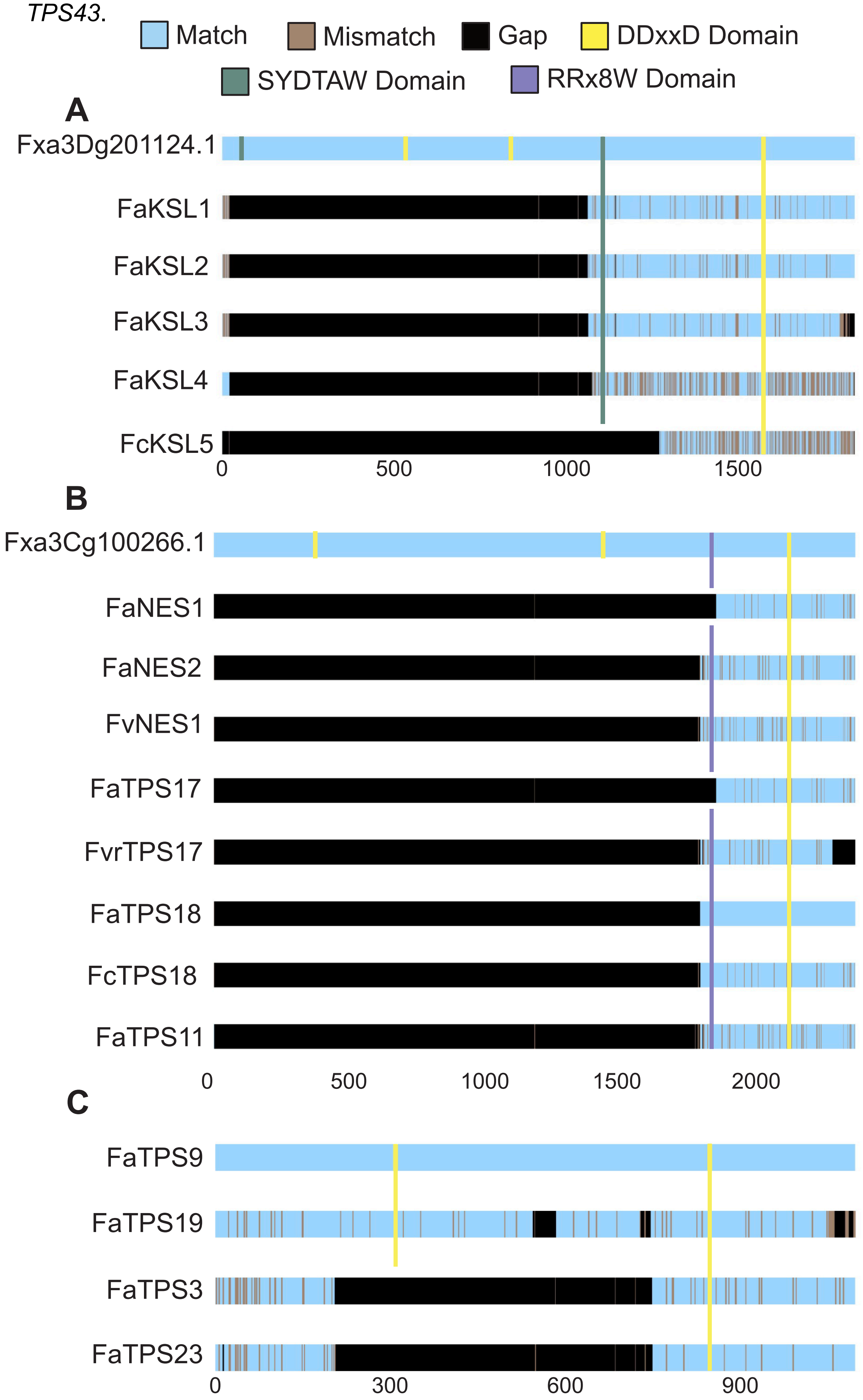

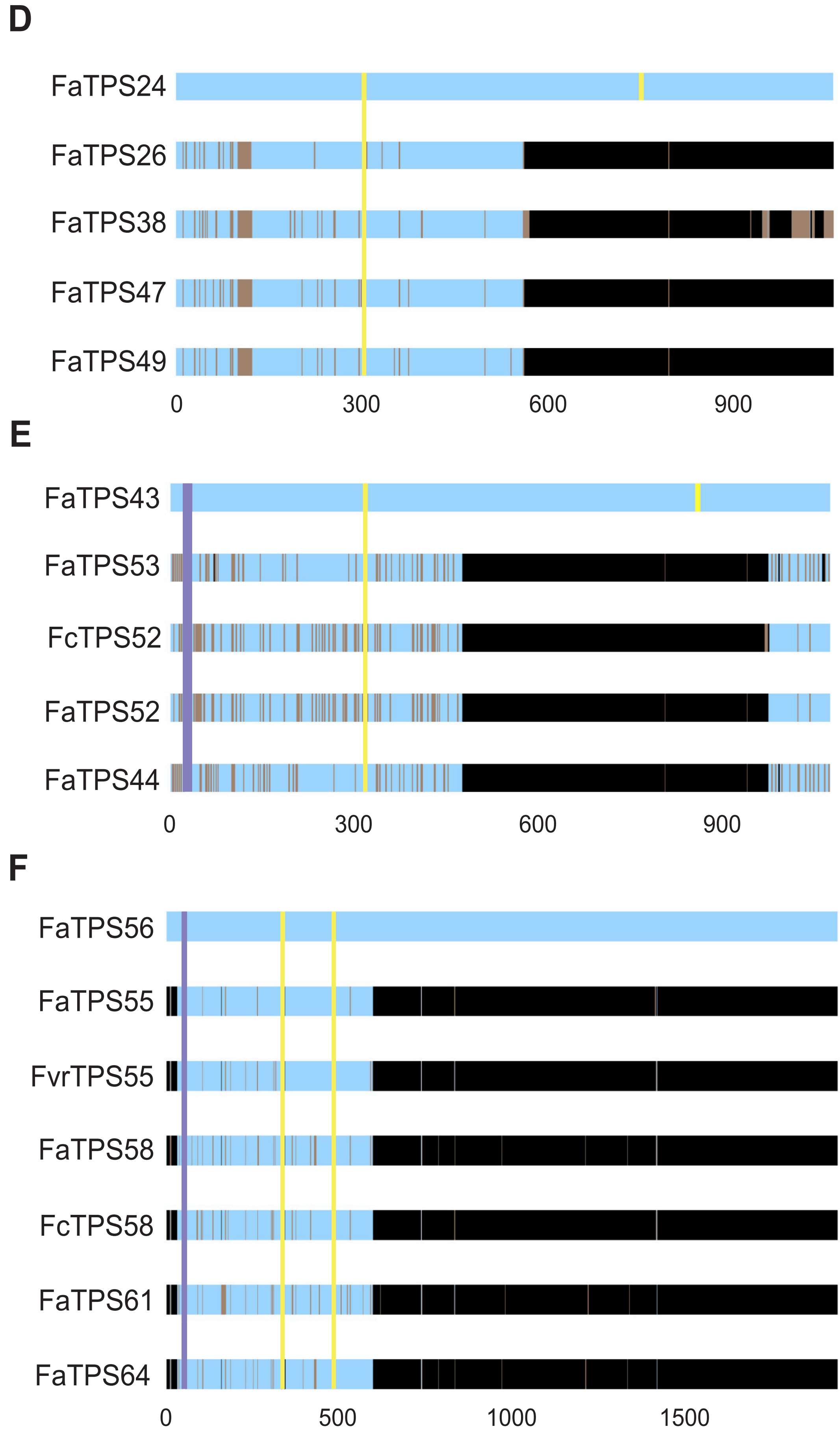
D
FaTPS24
FaTPS26
FaTPS38
FaTPS47
FaTPS49
0
300
600
900
E
FaTPS43
FaTPS53
FcTPS52
FaTPS52
FaTPS44
0
300
600
900
F
FaTPS56
FaTPS55
FvrTPS55
FaTPS58
FcTPS58
FaTPS61
FaTPS64
0
500
1000
1500

**Supplementary Fig. S6** Synteny plots of all 7 FaRR1 chromosomes with TPS genes identified in the diploid *F. vesca* genome. Gray lines show syntenic relationships between *TPS* genes. Syntenic pseudogenes are shown (gray). Chromosome 2C shown in green to depict whole chromosome inversion. Gene-dense region of chromosome 3 shown in detail; colored lines depict synteny matches across all 5 chromosomes.

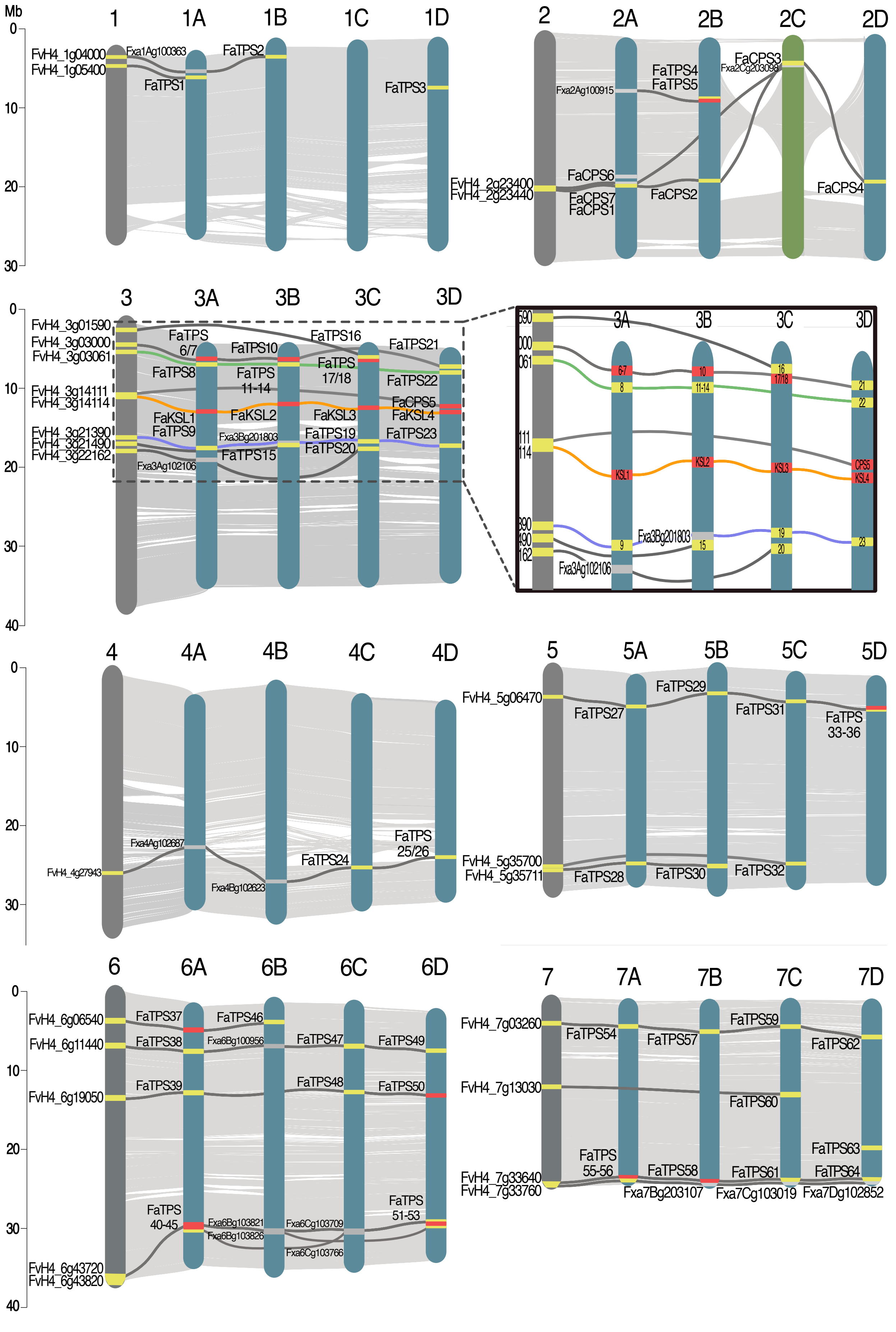

**Supplementary Fig. S7** Diterpene synthase gene synteny analysis. Microsynteny plots of diploid *F. vesca* and FaRR1 Royal Royce of diterpene synthases (**A**) Copalyl synthases (CPS) and (**B**) functionally tested Kaurene Synthases (KSL) tested in this study. Genes of interest are highlighted in red.

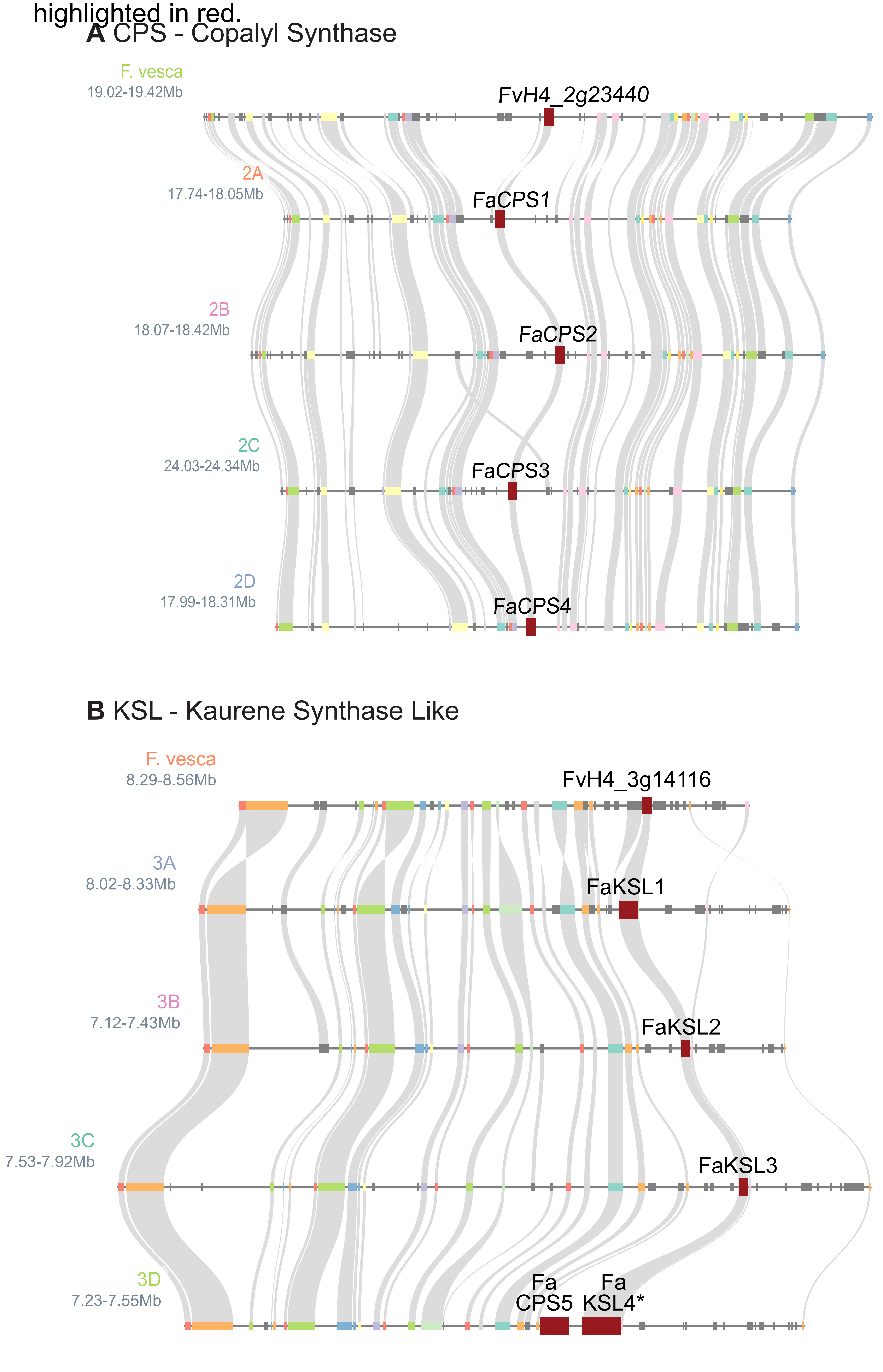

**Supplementary Fig. S8** Microsynteny plots of diploid *F. vesca* and FaRR1 Royal Royce of all functionally analyzed mono-, sesqui-terpene synthases. Genes of interest are highlighted in red.

**A** NES - Nerolidol Synthase Like

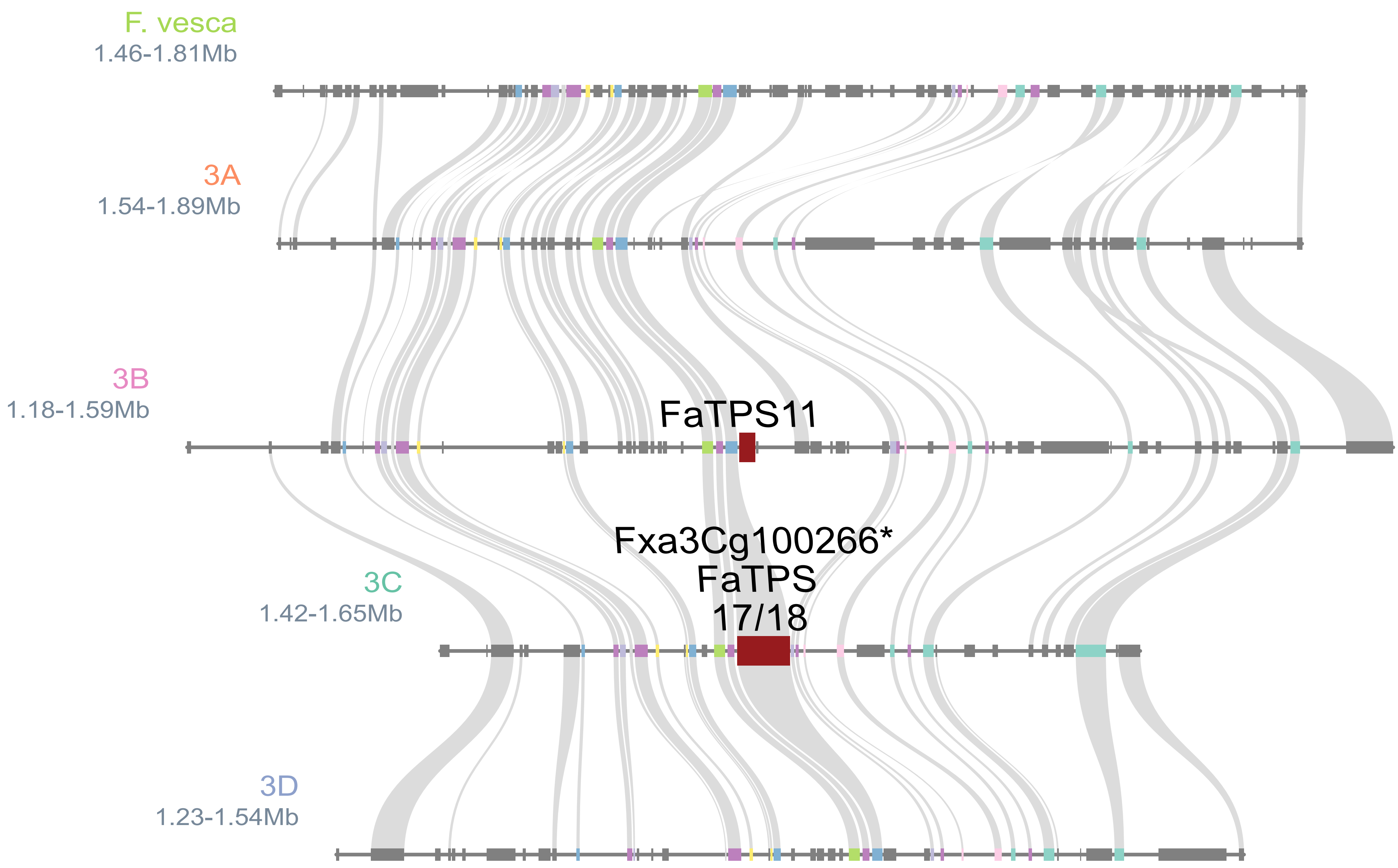


**B** FAR - Farnesene Synthase Like

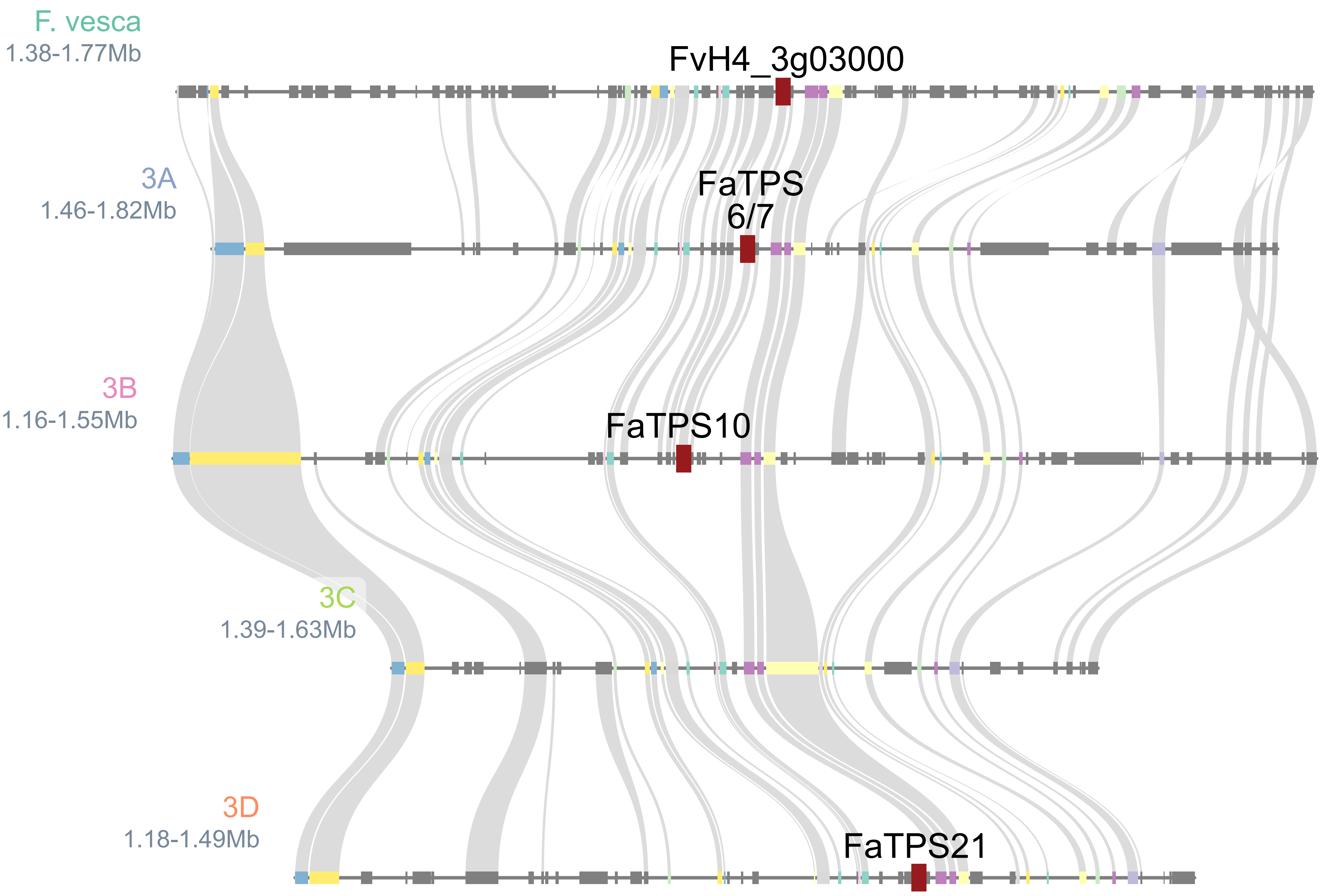

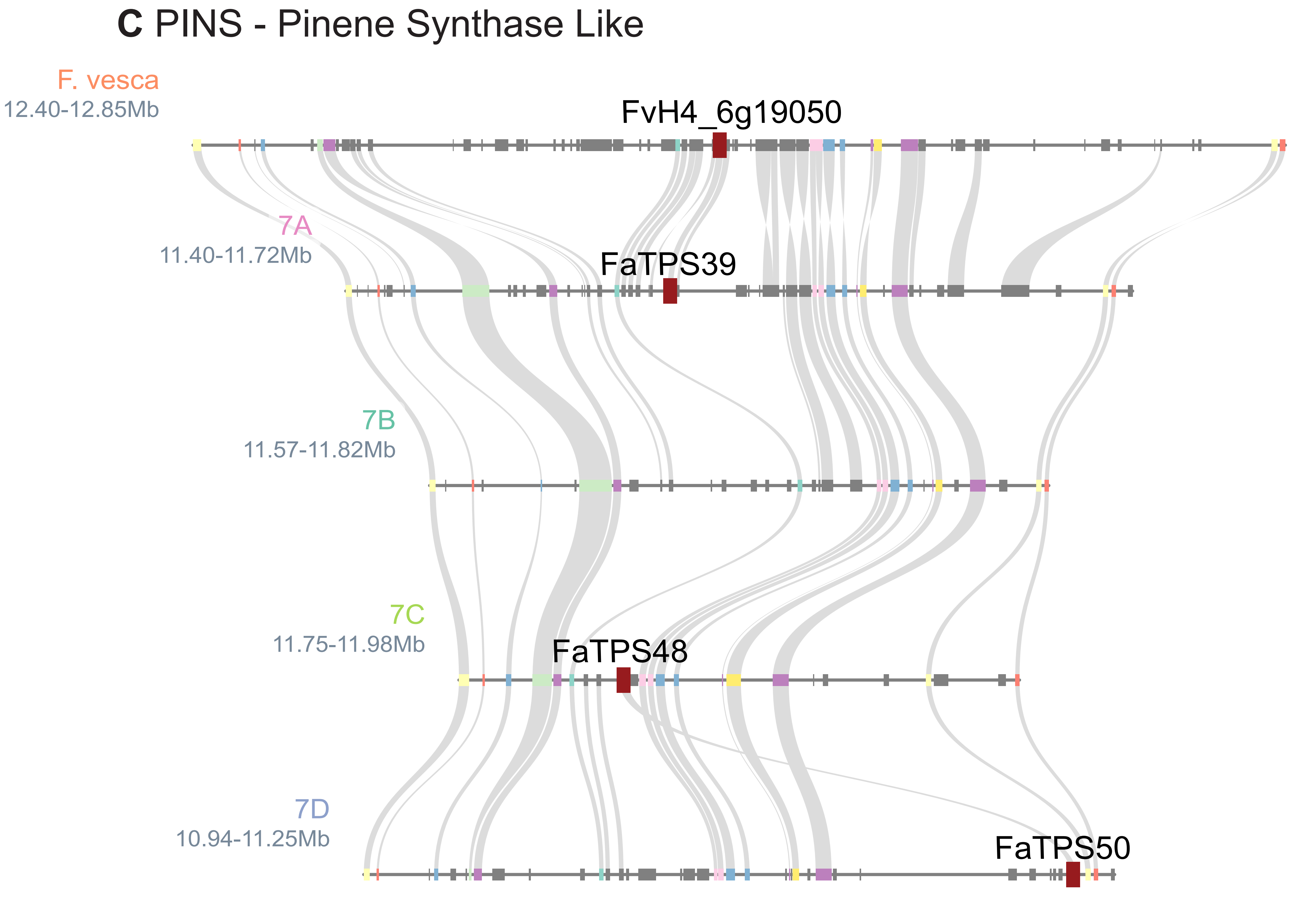
C PINS - Pinene Synthase Like
F. vesca
12.40-12.85Mb
FvH4_6g19050
7A
11.40-11.72Mb
FaTPS39
7B
11.57-11.82Mb
7C
11.75-11.98Mb
FaTPS48
7D
10.94-11.25Mb
FaTPS50

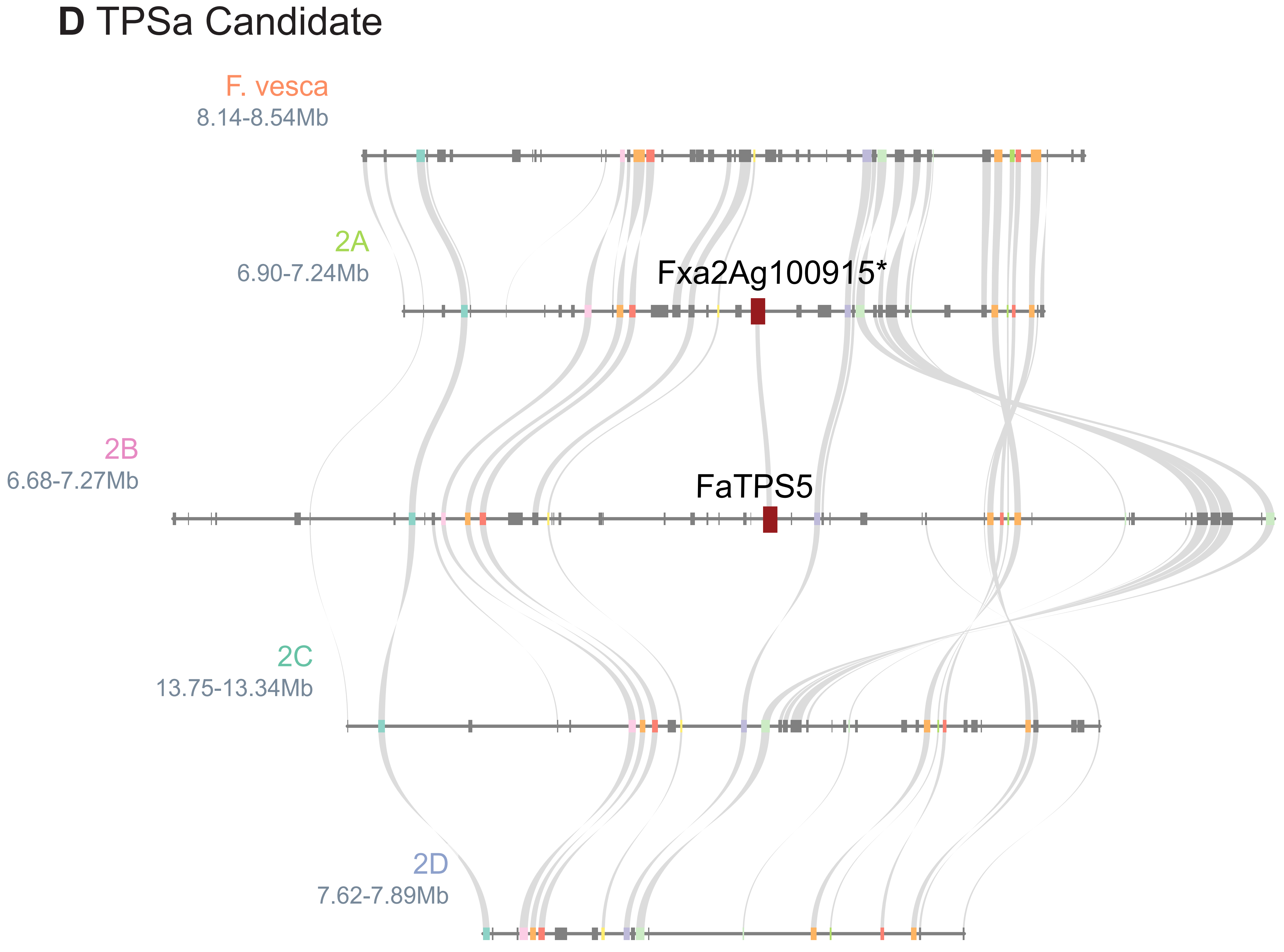
D TPSa Candidate
F. vesca
8.14-8.54Mb
2A
6.90-7.24Mb
Fxa2Ag100915*
2B
6.68-7.27Mb
FaTPS5
2C
13.75-13.34Mb
2D
7.62-7.89Mb

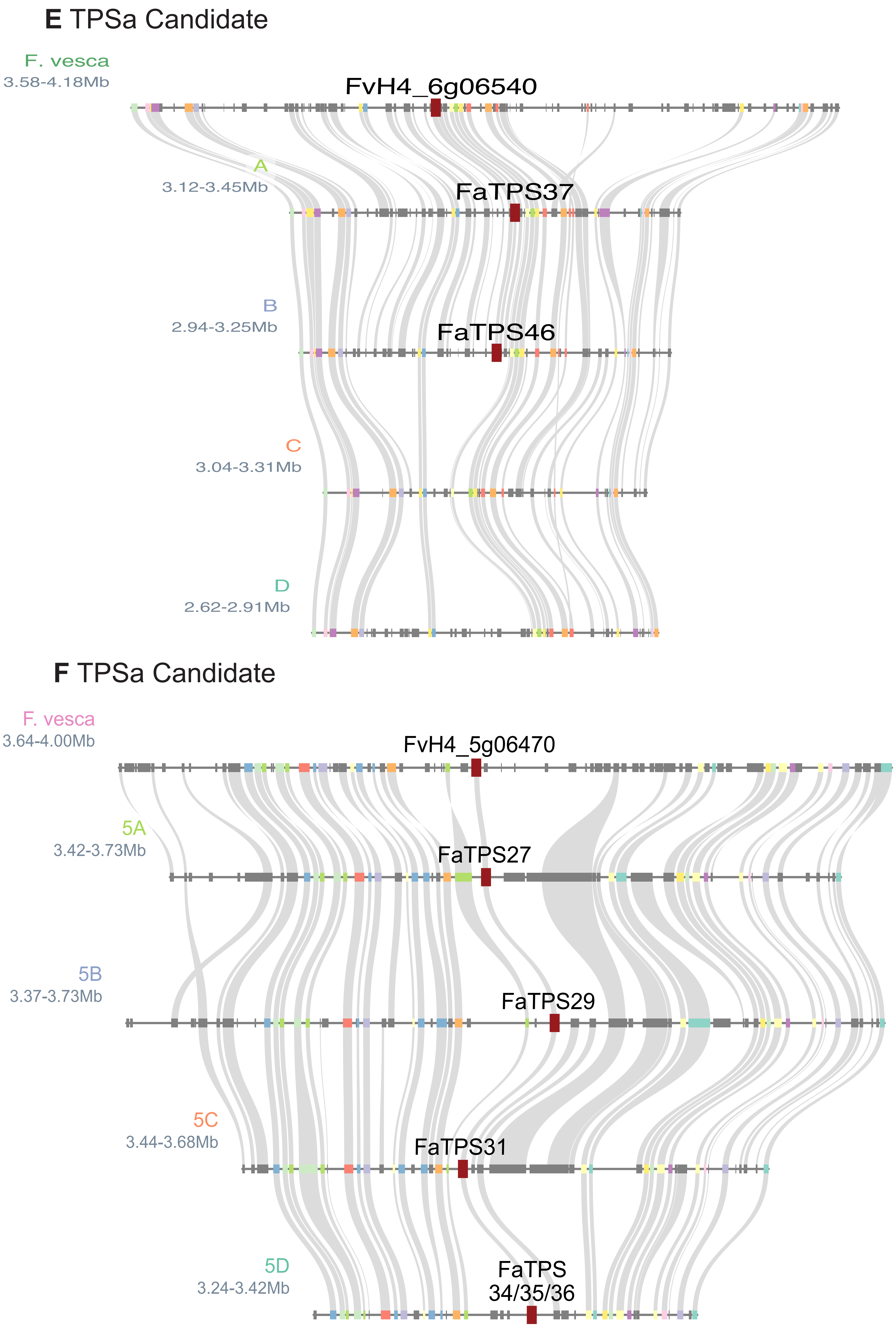
E TPSa Candidate
F. vesca
3.58-4.18Mb
FvH4_6g06540
A
3.12-3.45Mb
FaTPS37
B
2.94-3.25Mb
FaTPS46
C
3.04-3.31Mb
D
2.62-2.91Mb
F TPSa Candidate
F. vesca
3.64-4.00Mb
FvH4_5g06470
5A
3.42-3.73Mb
FaTPS27
5B
3.37-3.73Mb
FaTPS29
5C
3.44-3.68Mb
FaTPS31
5D
3.24-3.42Mb
FaTPS
34/35/36

**G** TPSa Candidate

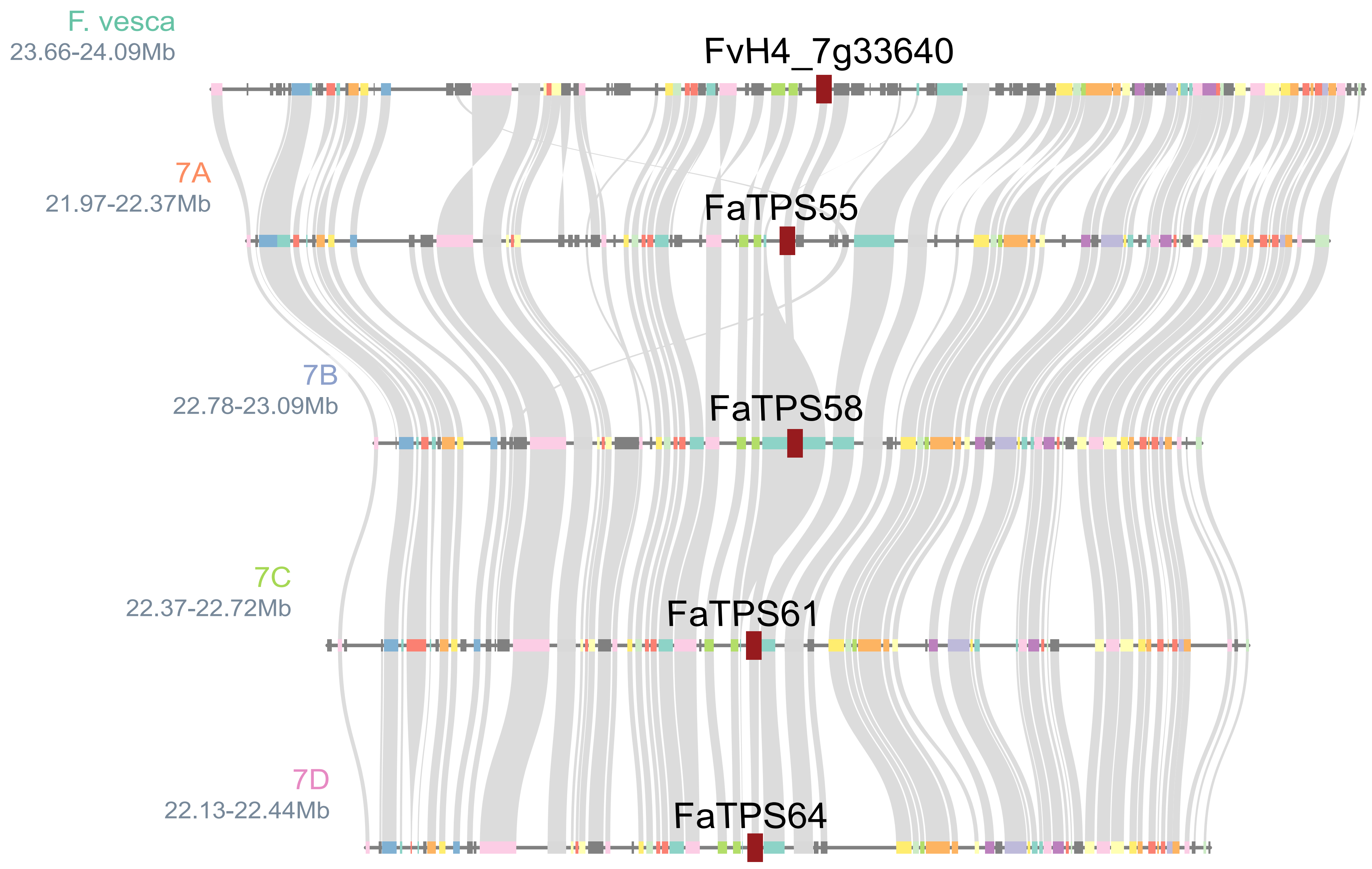


**H** TPSb Candidate

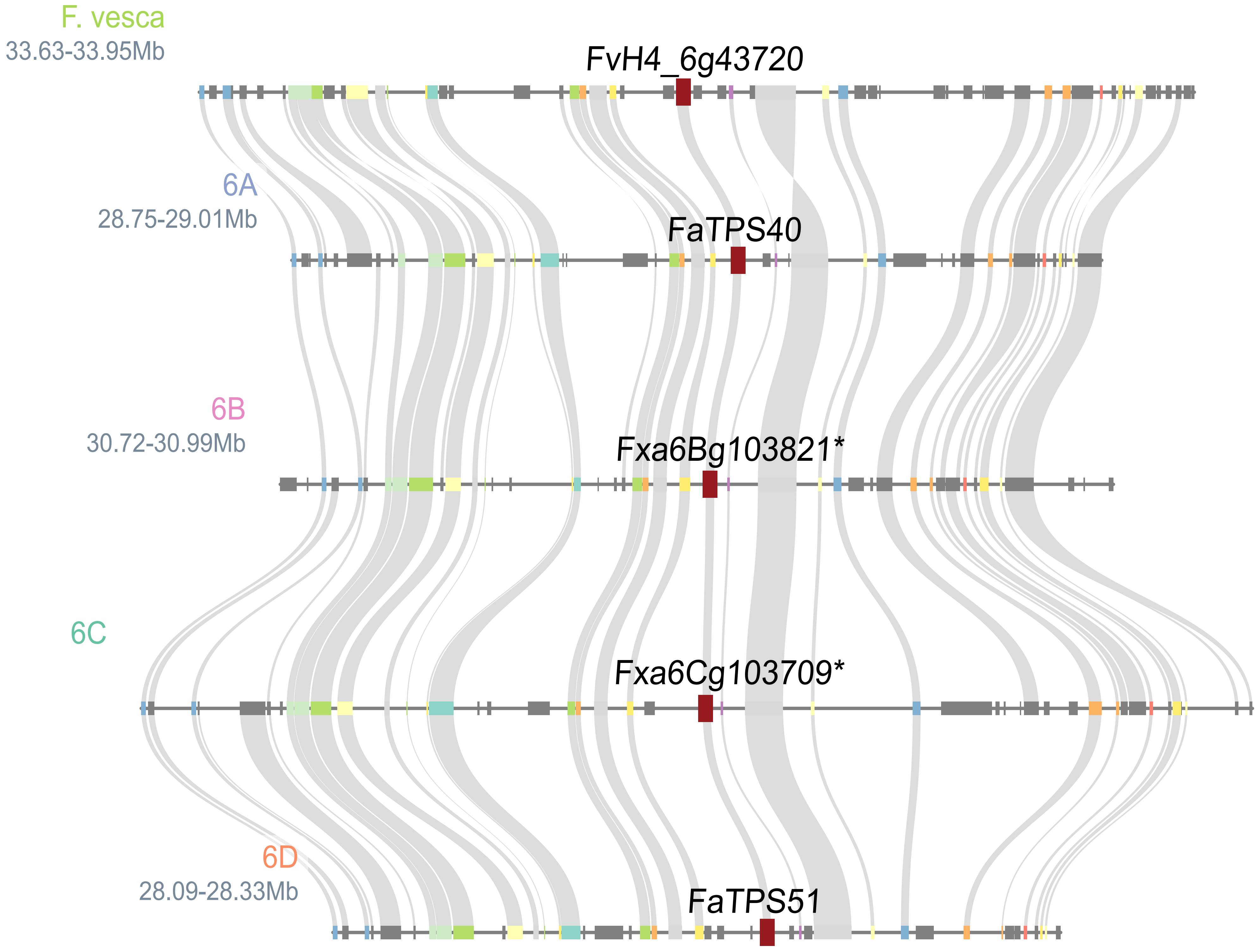

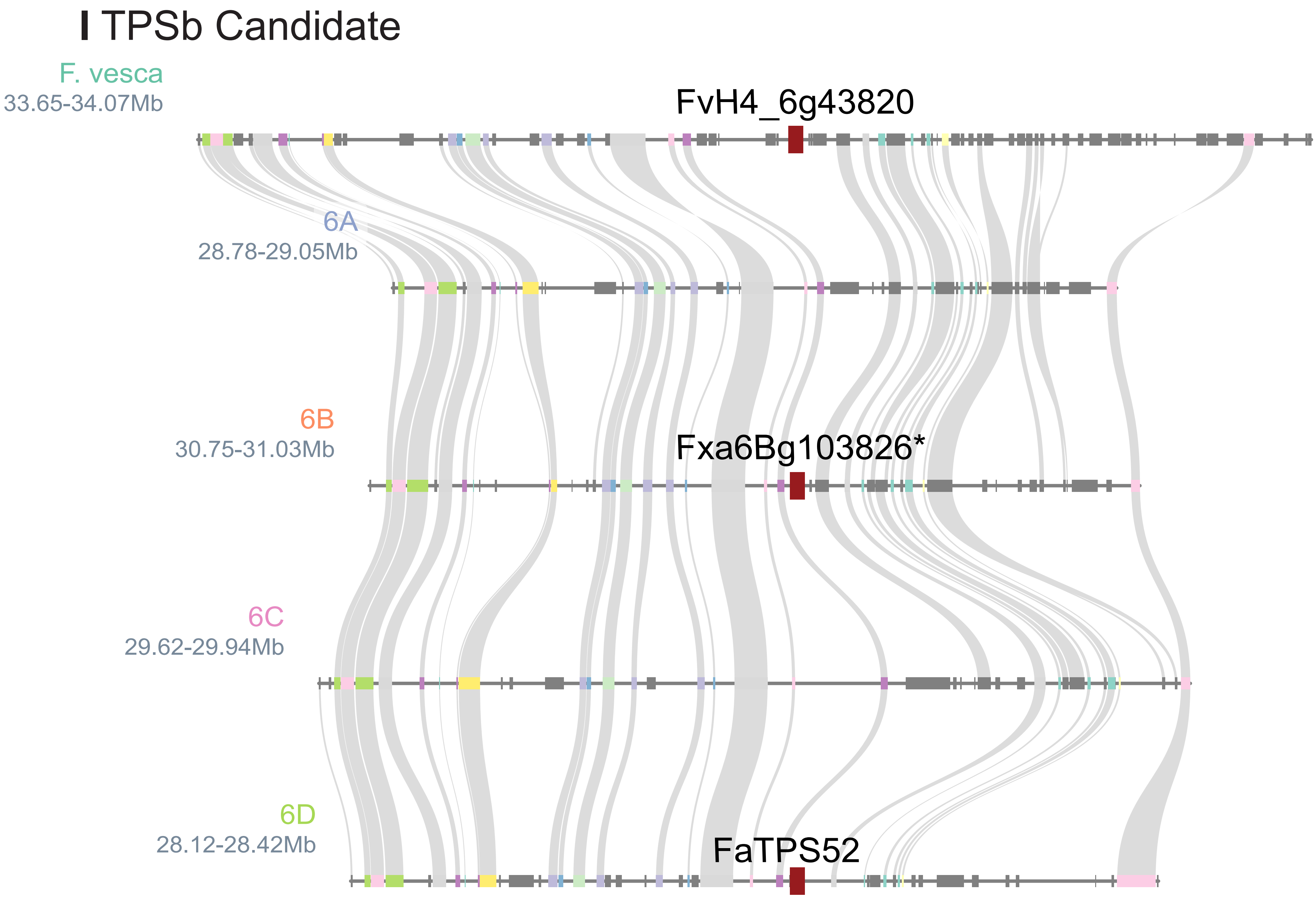
TPSb Candidate
F. vesca
33.65-34.07Mb
FvH4_6g43820
6A
28.78-29.05Mb
6B
30.75-31.03Mb
Fxa6Bg103826*
6C
29.62-29.94Mb
6D
28.12-28.42Mb
FaTPS52

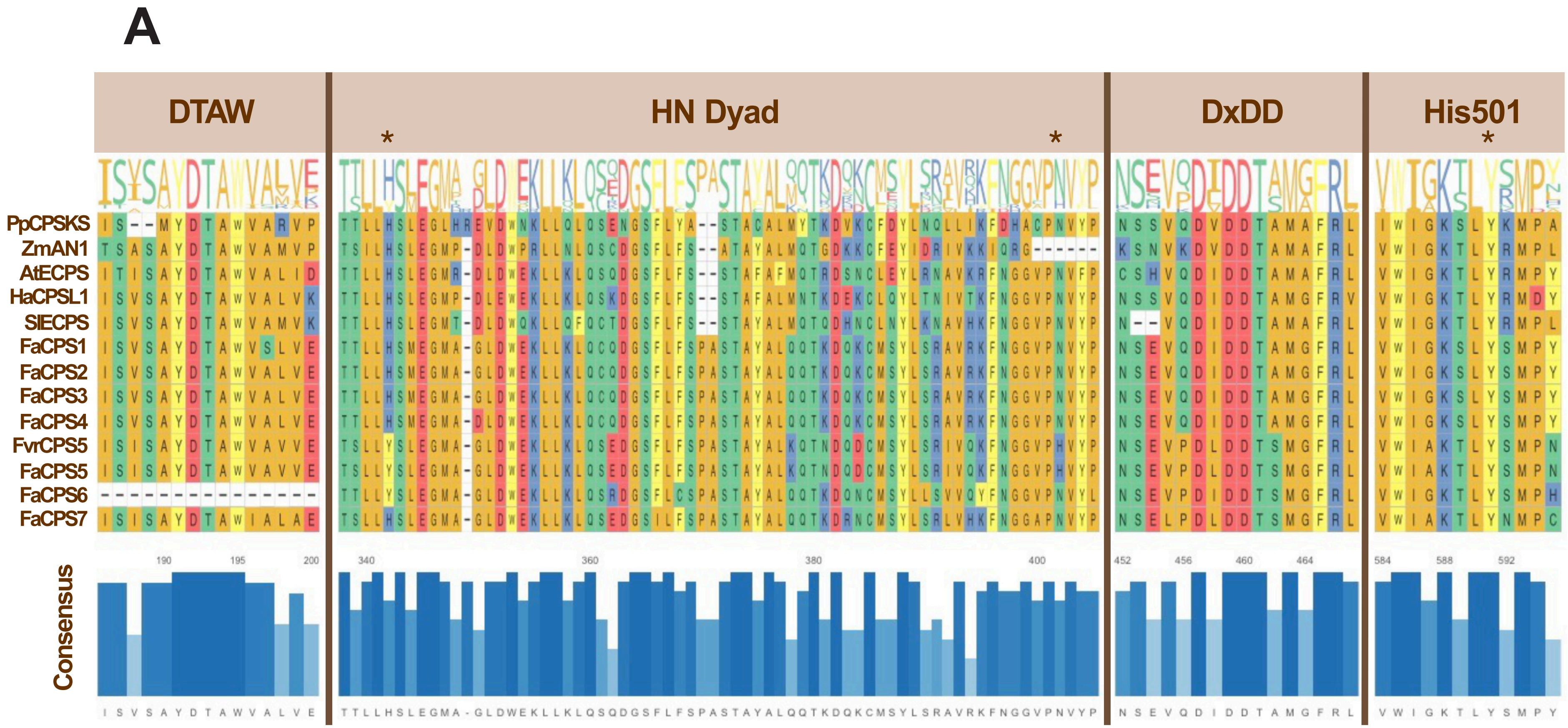


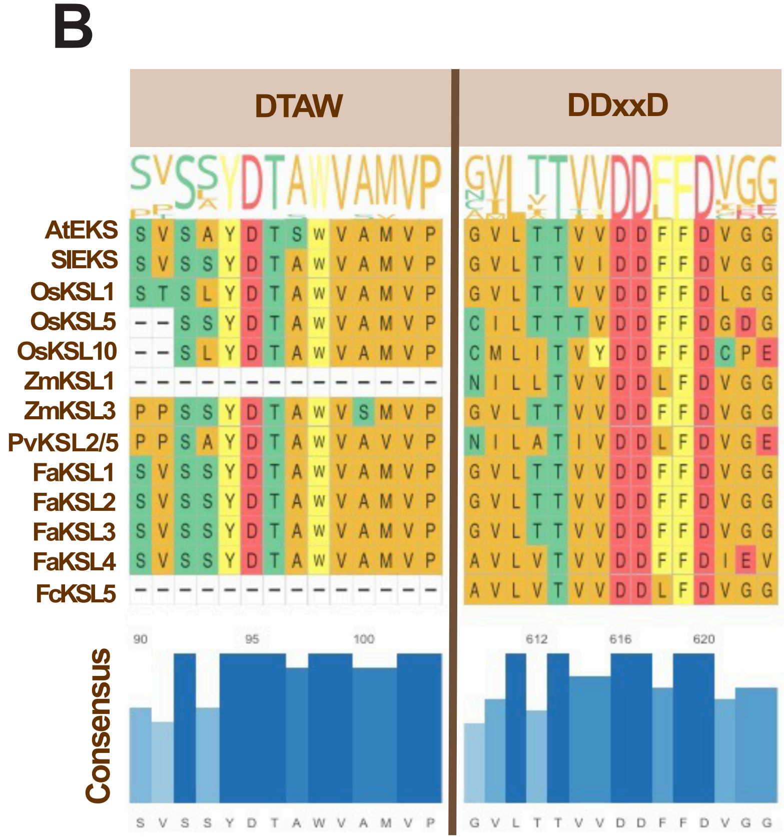


**Supplementary Fig. S9** Protein sequence alignment of identified diterpene synthase candidates. (**A**) FaCPS candidates and reference sequences from *Physcomitrella patens*, *Zea mays*, *Arabidopsis thaliana*, *Helianthus annuus*, *Oryza sativa* and *Solanum lycopersicum* showing the DTAW domain, H-N dyad and DDxD motif. Additional OsCPS4 His501 residue representing *syn*-CPS activity is shown. (**B**) FaKSL candidates and reference sequences shown with DTAW domain and catalytic DxxDD motif.

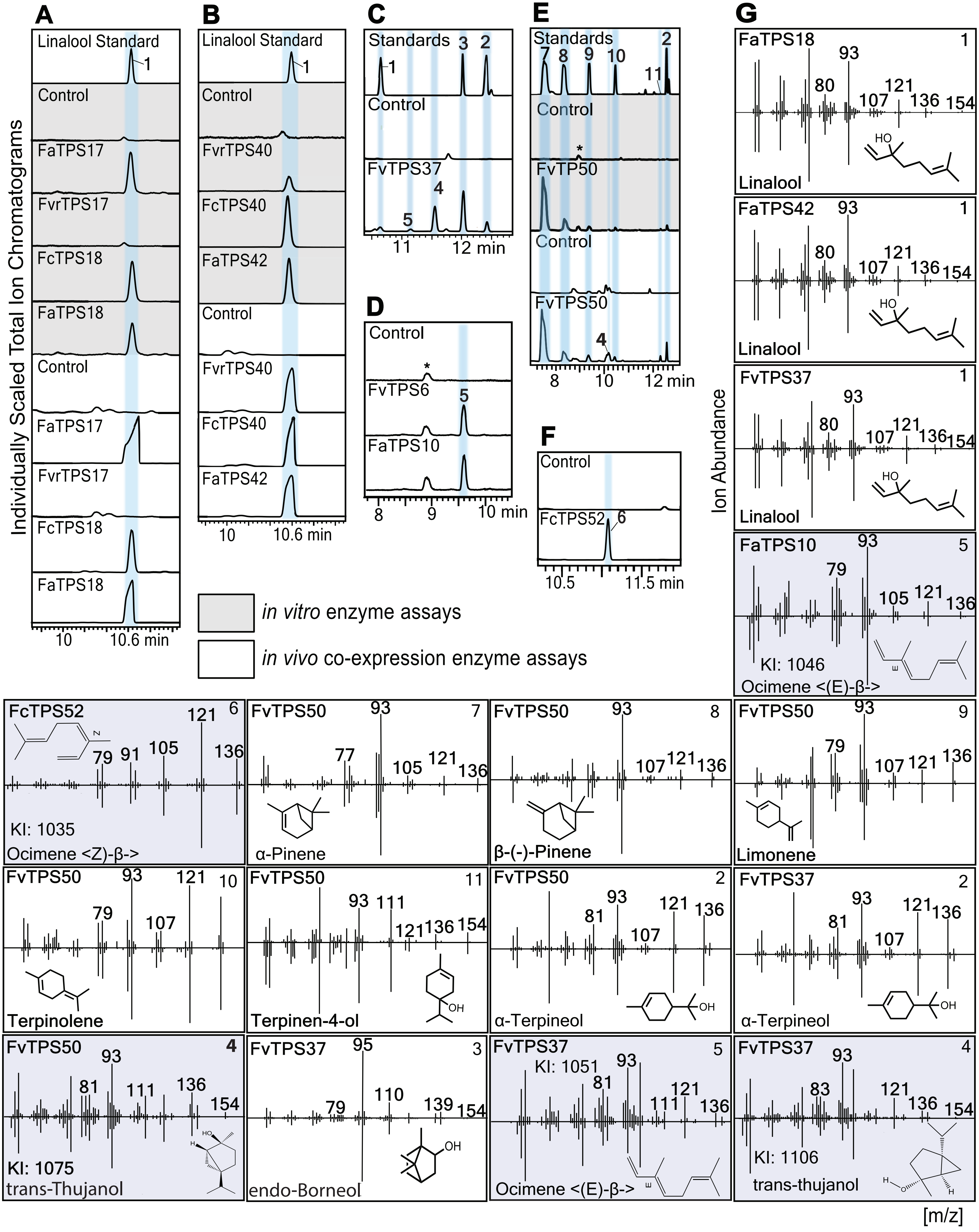
A
Linalool Standard
1
Control
FaTPS17
FvrTPS17
FcTPS18
FaTPS18
Control
FaTPS17
FvrTPS17
FcTPS18
FaTPS18
10
10.6 min
Individually Scaled Total Ion Chromatograms
B
Linalool Standard
1
Control
FvrTPS40
FcTPS40
FaTPS42
Control
FvrTPS40
FcTPS40
FaTPS42
10
10.6 min
C
Standards
1
3
2
Control
FvTPS37
5
4
11
12 min
D
Control
*
FvTPS6
5
FaTPS10
8
9
10 min
E
Standards
7
8
9
10
11
2
Control
*
FvTP50
Control
FvTPS50
4
8
10
12 min
F
Control
FcTPS52
6
10.5
11.5 min
in vitro enzyme assays
in vivo co-expression enzyme assays
G
Ion Abundance
FaTPS18
1
93
80
121
107
136
154
Linalool
FaTPS42
1
93
80
121
107
136
154
Linalool
FvTPS37
1
93
80
121
107
136
154
Linalool
FaTPS10
5
93
79
105
121
136
KI: 1046
Ocimene <(E)-β->
FcTPS52
6
121
79
91
105
136
KI: 1035
Ocimene <Z)-β->
FvTPS50
7
93
77
105
121
136
α-Pinene
FvTPS50
8
93
107
121
136
β-(-)-Pinene
FvTPS50
9
93
79
107
121
136
Limonene
FvTPS50
93
121
10
79
107
Terpinolene
FvTPS50
11
93
111
121
136
154
Terpinen-4-ol
FvTPS50
2
93
81
121
136
107
α-Terpineol
FvTPS37
2
93
81
121
136
107
α-Terpineol
FvTPS50
4
93
81
111
136
154
KI: 1075
trans-Thujanol
FvTPS37
95
3
79
110
139
154
endo-Borneol
FvTPS37
5
KI: 1051
93
81
121
111
136
Ocimene <(E)-β->
FvTPS37
4
93
83
121
136
154
KI: 1106
trans-thujanol
[m/z]

**Supplementary Fig. S10** Functional characterization of terpene synthases (TPSs). (**A-F**) GC-MS traces of products resulting from either in vitro enzyme assays of individual recombinant TPSs with geranyl diphosphate (GPP) as a substrate (gray) or co-expression assays ofindividual TPSs and a GPP synthase in *E. coli* (white). (**G**) Mass spectra of enzyme products identified by comparison to authentic standards (white) or mass spectral databases (NIST,v17.1; purple). FaTPS17, TPS18, and TPS42 taken from *F. x ananassa* (Fa) cultivar 'RoyalRoyce'; FvrTPS17 *F. virginiana* (Fvr) accession 'NC_96-35-2'; FcTPS18 *F. chiloensis* (Fc)ecotype 'Ambato'; FvrTPS40 accession 'Harris Springs'; FcTPS40 and FcTPS52 ecotype 'Islede Lemuy'; FvTPS6 and FvTPS50 diploid accession 'UC06'; FaTPS10 cultivar 'EarliMiss'.

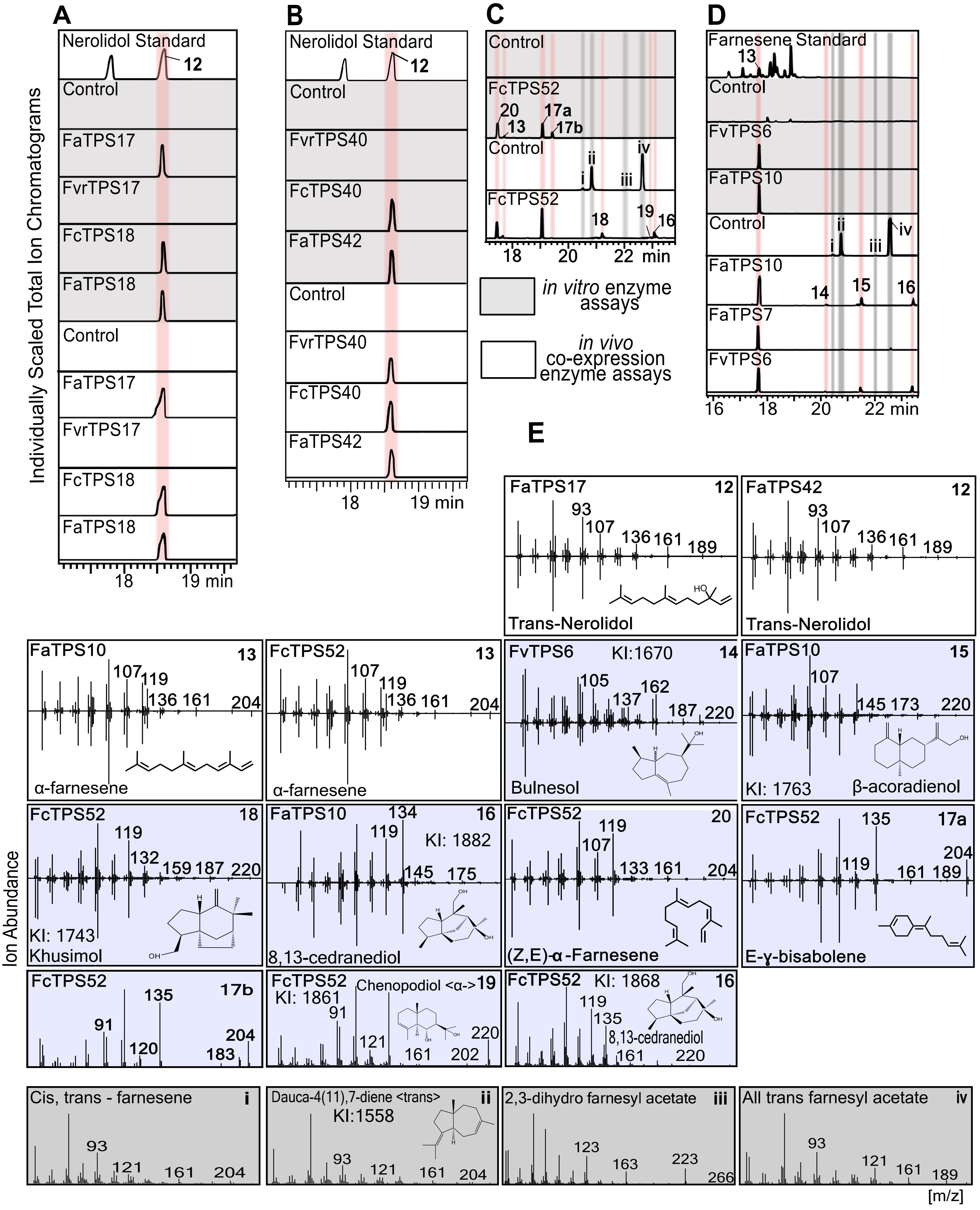
A
B
C
D
E
Nerolidol Standard
12
Control
FaTPS17
FvrTPS17
FcTPS18
FaTPS18
FvrTPS40
FcTPS40
FaTPS42
FcTPS52
20
13
17a
17b
i
ii
iii
iv
18
19
16
Farnesene Standard
FvTPS6
FaTPS10
FaTPS7
14
15
in vitro enzyme assays
in vivo co-expression enzyme assays
Individually Scaled Total Ion Chromatograms
18
19 min
20
22 min
16
Trans-Nerolidol
α-farnesene
FvTPS6 KI:1670
Bulnesol
KI: 1763
β-acoradienol
KI: 1743
Khusimol
KI: 1882
8,13-cedranediol
(Z,E)-α-Farnesene
E-γ-bisabolene
Chenopodiol <α->
KI: 1861
KI: 1868
Cis, trans - farnesene
Dauca-4(11),7-diene <trans>
KI:1558
2,3-dihydro farnesyl acetate
All trans farnesyl acetate
Ion Abundance
[m/z]

**Supplementary Fig. S11:** Functional characterization of TPS-g and TPS-b clade terpene synthases (TPSs). (A-D) GC-MS traces of products resulting from either in vitro enzyme assays of individual re combinant TPSs with farnesyl diphosphate (FPP) as a substrate (gray) or co-expression assays of individual TPSs and a FPP synthase in E. coli (white). (E) Mass spectra of enzyme products identi fied by comparison to authentic standards (white) or mass spectral databases (NIST, v17.1; purple ). The following compounds are likely degradation products of FPP produced in *E. coli* cultures: (i) *Cis-trans*-farnesene; (ii) Farnesol; (iii) 2,3- dihydro farnesyl acetate; (iv) *Trans*- farnesyl

l acetate. FaTPS17, TPS18, and TPS42 taken from *F. ananassa* (Fa) cultivar 'Royal Royce'; Fv rTPS17 *F. virginiana* (Fvr ) accession 'NC_ 96-35-2'; FcTPS18 *F. chiloensis* (Fc) ecotype 'Ambato'; FvrTPS40 accession 'Harris Springs'; FcTPS40 and F cTPS52 ecotype 'Isle de Lemuy'; FvTPS6 and FvTPS50 *F. vesca* (Fv) accession 'UC06'; FaTPS7 cultivar 'Primella'; FaTPS10 cultivar 'EarliMiss'.

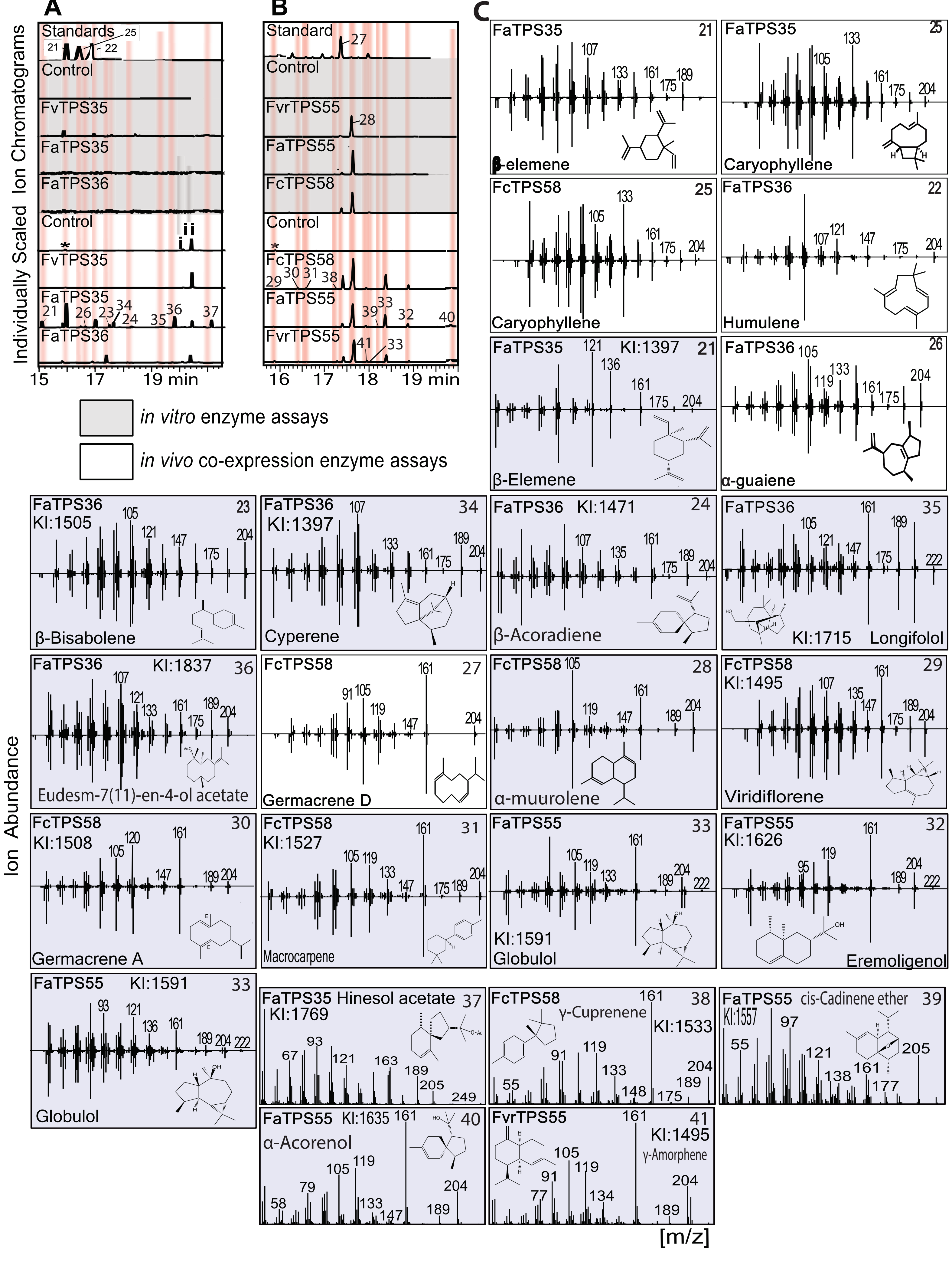
A
B
C
Individually Scaled Ion Chromatograms
Standards
Standard
Control
FvTPS35
FaTPS35
FaTPS36
FvrTPS55
FaTPS55
FcTPS58
15
17
19 min
16
18
in vitro enzyme assays
in vivo co-expression enzyme assays
Ion Abundance
[m/z]
FaTPS35 21 β-elemene
FaTPS35 25 Caryophyllene
FcTPS58 25 Caryophyllene
FaTPS36 22 Humulene
FaTPS35 KI:1397 21 β-Elemene
FaTPS36 26 α-guaiene
FaTPS36 KI:1505 23 β-Bisabolene
FaTPS36 KI:1397 34 Cyperene
FaTPS36 KI:1471 24 β-Acoradiene
FaTPS36 35 KI:1715 Longifolol
FaTPS36 KI:1837 36 Eudesm-7(11)-en-4-ol acetate
FcTPS58 27 Germacrene D
FcTPS58 28 α-muurolene
FcTPS58 KI:1495 29 Viridiflorene
FcTPS58 KI:1508 30 Germacrene A
FcTPS58 KI:1527 31 Macrocarpene
FaTPS55 33 KI:1591 Globulol
FaTPS55 KI:1626 32 Eremoligenol
FaTPS55 KI:1591 33 Globulol
FaTPS35 Hinesol acetate 37 KI:1769
FcTPS58 38 γ-Cuprenene KI:1533
FaTPS55 cis-Cadinene ether 39 KI:1557
FaTPS55 KI:1635 40 α-Acorenol
FvrTPS55 41 KI:1495 γ-Amorphene

**Supplementary Fig. S12:** Functional characterization of TPS-a terpene synthases (TPSs). (A-B) GC- MS traces of products resulting from either in vitro enzyme assays of individual recombinant TPSs with farnesyl diphosphate (FPP) as a substrate (gray) or co-expression assays of individual TPSs and a FPP synthase in E. coli (white). (C) Mass spectra of enzyme products identifie d by comparison to authentic standards (white) or mass spectral databases (NIST, v17.1; pu rple). The following compounds are likely degradation products of FPP produced in *E. co*li cultu res: (i) *Cis-trans*-farnesene; (ii) Farnesol. FvTPS35 from *F. vesca* (Fv) accession 'UC06'; FaTPS35 f rom *F. x ananassa* (Fa) cultivar 'Royal Royce'; FaTPS36 from *F. ananassa* (Fa) 'Direktor Paul Wallbaum'; FvrTPS55 *F. virginiana* (Fvr ) accession 'NC_96-35-2'; FaTPS55 cultivar 'M adame Moutot', FcTPS58 *F. chiloensis* (Fc) ecotype 'Ambato'.

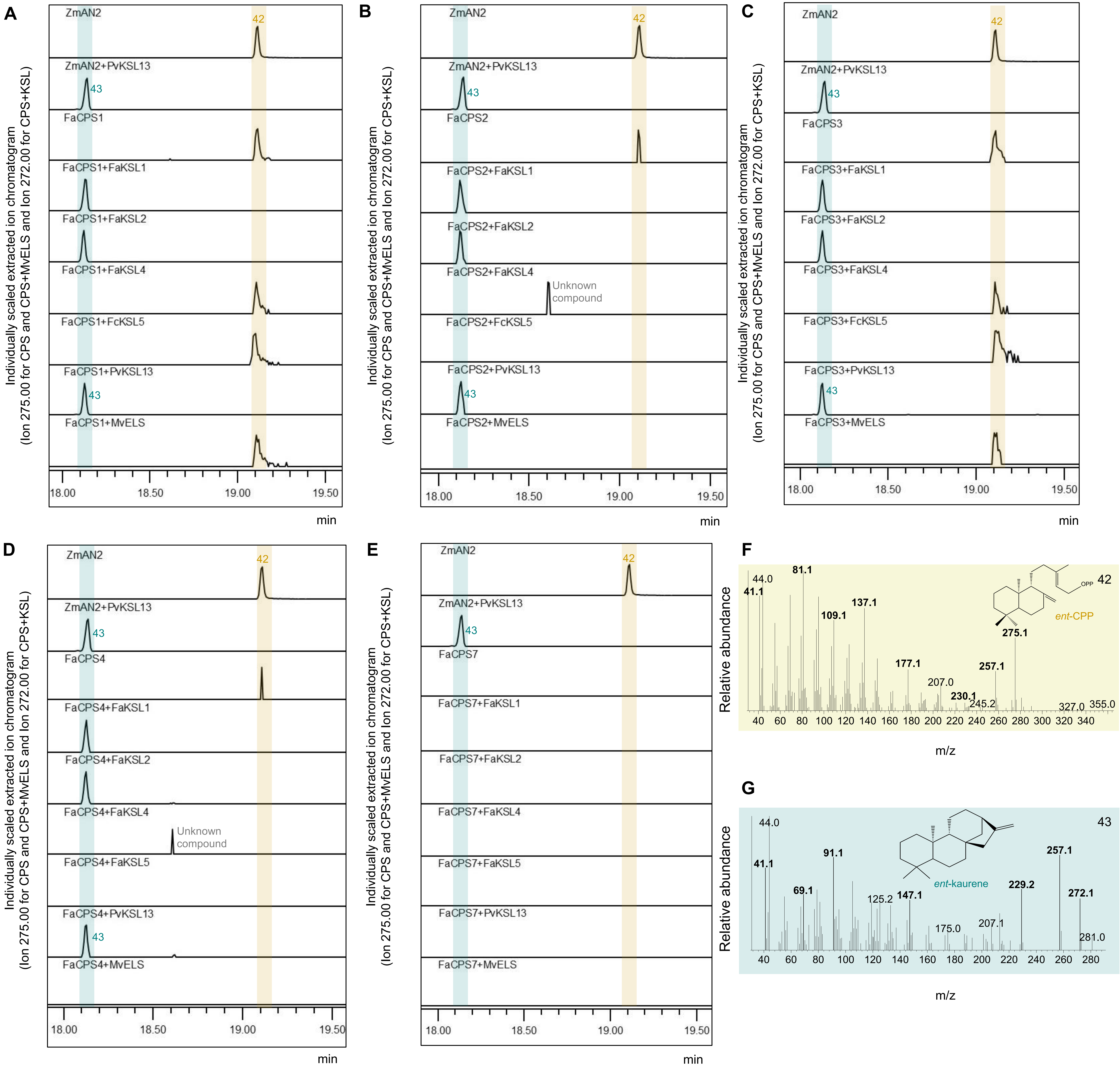

A
B
C
D
E
F
G
ZmAN2
ZmAN2+PvKSL13
FaCPS1
FaCPS1+FaKSL1
FaCPS1+FaKSL2
FaCPS1+FaKSL4
FaCPS1+FcKSL5
FaCPS1+PvKSL13
FaCPS1+MvELS
FaCPS2
FaCPS2+FaKSL1
FaCPS2+FaKSL2
FaCPS2+FaKSL4
FaCPS2+FcKSL5
FaCPS2+PvKSL13
FaCPS2+MvELS
FaCPS3
FaCPS3+FaKSL1
FaCPS3+FaKSL2
FaCPS3+FaKSL4
FaCPS3+FcKSL5
FaCPS3+PvKSL13
FaCPS3+MvELS
FaCPS4
FaCPS4+FaKSL1
FaCPS4+FaKSL2
FaCPS4+FaKSL4
FaCPS4+FaKSL5
FaCPS4+PvKSL13
FaCPS4+MvELS
FaCPS7
FaCPS7+FaKSL1
FaCPS7+FaKSL2
FaCPS7+FaKSL4
FaCPS7+FaKSL5
FaCPS7+PvKSL13
FaCPS7+MvELS
42
43
Unknown compound
Individually scaled extracted ion chromatogram
(Ion 275.00 for CPS and CPS+MvELS and Ion 272.00 for CPS+KSL)
18.00
18.50
19.00
19.50
min
Relative abundance
m/z
ent-CPP
OPP
44.0
41.1
81.1
109.1
137.1
275.1
177.1
257.1
207.0
230.1
245.2
327.0
355.0
40 60 80 100 120 140 160 180 200 220 240 260 280 300 320 340
ent-kaurene
44.0
41.1
91.1
69.1
125.2
147.1
175.0
207.1
229.2
257.1
272.1
281.0
40 60 80 100 120 140 160 180 200 220 240 260 280

**Supplementary Fig. S13** Characterization of diterpene synthase (FaCPS1-4 and FaCPS7) products. GC-MS traces of products resulting from co-expression assays of **(A)** FaCPS1, FaKSL1-4 and FcKSL5, **(B)** FaCPS2, FaKSL1-4 and FcKSL5 **(C)** FaCPS3, FaKSL1-4 and FcKSL5, **(D)** FaCPS4, FaKSL1-4 and FcKSL5, and **(E)** FaCPS7, FaKSL1-4 and FcKSL5 as compared to authentic standards produced by the ent-CPP synthase Zea mays AN2 (ZmAN2; Harris et al., 2005) and *ent*-kaurene synthase from switchgrass (*Panicum virgatum*), PvKSL13 (Pelot et al., 2018). (F) and (G)Mass spectra of enzyme products identified by comparison to enzyme-produced standards. FaCPS1-4, FaCPS7, and FaKSL1-4 taken from *F. x ananassa (Fa)* cultivar 'Royal Royce'; FvrCPS5 *F. virginiana (Fvr)* accession 'NC_96-35-2'; FcKSL5 *F. chiloensis (Fc)* ecotype 'Ambato'. 31, *ent*-CPP; 32, *ent*-kaurene.

**Supplementary Fig. S14** Functional characterization of diterpene synthases (diTPSs). (**A**) GC-MS traces of products resulting from co-expression assays of FaCPS5 or FvrCPS5 as compared to authentic standards produced by the *ent*-CPP synthase *Zea mays* AN2 (*ZmAN2*; Harris et al., 2005), the *ent-neo-cis-trans*-CLPP synthase *Panicum virgatum CPS1* (*PvCPS1*; Pelot et al., 2018) and the *PvCPS1* variant *PvCPS1:F251V* producing *ent-neo-cis-cis*-CLPP and *ent-neo-cis-trans*-CLPP (Pelot et al., 2016). (**B**) GC-MS traces of products resulting from co-expression assays of *FaKSL1-4* and *FcKSL5* with the *ent*-CPP synthase *Zea mays AN2* (*ZmAN2*; Harris et al., 2005). (**C**) GC-MS traces of products resulting from co-expression assays of specialized *P. virgatum* diTPSs tested in combinations with predicted FaKSLs to examine possible specialized FaCPS5 function. (**D**) Mass spectra of enzyme products identified by comparison to enzyme-produced standards. FaCPS5, and FaKSL1-4 taken from *F.* x *ananassa (Fa)* cultivar 'Royal Royce'; FvrCPS5 *F. virginiana* (*Fvr* ) accession 'NC_96-35-2'; FcKSL5 *F. chiloensis* (*Fc*) ecotype 'Ambato'.

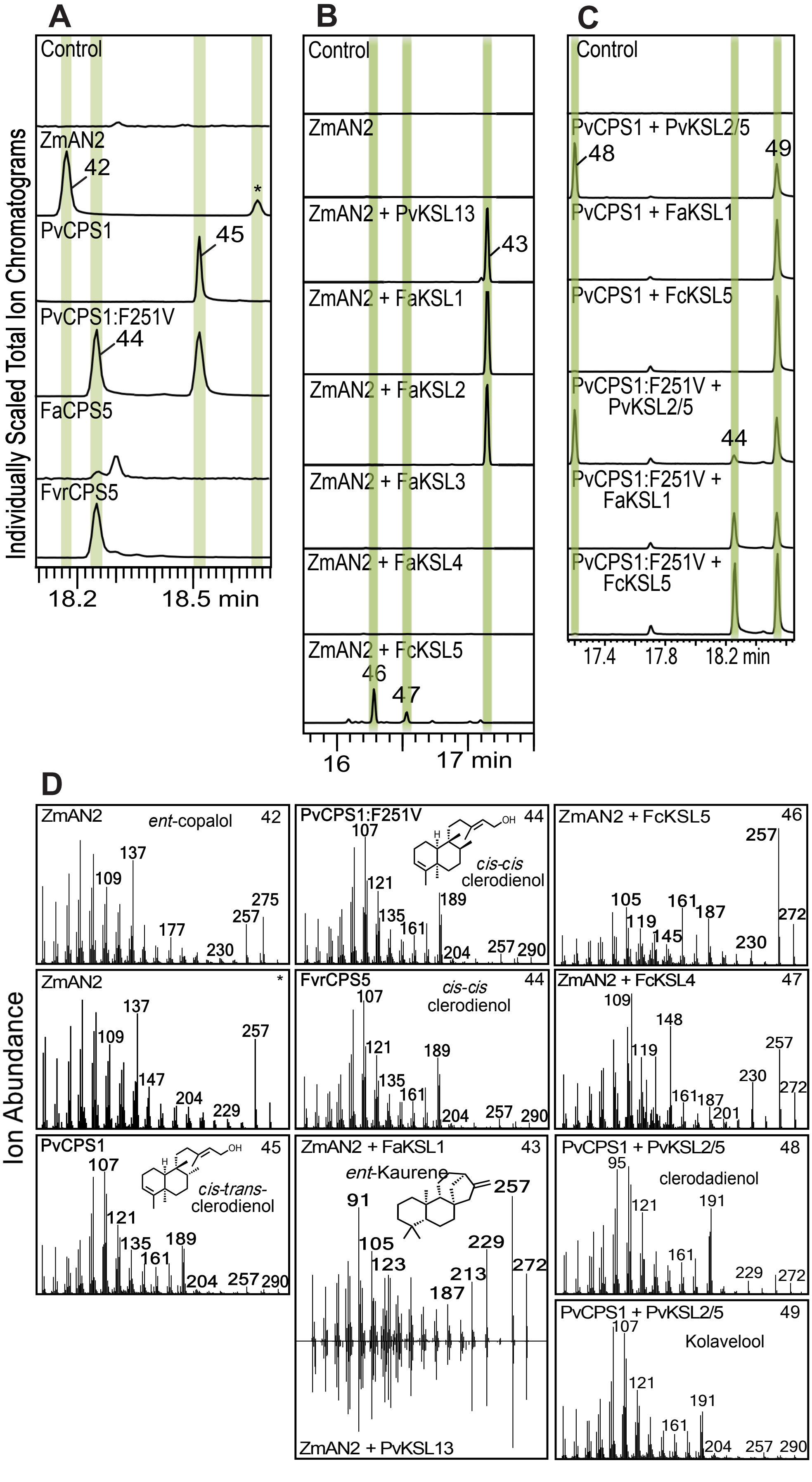

**Supplementary Fig. S15** Total SPME-GC-MS chromatograms of a sample from each of the 4 *Fragaria* species represented in this study. 31 terpenes were identified and are shown and listed with retention times. Standard confirmed terpenes are labeled in red and bold, best spectral matches in blue. Non-terpene peaks shown are also listed. (**A**) Individually scaled extracted ion chromatograms for all terpenes found via SPME-GC-MS for monoterpene ion 136 and sesquiterpene ion 204. (**B**) Small or missing terpene peaks from TIC are shown. (**C**) Total ion chromatograms shown for ripe, *Fa* Royal Royce samples processed from freshly harvested field-grown fruits, flash-frozen field-grown fruits, and flash-frozen greenhouse-grown fruits. (**D**) NIST spectral matches with standard confirmed terpenes in white (NIST, v17.1).

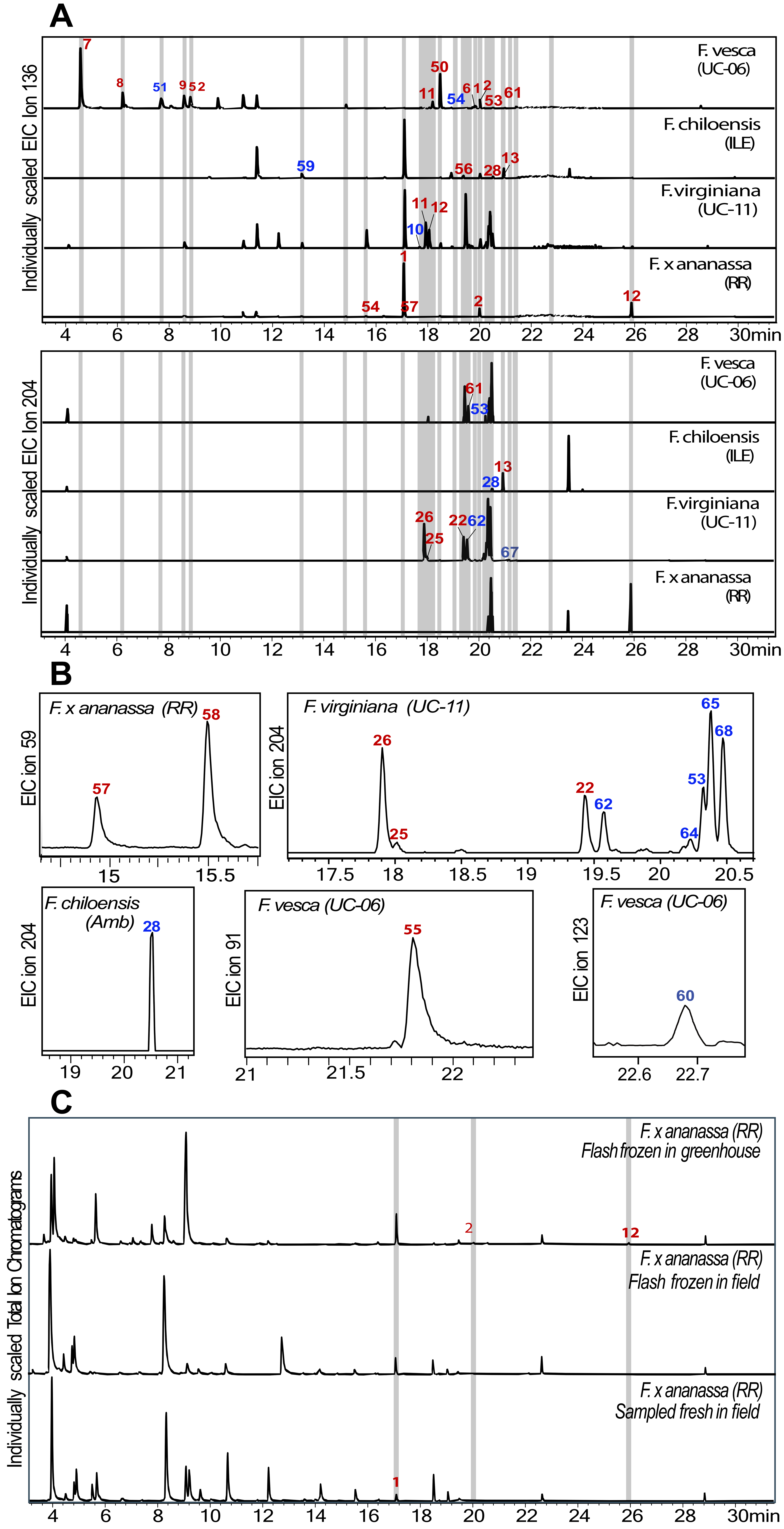


**1. Linalool (17.0)**
**2. α-Terpineol (20.0)**
**7. α-Pinene (4.4)**
**8. β-Pinene (6.2)**
**9. D-Limonene (8.4)**
**11. Terpinen-4-ol (18.1)**
**12. Nerolidol (25.9)**
**13. α-Farnesene (20.9)**
**22. Humulene (19.4)**
**25. Caryophyllene (18.0)**
**26. α-Guaiene (17.9)**
**28.** α-Muurolene (20.5)
**50. 1-R-Myrtenal (18.6)**
**51.** β-Myrcene (7.5)
**52. β-Phellandrene(8.7)**
**53.** β-Selinene (20.3)
**54.** Trans-Pinocarvyl Acetate (19.0)
**55. (-)-Myrtenol (21.7)**
**56. E-β-Farnesene (19.3)**
**57. Cis-Linalool Oxide (14.7)**
**58. Trans-Linalool Oxide (15.4)**
**59.** Allo-Ocimene (13.1)
**60.** Geraniol (22.6)
**61. Myrtenyl Acetate (19.7)**
**62.** 4,11Selinadiene (19.6)
**63.** Isopulegol (17.7)
**64.** Eremophilene (20.2)
**65.** δ-Guaiene (20.4)
**67.** 7-epi-α-Selinene (21.3)
**68.** Selinane (20.5)
**69.** Trans-Myrtanyl Acetate (20.9)

Non-Terpene Peaks
(4.0) Silica Fiber Contamination
(5.0) Silica Fiber Contamination
(8.1) Silica Fiber Contamination
(9.8) Ethyl Hexanoate
(10.8) Hexyl Acetate
(11.5) Tridecane
(12.3) Trans-2-Hexenyl Acetate
(16.3) Silica Fiber Contamination
(18.1) Methyl Caproate
(18.5) Benzyl Acetate
(18.9) Ethyl Caproate
(19.5) Trans-2-Hexenyl-Caproate
(20.5) Benzyl Acetate
(21.3) Methyl Salicylate
(22-23) Silica Fiber Contamination
(28.7) Internal Standard
(30) Silica Fiber Contamination

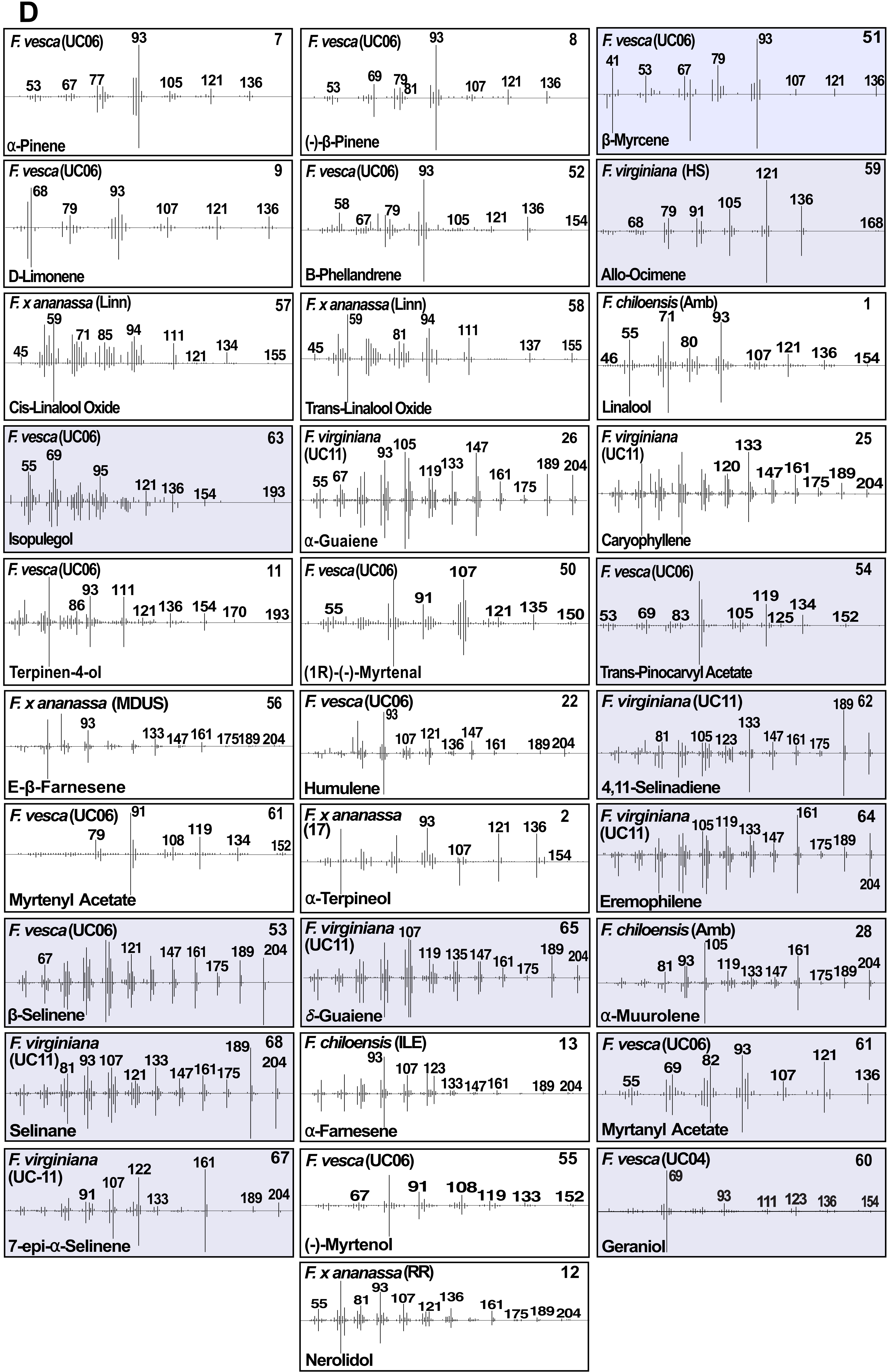
D
F. vesca (UC06) 7 α-Pinene 53 67 77 93 105 121 136
F. vesca (UC06) 8 (-)-β-Pinene 53 69 79 81 93 107 121 136
F. vesca (UC06) 51 β-Myrcene 41 53 67 79 93 107 121 136
F. vesca (UC06) 9 D-Limonene 68 79 93 107 121 136
F. vesca (UC06) 52 B-Phellandrene 58 67 79 93 105 121 136 154
F. virginiana (HS) 59 Allo-Ocimene 68 79 91 105 121 136 168
F. x ananassa (Linn) 57 Cis-Linalool Oxide 45 59 71 85 94 111 121 134 155
F. x ananassa (Linn) 58 Trans-Linalool Oxide 45 59 81 94 111 137 155
F. chiloensis (Amb) 1 Linalool 46 55 71 80 93 107 121 136 154
F. vesca (UC06) 63 Isopulegol 55 69 95 121 136 154 193
F. virginiana (UC11) 26 α-Guaiene 55 67 93 105 119 133 147 161 175 189 204
F. virginiana (UC11) 25 Caryophyllene 120 133 147 161 175 189 204
F. vesca (UC06) 11 Terpinen-4-ol 86 93 111 121 136 154 170 193
F. vesca (UC06) 50 (1R)-(-)-Myrtenal 55 91 107 121 135 150
F. vesca (UC06) 54 Trans-Pinocarvyl Acetate 53 69 83 105 119 125 134 152
F. x ananassa (MDUS) 56 E-β-Farnesene 93 133 147 161 175 189 204
F. vesca (UC06) 22 Humulene 93 107 121 136 147 161 189 204
F. virginiana (UC11) 62 4,11-Selinadiene 81 105 123 133 147 161 175 189
F. vesca (UC06) 61 Myrtenyl Acetate 79 91 108 119 134 152
F. x ananassa (17) 2 α-Terpineol 93 107 121 136 154
F. virginiana (UC11) 64 Eremophilene 105 119 133 147 161 175 189 204
F. vesca (UC06) 53 β-Selinene 67 121 147 161 175 189 204
F. virginiana (UC11) 65 δ-Guaiene 107 119 135 147 161 175 189 204
F. chiloensis (Amb) 28 α-Muurolene 81 93 105 119 133 147 161 175 189 204
F. virginiana (UC11) 68 Selinane 81 93 107 121 133 147 161 175 189 204
F. chiloensis (ILE) 13 α-Farnesene 93 107 123 133 147 161 189 204
F. vesca (UC06) 61 Myrtanyl Acetate 55 69 82 93 107 121 136
F. virginiana (UC-11) 67 7-epi-α-Selinene 91 107 122 133 161 189 204
F. vesca (UC06) 55 (-)-Myrtenol 67 91 108 119 133 152
F. vesca (UC04) 60 Geraniol 69 93 111 123 136 154
F. x ananassa (RR) 12 Nerolidol 55 81 93 107 121 136 161 175 189 204

**Supplementary Fig. S16** Abundance of terpene metabolites and relevant terpene synthase (TPS) genes in field-grown strawberry fruits which were flash frozen immediately after harvest for transcriptomic analysis. (**A**) Dot plot representing average normalized peak area of terpenes identified via SPME-GC-MS analysis of metabolites extracted from harvested from flash frozen tissue. (**B**) Hierarchical cluster analysis performed on gene expression data of select TPS genes analyzed in this study. TPSs were identified based on mapping functionally characterized TPSs against the FaRR1 genome (Hardigan et al. 2021). Scaled gene expression data are based on three biological replicates. *F.* x *ananassa (Fa)* cultivars 'Royal Royce' (RR), '17C224P011' (17), 'Mara des Bois' (MDB) for which no field-grown transcriptome data was available, 'Beaver Belle' (BB), 'MDUS 5130' (MDUS), 'Tangi' (Tan), 'Primella' (Prim), 'Linn', 'Headliner' (Head), 'EarliMiss' (EM), 'Direktor Paul Wallbaum' (DPW), and 'Madame Moutot' (MM). *F. chiloensis* (*Fc*) ecotypes 'Ambato' (Amb) and 'Isle de Lemuy 02A White' (ILE). *F. virginiana* (*Fvr* ) accessions 'Harris Springs' (HS), 'NC_96-35-2' (NC), 'UC11'. Diploid *F. vesca* (*Fv*) accessions 'UC04' and 'UC06'.

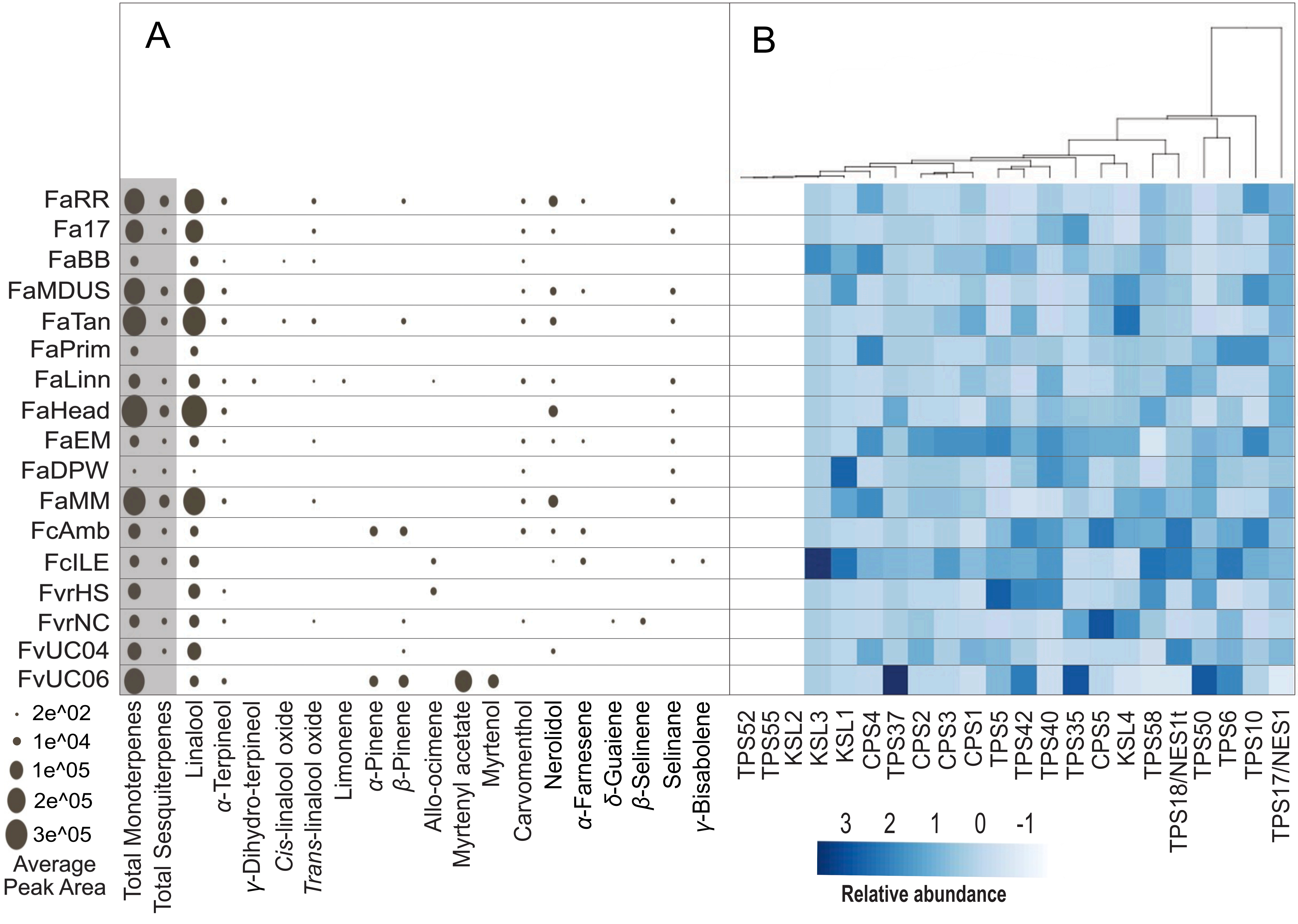

**Supplementary Fig. S17** Hierarchical cluster analysis of select upstream terpene biosynthetic and terpene synthase (TPS) genes analyzed in this study from greenhouse-grown fruit from two cultivars, *Fa* Royal Royce and Mara des Bois, harvested from various developmental stages. TPSs were identified based on mapping functionally characterized TPSs in study against the FaRR1 genome (Hardigan et al. 2021). Scaled gene expression data are based on three biological replicates.

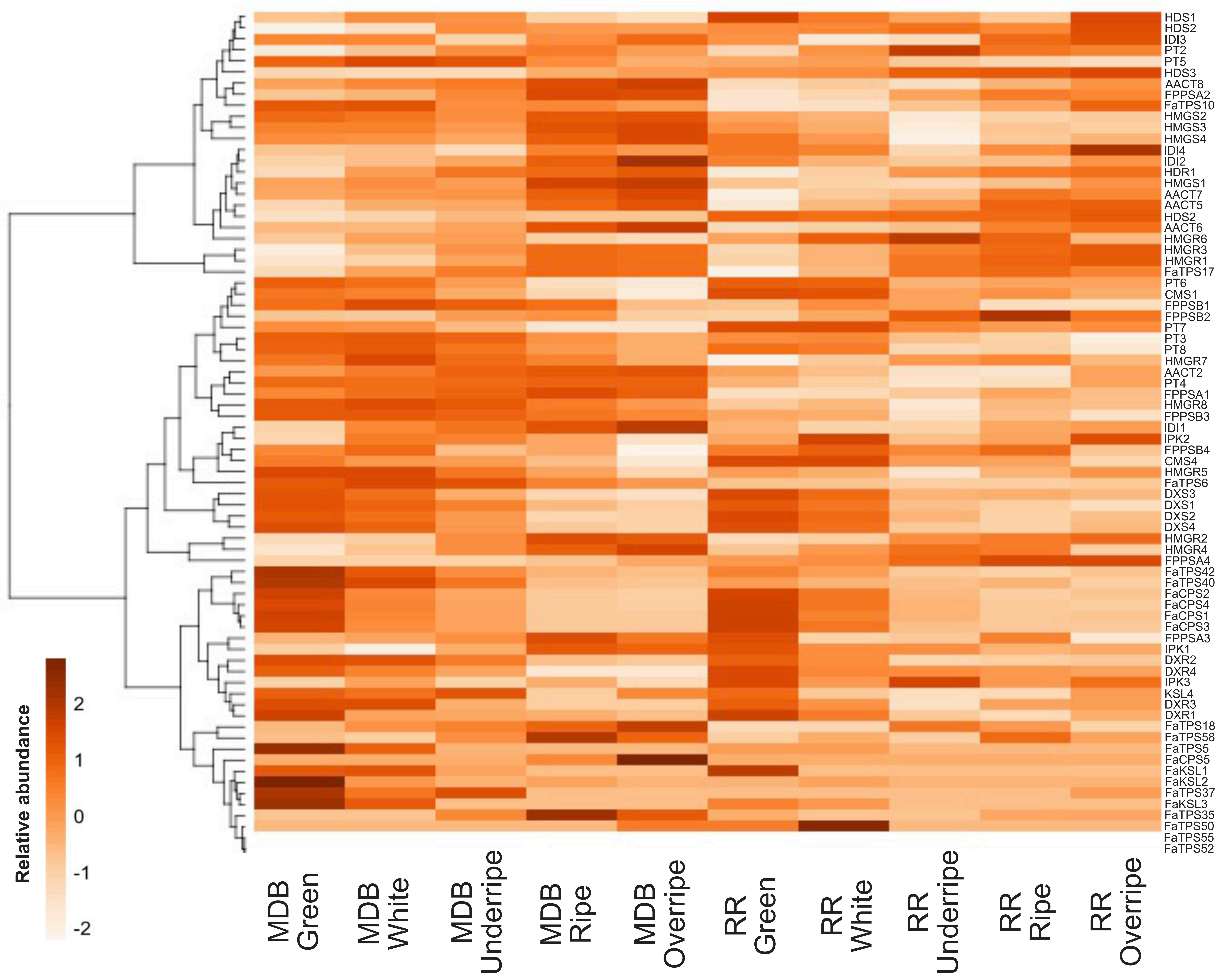

Supplement: kiag292_Supplementary_Data [file kiag292_supplementary_data.zip › PLPHYS-2025-1377_R2 Supplementary Figures.pdf]
