# Supplementary material for: Genome-wide discovery and characterization of terpene synthases contributing to strawberry aroma metabolism
Source: Plant Physiol. 2026 May 22;201(3):kiag292. doi: 10.1093/plphys/kiag292 (PMC13368613; doi:10.1093/plphys/kiag292)
Supplement: kiag292_Supplementary_Data [file kiag292_supplementary_data.zip › PLPHYS-2025-1377_R2 Supplementary Tables.pdf]

Supplementary Table S3 Terpene synthase sequences identified in this study. 64 Genomic *F.* × *ananassa (Fa)* MSTP S; 12 Genomic *F.* × *ananassa* diTPS; 4 additional *F.* × *ananassa* transcripts; 5 additional *F. chiloensis (Fc)* transcripts; 5 additional *F. virginiana (Fvr)* transcripts; 4 additional *F. vesca (Fv)* transcripts. Additionally discarded pseudogenes are included from synteny analysis. Functionally tested candidates are shown in bold.

**FaRR1 Genome Hit**	**Type**	**Name**	**Synteny Group**	**Species**	***F. vesca Hit***	**RRX8W**	**DDXXD**	**AA**	Tested?
Fxa1Ag100480	M/S TPS	*FaTPS1*	a	*F. x ananassa*	FvH4_1g05400	RRTANFKSPVW	DDMYD	557	No
Fxa1Ag100363	Syntenic Psuedogene	(Fragment)	b	*F. x ananassa*	FvH4_1g04000	NA	DDIYD	425	
Fxa1Bg200351	M/S TPS	*FaTPS2*	b	*F. x ananassa*	FvH4_1g04000	RRSANYHPSIW	DDIYD	560	No
Fxa1Dg200965	M/S TPS	*FaTPS3*	c	*F. x ananassa*	-	NA	DDIFD	557	No
Fxa2Ag102276	diTPS	*FaCPS6*	d	*F. x ananassa*	-	NA	DIDD	563	No
**Fxa2Ag102357**	**diTPS**	***FaCPS7***	**e**	***F. x ananassa***	**FvH4_2g23400**	**NA**	**DLDD**	**809**	**Yes**
Fxa2Cg203098	Syntenic Psuedogene	(Fragment)	e	*F. x ananassa*	FvH4_2g23400	NA		113	
**Fxa2Ag102359.1**	**diTPS**	***FaCPS1***	**f**	***F. x ananassa***	**FvH4_2g23440**	**NA**	**DIDD**	**818**	**Yes**
**Fxa2Bg202200.1**	**diTPS**	***FaCPS2***	**f**	***F. x ananassa***	**FvH4_2g23440**	**NA**	**DIDD**	**818**	**Yes**
**Fxa2Cg203102.1**	**diTPS**	***FaCPS3***	**f**	***F. x ananassa***	**FvH4_2g23440**	**NA**	**DIDD**	**818**	**Yes**
**Fxa2Dg202041.1**	**diTPS**	***FaCPS4***	**f**	***F. x ananassa***	**FvH4_2g23440**	**NA**	**DIDD**	**818**	**Yes**
Fxa2Bg200794	M/S TPS	*FaTPS4*	g	*F. x ananassa*	-	RPIANFQPSIW	DDIYD	582	No
Fxa2Ag100915	Syntenic Psuedogene	(Fragment)	h	*F. x ananassa*	-	NA	DDIYD	408	
Fxa2Bg200800	M/S TPS	*FaTPS5*	h	*F. x ananassa*	-	RPIANFQPSIW	DDIYD	615	No
**Fxa2Bg200800.1**	M/S TPS	***FvrTPS5***	h	*F. virginiana*	**NA**	**RPIANFQPSIW**	**DDIYD**	**498**	**Yes**
Fxa3Ag100266.1	M/S TPS	*FaTPS6*	i	*F. x ananassa*	FvH4_3g03000	RRSANYKPNIW	DDVYD	584	No
**Fxa3Ag100266.1**	M/S TPS	***FvTPS6***	i	*F. vesca*	**FvH4_3g03000**	**RRSANYKPNIW**	**DDVYD**	**583**	**Yes**

**Fxa3Ag100266.1**	M/S TPS	***FaTPS7***	i	*F. x ananassa*	**FvH4_3g03000**	**RRSANYKPNIW**	**DDVYD**	**584**	**Yes**
**Fxa3Bg200271.1**	M/S TPS	***FaTPS10***	i	*F. x ananassa*	**FvH4_3g03000**	**RRSANYKPNIW**	**DDVYD**	**568**	**Yes**
Fxa3Dg200246.1	M/S TPS	*FaTPS21*	i	*F. x ananassa*	FvH4_3g03000	RRSANYKPNIW	DDVYD	580	No
Fxa3Ag100275.1	M/S TPS	*FaTPS8*	j	*F. x ananassa*	FvH4_3g03061	NA	DDIFD	522	No
Fxa3Bg200281.1	M/S TPS	*FaTPS12*	j	*F. x ananassa*	FvH4_3g03061	RRGIAEDSLLP	DDIFD	581	No
Fxa3Dg200253.1	M/S TPS	*FaTPS22*	j	*F. x ananassa*	FvH4_3g03061	NA	DDIFD	509	No
**Fxa3Ag101306**	diTPS	***FaKSL1***	k	*F. x ananassa*	**FvH4_3g14116**	**NA**	**DDFFD**	**797***	**Yes**
**Fxa3Bg201184**	diTPS	***FaKSL2***	k	*F. x ananassa*	**FvH4_3g14116**	**NA**	**DDFFD**	**797***	**Yes**
**Fxa3Cg101173**	diTPS	***FaKSL3***	k	*F. x ananassa*	**FvH4_3g14116**	**NA**	**DDFFD**	**774***	**Yes**
**Fxa3Dg2011124**	diTPS	***FaKSL4***	k	*F. x ananassa*	**FvH4_3g14116**	**NA**	**DDFFD**	**787***	**Yes**
**Fxa3Dg201124**	diTPS	***FcKSL5***	k	*F. chiloensis*	**FvH4_3g14116**	**NA**	**DDLFD**	**571**	**Yes**
Fxa3Ag102016.1	M/S TPS	*FaTPS9*	l	*F. x ananassa*	FvH4_3g21390	NA	DDVYD/DDIFD	1095	No
Fxa3Bg201803	Syntenic Psuedogene	(Fragment)	l	*F. x ananassa*	FvH4_3g21390	NA	DDIFD	329	
Fxa3Cg101786.1	M/S TPS	*FaTPS19*	l	*F. x ananassa*	FvH4_3g21390	NA	DDVYD/DDIFD	1019	No
Fxa3Dg201726.1	M/S TPS	*FaTPS23*	l	*F. x ananassa*	FvH4_3g21390	NA	DDIFD	557	No
Fxa3Bg200279.1	M/S TPS	*FaTPS11*	m	*F. x ananassa*	-	RRGIAEDSLLP	DDIFD	585	No
Fxa3Bg200284.1	M/S TPS	*FaTPS13*	n	*F. x ananassa*	-	NA	DDIFD	485	No
Fxa3Bg200286.1	M/S TPS	*FaTPS14*	o	*F. x ananassa*	-	NA	DDIFD	522	No
Fxa3Bg201829.1	M/S TPS	*FaTPS15*	p	*F. x ananassa*	FvH4_3g21490	NA	DDIYD	484	No
Fxa3Cg100132.1	M/S TPS	*FaTPS16*	q	*F. x ananassa*	FvH4_3g01590	RRSVNFKPSIW	DDMYD	562	No
**Fxa3Cg100266.2**	M/S TPS	***FvrTPS17***	r	*F. virginiana*	**(FvH4_3g03041)**	**RRGIAEDSLLP**	**DDIFD**	**495**	**Yes**
**Fxa3Cg100266.2**	M/S TPS	***FaTPS17***	r	*F. x ananassa*	**(FvH4_3g03041)**	**NA**	**DDIFD**	**519**	**Yes**

**Fxa3Cg100266.3**	M/S TPS	***FcTPS18***	r	*F. chiloensis*	**(FvH4_3g03041)**	**RRGIAEDSLLP**	**DDIFD**	**580**	**Yes**
**Fxa3Cg100266.3**	M/S TPS	***FaTPS18***	r	*F. x ananassa*	**(FvH4_3g03041)**	**RRGIAEDSLLP**	**DDIFD**	**580**	**Yes**
Fxa3Ag102106	Syntenic Psuedogene	(Fragment)	s	*F. x ananassa*	FvH4_3g22162	NA	DDIYD	268	
Fxa3Cg101875.1	M/S TPS	*FaTPS20*	s	*F. x ananassa*	FvH4_3g22162	RRSANFHPIW	DDIYD	562	No
**Fxa3Dg201123.1**	diTPS	***FvrCPS5***	t	*F. virginiana*	**Fv4_3g14111**	**NA**	**DLDD**	**800**	**Yes**
**Fxa3Dg201123.1**	diTPS	***FaCPS5***	t	*F. x ananassa*	**Fv4_3g14111**	**NA**	**DLDD**	**800**	**Yes**
Fxa4Ag102687	Syntenic Psuedogene	(Fragment)	v	*F. x ananassa*	FvH4_4g27943	RNKIVAEYFW	DDVYD	311	
Fxa4Bg102623	Syntenic Psuedogene	(Too long)	v	*F. x ananassa*	FvH4_4g27943	RPANYAPCMW	DDVYD	879	
Fxa4Cg202367.1	M/S TPS	*FaTPS24*	v	*F. x ananassa*	FvH4_4g27943	RPANYAPCMW	DDVYD/DDVYD	1050	No
Fxa4Dg102144.1	M/S TPS	*FaTPS26*	v	*F. x ananassa*	FvH4_4g27943	RPANYAPCIW	DDVYD	564	No
Fxa4Dg102142.1	M/S TPS	*FaTPS25*	w	*F. x ananassa*	-	RCSPHYTPGIE	DDVYD	560	No
Fxa5Ag200582.1	M/S TPS	*FaTPS27*	x	*F. x ananassa*	FvH4_5g06470	RRSANFSPSVW	DDTFD	582	No
Fxa5Bg100567.1	M/S TPS	*FaTPS29*	x	*F. x ananassa*	FvH4_5g06470	RRSANFSPSVW	DDTFD	572	No
Fxa5Cg200524	M/S TPS	*FaTPS31*	x	*F. x ananassa*	FvH4_5g06470	RRSANFPPSVW	DDTFD	570	No
Fxa5Dg200537	M/S TPS	*FaTPS34*	x	*F. x ananassa*	FvH4_5g06470	RRSADFSPSVW	DDTFD	573	No
**Fxa5Dg200537.1**	M/S TPS	***FaTPS35***	x	*F. x ananassa*	**FvH4_5g06470**	**RRSADFSPSVW**	**DDTFD**	**570**	**Yes**
**Fxa5Dg200537.1**	M/S TPS	***FvTPS35***	x	*F. vesca*	**FvH4_5g06470**	**RRSADFSPSVW**	**DDTFD**	**570**	**Yes**
**Fxa5Dg200537.1**	M/S TPS	***FaTPS36***	x	*F. x ananassa*	**FvH4_5g06470**	**RRSADFSPSVW**	**DDTFD**	**570**	**Yes**
Fxa5Ag203430.1	M/S TPS	*FaTPS28*	y	*F. x ananassa*	FvH4_5g35711	LAYYEYAPCIW	DDIYD	586	No
Fxa5Bg103205	M/S TPS	*FaTPS30*	y	*F. x ananassa*	FvH4_5g35711	LAYYEHAPCIW	DDIYD	579	No
Fxa5Cg202929	M/S TPS	*FaTPS32*	z	*F. x ananassa*	FvH4_5g35700	RPSADYTPSIW	DDIYD	555	No
Fxa5Dg200534	M/S TPS	*FaTPS33*	aa	*F. x ananassa*	-	RRSANFSPSIW	DDVYD	553	No

Fxa6Ag100563	M/S TPS	*FaTPS37*	bb	*F. x ananassa*	FvH4_6g06540	RPIANFQPSIW	DDIYD	579	No
**Fxa6Ag100563.1**	M/S TPS	***FvTPS37***	bb	*F. vesca*	**FvH4_6g06540**	**RPIANFQPSIW**	**DDIYD**	**569**	**Yes**
Fxa6Bg100527	M/S TPS	*FaTPS46*	bb	*F. x ananassa*	FvH4_6g06540	RPIANFQPSIW	DDIYD	580	No
Fxa6Ag101058	M/S TPS	*FaTPS38*	cc	*F. x ananassa*	FvH4_6g11440	PRPANYAPCIW	DDVYD	673	No
Fxa6Bg100956	Syntenic Psuedogene	(Fragment)	cc	*F. x ananassa*	FvH4_6g11440	NA	DDVYD	392	
Fxa6Cg100917	M/S TPS	*FaTPS47*	cc	*F. x ananassa*	FvH4_6g11440	PRPANYAPCIW	DDVYD	564	No
Fxa6Dg100848	M/S TPS	*FaTPS49*	cc	*F. x ananassa*	FvH4_6g11440	PRPANYAPCIW	DDVYD	564	No
Fxa6Ag101852	M/S TPS	*FaTPS39*	dd	*F. x ananassa*	FvH4_6g19050	RRTANYHPSIW	DDIYD	564	No
Fxa6Cg101643	M/S TPS	*FaTPS48*	dd	*F. x ananassa*	FvH4_6g19050	RRTAIYHPSIW	DDIYD	750	No
Fxa6Dg101605	M/S TPS	*FaTPS50*	dd	*F. x ananassa*	FvH4_6g19050	RRTANFKPSVW	DDMYD	558	No
**Fxa6D101605.1**	M/S TPS	***FvTPS50***	dd	*F. vesca*	**FvH4_6g19050**	**RRTANFKPSVW**	**DDMYD**	**556**	**Yes**
Fxa6Ag104161	M/S TPS	*FaTPS40*	ee	*F. x ananassa*	FvH4_6g43720	RRLAQYHPTIW	DDMYD	614	No
**Fxa6Ag104161.1**	M/S TPS	***FcTPS40***	ee	*F. chiloensis*	**FvH4_6g43720**	**RRLAQYHPTIW**	**DDMYD**	**602**	**Yes**
**Fxa6Ag104161.1**	M/S TPS	***FvrTPS40***	ee	*F. virginiana*	**FvH4_6g43720**	**RRLAQYHPTIW**	**DDMYD**	**604**	**Yes**
Fxa6Bg103821	Syntenic Psuedogene	(Fragment)	ee	*F. x ananassa*	FvH4_6g43720	NA	DDMYD	395	
Fxa6Cg103709	Syntenic Psuedogene	(Fragment)	ee	*F. x ananassa*	FvH4_6g43720	RRLAQYHPTIW	NA	334	
Fxa6Dg103609	M/S TPS	*FaTPS51*	ee	*F. x ananassa*	FvH4_6g43720	RRLAQYHPTIW	DDMYD	630	No
Fxa6Ag104162	M/S TPS	*FaTPS41*	ff	*F. x ananassa*	-	RRLAQYHPTIW	DDMYD	589	No
**Fxa6Ag104162.1**	M/S TPS	***FaTPS42***	ff	*F. x ananassa*	**-**	**RRLAQYHPTIW**	**DDMYD**	**580**	**Yes**
Fxa6Ag104168	M/S TPS	*FaTPS43*	gg	*F. x ananassa*	-	RRTADYKPSIW	DDVYD/DDVYD	1067	No
Fxa6Ag104224	M/S TPS	*FaTPS44*	hh	*F. x ananassa*	-	TRTADYKPSIW	DDVYD	572	No
Fxa6Cg103766	Syntenic Psuedogene	(Fragment)	hh	*F. x ananassa*	-	RTADYKPSIW	DDVYD	363	
Fxa6Ag104324	M/S TPS	*FaTPS45*	ii	*F. x ananassa*	-	RVTPDYKPSIW	DDIYD	563	No

Fxa6Bg103826	Syntenic Psuedogene	(Fragment)	jj	*F. x ananassa*	FvH4_6g43820	NA	DDVYD	259	
Fxa6Dg103617	M/S TPS	*FaTPS52*	jj	*F. x ananassa*	FvH4_6g43820	RRTTDYKPSIW	DDVYD	578	No
**Fxa6Dg103617.2**	M/S TPS	***FcTPS52***	jj	*F. chiloensis*	**FvH4_6g43820**	**RRTTDYKPSIW**	**DDVYD**	**577**	**Yes**
Fxa6Dg103674	M/S TPS	*FaTPS53*	kk	*F. x ananassa*	-	TRTAYYKPSIW	DDVYD	566	No
Fxa7Ag200397	M/S TPS	*FaTPS54*	ll	*F. x ananassa*	FvH4_7g03620	RRVANFSPSVW	DDIYD	528	No
Fxa7Bg200423	M/S TPS	*FaTPS57*	ll	*F. x ananassa*	FvH4_7g03620	RRVANFSPSVW	DDIYD	564	No
Fxa7Cg100355	M/S TPS	*FaTPS59*	ll	*F. x ananassa*	FvH4_7g03620	RRVANFSPSVW	DDIYD	510	No
Fxa7Dg100343	M/S TPS	*FaTPS62*	ll	*F. x ananassa*	FvH4_7g03620	RRVANFSPSVW	DDIYD	565	No
**Fxa7Ag203289.1**	M/S TPS	***FvrTPS55***	mm	*F. virginiana*	**FvH4_7g33640**	**PAYYEYAPCIW**	**DDIYD**	**561**	**Yes**
**Fxa7Ag203289.1**	M/S TPS	***FaTPS55***	mm	*F. x ananassa*	**FvH4_7g33640**	**PAYYEYAPCIW**	**DDIYD**	**561**	**Yes**
**Fxa7Bg203106**	M/S TPS	***FcTPS58***	mm	*F. chiloensis*	**FvH4_7g33640**	**PAYYEYAPCIW**	**DDIYD**	**561**	**Yes**
Fxa7Bg203106	M/S TPS	*FaTPS58*	mm	*F. x ananassa*	FvH4_7g33640	LAYYEYAPCIW	DDIYD	568	No
Fxa7Cg103018	M/S TPS	*FaTPS61*	mm	*F. x ananassa*	FvH4_7g33640	LAYYEYAPCIW	DDIYD	584	No
Fxa7Dg102851	M/S TPS	*FaTPS64*	mm	*F. x ananassa*	FvH4_7g33640	LAYYEYAPGIW	DDIYD	568	No
Fxa7Ag203294	M/S TPS	*FaTPS56*	nn	*F. x ananassa*	FvH4_7g33760	PAYYEYAPCIW	DDIYD	1910	No
Fxa7Bg203107	Syntenic Psuedogene	(Too long, no DDxxD)	nn	*F. x ananassa*	FvH4_7g33760	RRKFTGQTVA	NA	1347	
Fxa7Cg103019	Syntenic Psuedogene	(Too long, no DDxxD)	nn	*F. x ananassa*	FvH4_7g33760	NA	NA	951	
Fxa7Dg102852	Syntenic Psuedogene	(Too long, no DDxxD)	nn	*F. x ananassa*	FvH4_7g33760	RRKFTGQTVA	NA	1355	
Fxa7Cg101251	M/S TPS	*FaTPS60*	oo	*F. x ananassa*	FvH4_7g13030	RRSTNFKLSIW	DDIYD	536	No
Fxa7Dg102096	M/S TPS	*FaTPS63*	pp	*F. x ananassa*	-	RRSTNFKPSIW	DDIYD	472	No

Supplementary Table S5: Strawberry accessions used in this study. Modern cultivars 'Mara Des Bois' (MDB) and 'Royal Royce' were also grown under greenhouse conditions for a developmental time course study. No MDB fruit could be harvested in the field for RNA sequencing.

**Species**	**Type**	**Code**	**Accessions** (Identifier) Release Date & Location
F. vesca (Fv)	Diploid	UC04<br>UC06	**UC04** (PI 551598) California, USA<br>**UC06** (PI 551514) California, USA
F. virginiana (Fvr)	North American Octoploid	NC<br>HS	**NC_96-35-2** (PI 612323) Alabama, USA<br>**Harris Springs** (17X004P001) California, USA
F. chiloensis (Fc)	South American Octoploid	ILE<br>Amb	**Isle De Lemuy (02A White)** (PI 552038) Chile<br>**Ambato** (PI 551736) Ecuador
F. × ananassa (Fa)	Breeding Cultivar Octoploid	MM<br>DPW<br>EM<br>Head<br>Linn<br>Prim<br>Tan<br>MDUS<br>BB<br>MDB<br>17<br>RR	**Madame Moutot** (PI 551632) 1910 France<br>**Direktor Paul Wallbaum** (PI 551436) 1953 Germany<br>**EarliMiss** (PI 551862) 1955 Mississippi, USA<br>**Headliner** (PI 551652) 1957 Louisiana, USA<br>**Linn** (PI 551500) 1967 Oregon, USA<br>**Primella** (PI 551422) 1969 Netherlands<br>**Tangi** (PI 551481) 1975 Louisiana, USA<br>**MDUS 5130** (PI 551946) 1981 Maryland, USA<br>**Beaver Belle** (PI 551839) 1989 Canada<br>***Mara Des Bois** (17Z001P001) 1991 France<br>**17C224P011** [2017] California, USA<br>***Royal Royce** (08C123P001) 2019 California, USA

Supplementary Table S6: Amino acid sequence similarity matrices of identified/tested TPS and syntenic pseudogenes from synteny analysis.

**TPS Clade C**	**FvrCPS5**	**Fxa2Cg203098.1**	**FaCPS1**	**FaCPS2**	**FaCPS3**	**FaCPS4**	**FaCPS5**	**FaCPS6**	**FaCPS7**
**FvrCPS5**		10.9	69.2	69.2	69.2	69	98.5	54.7	78.5
**Fxa2Cg203098.1**	10.9		9.6	9.8	9.9	9.8	10.9	16.3	11.6
**FaCPS1**	69.2	9.6		96.7	96.2	96.1	69.4	51.6	71
**FaCPS2**	69.2	9.8	96.7		98.3	98.2	69.6	51.7	71.3
**FaCPS3**	69.2	9.9	96.2	98.3		98	69.6	51.7	71.7
**FaCPS4**	69	9.8	96.1	98.2	98		69.2	51.7	71.3
**FaCPS5**	98.5	10.9	69.4	69.6	69.6	69.2		55	78.4
**FaCPS6**	54.7	16.3	51.6	51.7	51.7	51.7	55		54.8
**FaCPS7**	78.5	11.6	71	71.3	71.7	71.3	78.4	54.8	

**TPS Clade e/f**	**FcKSL5**	**Fxa3Dg201124.1**	**FaKSL1**	**FaKSL2**	**FaKSL3**	**FaKSL4**
**FcKSL5**		21.9	51.6	52.1	48.8	59
**Fxa3Dg201124.1**	21.9		40.7	40.6	38.8	31.8
**FaKSL1**	51.6	40.7		97.4	93	73.9
**FaKSL2**	52.1	40.6	97.4		91.3	73.8
**FaKSL3**	48.8	38.8	93	91.3		70.6
**FaKSL4**	59	31.8	73.9	73.8	70.6	

**TPS Clade g**	**FcTPS18**	**FvNES1**	**FvrTPS17**	**Fxa3Cg100266.1**	**FaNES1**	**FaNES2**	**FaTPS8**	**FaTPS11**	**FaTPS12**	**FaTPS13**	**FaTPS14**	**FaTPS17**	**FaTPS18**	**FaTPS 22**
**FcTPS18**		93.6	82.1	23.4	88.8	93.6	83.4	92.7	94	69.2	83.4	88.8	96.2	81.6
**FvNES1**	93.6		80.5	22.6	84.2	93.8	83.4	92.8	98.1	68.9	83.4	84.2	92.8	81.8
**FvrTPS17**	82.1	80.5		19.7	73.1	82.6	70.6	81.5	80.9	75	70.4	73.1	81.1	68.4
**Fxa3Cg100266.1**	23.4	22.6	19.7		21	22.6	20.1	22.6	22.7	17.6	20.1	21	24.1	19.7
**FaNES1**	88.8	84.2	73.1	21		84.9	93.9	84.2	84.7	76.5	93.9	100	85.9	91.2
**FaNES2**	93.6	93.8	82.6	22.6	84.9		83.8	96.1	94.7	69.1	83.8	84.9	92.9	81.7
**FaTPS8**	83.4	83.4	70.6	20.1	93.9	83.8		83.4	83.3	75	98.9	93.9	82	91
**FaTPS11**	92.7	92.8	81.5	22.6	84.2	96.1	83.4		93.3	68.4	83.5	84.2	91.7	81.2
**FaTPS12**	94	98.1	80.9	22.7	84.7	94.7	83.3	93.3		69.3	83.4	84.7	93.5	82.3
**FaTPS13**	69.2	68.9	75	17.6	76.5	69.1	75	68.4	69.3		75	76.5	69.2	74
**FaTPS14**	83.4	83.4	70.4	20.1	93.9	83.8	98.9	83.5	83.4	75		93.9	82.6	91.8
**FaTPS17**	88.8	84.2	73.1	21	100	84.9	93.9	84.2	84.7	76.5	93.9		85.9	91.2
**FaTPS18**	96.2	92.8	81.1	24.1	85.9	92.9	82	91.7	93.5	69.2	82.6	85.9		81.4
**FaTPS22**	81.6	81.8	68.4	19.7	91.2	81.7	91	81.2	82.3	74	91.8	91.2	81.4	

**TPS Clade b, 1**	**FaTPS51**	**FaTPS43**	**FaTPS42**	**FaTPS41**	**FaTPS40**	**FaTPS18**	**FaTPS17**	**Fxa6Cg103709.1**	**Fxa6Bg103821.1**	**FvrTPS40**	**FcTPS40**
**FaTPS51**		20.1	89.9	87.5	75.7	31.4	30.1	41.1	60	75.7	77

**FaTPS43**	20.1		20.5	20.2	20	16.5	16.3	9.5	15.6	20.5	20.6
**FaTPS42**	89.9	20.5		93	76.6	33.7	32.6	40.9	64.8	80.8	82.9
**FaTPS41**	87.5	20.2	93		76.2	34	32.2	40.8	63.4	80.5	81.7
**FaTPS40**	75.7	20	76.6	76.2		31.9	30.2	42.3	51.7	83.2	84.3
**FaTPS18**	31.4	16.5	33.7	34	31.9		86.4	16.8	23.7	33.3	33.3
**FaTPS17**	30.1	16.3	32.6	32.2	30.2	86.4		14.9	26.5	31.8	31.9
**Fxa6Cg103709.1**	41.1	9.5	40.9	40.8	42.3	16.8	14.9		13.1	40.8	40.9
**Fxa6Bg103821.1**	60	15.6	64.8	63.4	51.7	23.7	26.5	13.1		52.6	53
**FvrTPS40**	75.7	20.5	80.8	80.5	83.2	33.3	31.8	40.8	52.6		97.2
**FcTPS40**	77	20.6	82.9	81.7	84.3	33.3	31.9	40.9	53	97.2	

**TPS Clade B, 2**	**FaTPS21**	**FaTPS10**	**FaTPS7**	**FaTPS6**	**FvTPS6**
**FaTPS21**		88.7	95.2	92	92.1
**FaTPS10**	88.7		92	92.6	92.5
**FaTPS7**	95.2	92		95.2	95
**FaTPS6**	92	92.6	95.2		97.9
**FvTPS6**	92.1	92.5	95	97.9	

**TPS Clade b, 3**	**FaTPS53**	**FaTPS52**	**FaTPS45**	**FaTPS44**	**FaTPS43**	**FaTPS10**	**Fxa6Cg103766.1**	**Fxa6Bg103826.1**	**FcTPS52**
**FaTPS53**		81.5	79.8	92.5	46.9	37.4	62.8	38.9	80.7
**FaTPS52**	81.5		84.3	80.2	46.5	37.5	50.7	42.3	98.8
**FaTPS45**	79.8	84.3		78.2	43.8	37	49	40.2	83.5
**FaTPS44**	92.5	80.2	78.2		46.8	36.3	59.2	37.4	80
**FaTPS43**	46.9	46.5	43.8	46.8		20.2	30.6	20.8	46.4
**FaTPS10**	37.4	37.5	37	36.3	20.2		24.6	18.2	37.4
**Fxa6Cg103766.1**	62.8	50.7	49	59.2	30.6	24.6		60.9	50.9
**Fxa6Bg103826.1**	38.9	42.3	40.2	37.4	20.8	18.2	60.9		42.5
**FcTPS52**	80.7	98.8	83.5	80	46.4	37.4	50.9	42.5	

**TPS Clade a1, Pinene**	**FaTPS 50**	**FaTPS 48**	**FaTPS 46**	**FaTPS 39**	**FaTPS 37**	**FaTPS 23**	**FaTPS 19**	**FaTPS 9**	**FaTPS 5**	**FaTPS 4**	**FaTPS 3**	**FaTPS 1**	**Fxa3Bg20 1803.1**	**Fxa2Ag1 00915.1**	**FvrTP S5**	**FvTPS5 0**	**FvTPS3 7**	**FvPIN S**
**FaTPS50**		42.7	60.4	60.5	60.4	55.1	29.5	27.5	57	60.1	54.5	95.4	32.5	46.6	53.1	94	61	93.4
**FaTPS48**	42.7		42.4	65.5	42.8	37.8	28.9	27.1	43.5	42.4	38.7	42.7	25.3	32.1	37.6	42.5	43.1	42.5
**FaTPS46**	60.4	42.4		59.1	94	53.9	29.3	27.8	86.5	94.9	53.8	61	32.8	67.4	81.4	59.8	91.6	59.6
**FaTPS39**	60.5	65.5	59.1		59.8	53.7	28.8	26.9	55.7	59.1	54.4	60.7	33.6	45.4	52.1	60.2	59.9	60.1
**FaTPS37**	60.4	42.8	94	59.8		53.8	29.1	27.6	87.4	93.3	53.9	60.9	32.9	67.1	82.2	59.7	95.2	59.6
**FaTPS23**	55.1	37.8	53.9	53.7	53.8		42.9	39.5	50.6	53.2	91.2	55.7	55.8	42.5	46.6	54.6	54.1	54.4
**FaTPS19**	29.5	28.9	29.3	28.8	29.1	42.9		87.1	29.6	28.9	42.1	30	27.3	22.7	25.5	29.1	29.3	29.2
**FaTPS9**	27.5	27.1	27.8	26.9	27.6	39.5	87.1		28.1	27.4	39	27.8	25.5	21.2	24	27.1	27.7	27.1
**FaTPS5**	57	43.5	86.5	55.7	87.4	50.6	29.6	28.1		89.8	50.9	58	31	65	80.3	57.1	87.8	56.6
**FaTPS4**	60.1	42.4	94.9	59.1	93.3	53.2	28.9	27.4	89.8		53.5	61.3	32.7	68	84.5	60.1	92.1	59.6
**FaTPS3**	54.5	38.7	53.8	54.4	53.9	91.2	42.1	39	50.9	53.5		55.2	54.4	41.9	47.5	54.4	55	54.3
**FaTPS1**	95.4	42.7	61	60.7	60.9	55.7	30	27.8	58	61.3	55.2		32.9	47.2	53.5	94.3	61.7	93
**Fxa3Bg201803.1**	32.5	25.3	32.8	33.6	32.9	55.8	27.3	25.5	31	32.7	54.4	32.9		21.2	37.9	32.2	33.2	32.2
**Fxa2Ag100915.1**	46.6	32.1	67.4	45.4	67.1	42.5	22.7	21.2	65	68	41.9	47.2	21.2		58.1	46.7	69.6	46.2
**FvrTPS5**	53.1	37.6	81.4	52.1	82.2	46.6	25.5	24	80.3	84.5	47.5	53.5	37.9	58.1		52.5	83.3	52.3
**FvTPS50**	94	42.5	59.8	60.2	59.7	54.6	29.1	27.1	57.1	60.1	54.4	94.3	32.2	46.7	52.5		60.8	98.7

**FvTPS37**	61	43.1	91.6	59.9	95.2	54.1	29.3	27.7	87.8	92.1	55	61.7	33.2	69.6	83.3	60.8		60.8
**FvPINS**	93.4	42.5	59.6	60.1	59.6	54.4	29.2	27.1	56.6	59.6	54.3	93	32.2	46.2	52.3	98.7	60.8	

**TPS Clade a1, 2**	**FaTPS63**	**FaTPS60**	**FaTPS36**	**FaTPS35**	**FaTPS34**	**FxTPS33**	**FaTPS31**	**FaTPS29**	**FaTPS27**	**FaTPS16**	**FvTPS35**
**FaTPS63**		59.4	42.7	43	42.1	38.1	42.3	41.7	42	60.6	42.7
**FaTPS60**	59.4		39.5	39.7	39.4	34.9	39.2	38.2	37.7	57.8	39.6
**FaTPS36**	42.7	39.5		98.4	93	56.5	91.8	87.4	83.6	51.5	97.7
**FaTPS35**	43	39.7	98.4		93.4	56.6	91.4	86.9	82.9	51.1	97.9
**FaTPS34**	42.1	39.4	93	93.4		55.8	91.8	86.6	82.1	50.3	92.3
**FaTPS33**	38.1	34.9	56.5	56.6	55.8		55.7	56.5	55	41.7	56.1
**FaTPS31**	42.3	39.2	91.8	91.4	91.8	55.7		90.9	86	49.7	91.6
**FaTPS29**	41.7	38.2	87.4	86.9	86.6	56.5	90.9		86.4	48.6	87.3
**FaTPS27**	42	37.7	83.6	82.9	82.1	55	86	86.4		47.4	82.6
**FaTPS16**	60.6	57.8	51.5	51.1	50.3	41.7	49.7	48.6	47.4		51.6
**FvTPS35**	42.7	39.6	97.7	97.9	92.3	56.1	91.6	87.3	82.6	51.6	

**TPS Clade a1, 3**	**FaTPS62**	**FaTPS59**	**FaTPS57**	**FaTPS54**	**FaTPS49**	**FaTPS47**	**FaTPS38**	**FaTPS26**	**FaTPS25**	**FaTPS24**	**FaTPS15**	**Fxa6Bg100956.1**	**Fxa4Bg102623.1**	**Fxa4Ag102687.1**
**FaTPS62**		87.2	97.2	88.8	57.8	57.1	51.1	57.2	55.5	29.9	51.4	42.4	36.7	31.8
**FaTPS59**	87.2		87.4	79	52.7	51.9	46.5	52.1	50.9	27	46.6	37.9	33.4	27.3
**FaTPS57**	97.2	87.4		89.5	58.6	57.9	51.7	58.1	56.3	30.4	51.7	42.8	37.3	32
**FaTPS54**	88.8	79	89.5		53.8	53.1	47.4	53.4	50.8	27.8	46.9	39.6	34.2	27.5
**FaTPS49**	57.8	52.7	58.6	53.8		97.3	85.1	95.7	76.4	49.1	52.9	68.3	61.1	43.9
**FaTPS47**	57.1	51.9	57.9	53.1	97.3		84.4	95.7	76.4	48.9	53.1	68.4	61	44.1
**FaTPS38**	51.1	46.5	51.7	47.4	85.1	84.4		83.6	67.2	49.5	47.5	59.9	61.5	38.3
**FaTPS26**	57.2	52.1	58.1	53.4	95.7	95.7	83.6		75.4	49.5	53.1	67.2	62.2	43.2
**FaTPS25**	55.5	50.9	56.3	50.8	76.4	76.4	67.2	75.4		39.5	49.1	56.9	48.5	45.9
**FaTPS24**	29.9	27	30.4	27.8	49.1	48.9	49.5	49.5	39.5		28.3	36	63.2	23.1
**FaTPS15**	51.4	46.6	51.7	46.9	52.9	53.1	47.5	53.1	49.1	28.3		45.1	34.1	36.6
**Fxa6Bg100956.1**	42.4	37.9	42.8	39.6	68.3	68.4	59.9	67.2	56.9	36	45.1		42.9	60.6
**Fxa4Bg102623.1**	36.7	33.4	37.3	34.2	61.1	61	61.5	62.2	48.5	63.2	34.1	42.9		27.5
**Fxa4Ag102687.1**	31.8	27.3	32	27.5	43.9	44.1	38.3	43.2	45.9	23.1	36.6	60.6	27.5	

**TPS Clade a1, 4**	**FaTPS64**	**FaTPS61**	**FaTPS58**	**FaTPS56**	**FaTPS55**	**FaTPS32**	**FaTPS30**	**FaTPS28**	**FaTPS20**	**FaTPS2**	**Fxa7Dg102852.1**	**Fxa7Cg103019.1**	**Fxa7Bg203107.2**	**Fxa3Ag102106.1**	**Fxa1Ag100363.1**	**FvrTPS55**	**FcTPS58**
**FaTPS64**		89.4	96.1	6.2	93.9	62.2	85.1	88.8	55.7	56.6	8.6	12.5	8.8	30.3	43.8	93.7	94.1
**FaTPS61**	89.4		89.4	6.3	88.1	59.2	85.4	83.9	52.4	52.7	8.7	12.5	8.9	28.6	41.2	88.3	89.3
**FaTPS58**	96.1	89.4		6.3	93.5	62.5	85.8	89.4	55.5	56.5	8.7	12.7	8.9	30.6	44	94.4	94.2
**FaTPS56**	6.2	6.3	6.3		6.1	5.2	5.9	6.2	5.7	5.6	69.4	46.7	68.2	2.9	4.5	6.3	6.3
**FaTPS55**	93.9	88.1	93.5	6.1		64.3	86.4	89.8	56.2	57	8.6	12.5	8.8	30.2	43.6	97.7	96.3

**FaTPS32**	62.2	59.2	62.5	5.2	64.3		60	60.9	57.8	59.8	7.3	10.7	7.5	30.5	45.6	63.7	63.6
**FaTPS30**	85.1	85.4	85.8	5.9	86.4	60		83.6	53	53.5	8.2	12.1	8.4	28.6	41.9	86.3	86
**FaTPS28**	88.8	83.9	89.4	6.2	89.8	60.9	83.6		53.9	55.2	8.7	12.8	9	29.3	42.2	89.6	89.3
**FaTPS20**	55.7	52.4	55.5	5.7	56.2	57.8	53	53.9		93.8	8	11.6	8.5	43.8	69	56.9	56.4
**FaTPS2**	56.6	52.7	56.5	5.6	57	59.8	53.5	55.2	93.8		7.9	11.3	8.2	45.2	72.8	57.5	57
**Fxa7Dg102852.1**	8.6	8.7	8.7	69.4	8.6	7.3	8.2	8.7	8	7.9		66.1	97.3	4.1	6.3	8.8	8.8
**Fxa7Cg103019.1**	12.5	12.5	12.7	46.7	12.5	10.7	12.1	12.8	11.6	11.3	66.1		66.2	5.9	9	12.8	12.8
**Fxa7Bg203107.2**	8.8	8.9	8.9	68.2	8.8	7.5	8.4	9	8.5	8.2	97.3	66.2		4.3	6.6	9	9
**Fxa3Ag102106.1**	30.3	28.6	30.6	2.9	30.2	30.5	28.6	29.3	43.8	45.2	4.1	5.9	4.3		42.1	30.8	30.3
**Fxa1Ag100363.1**	43.8	41.2	44	4.5	43.6	45.6	41.9	42.2	69	72.8	6.3	9	6.6	42.1		44	43.7
**FvrTPS55**	93.7	88.3	94.4	6.3	97.7	63.7	86.3	89.6	56.9	57.5	8.8	12.8	9	30.8	44		96.1
**FcTPS58**	94.1	89.3	94.2	6.3	96.3	63.6	86	89.3	56.4	57	8.8	12.8	9	30.3	43.7	96.1	

**Zhang et al., FaTPS1 (FaTPS26)**	**FaTPS49**	**FaTPS47**	**FaTPS38**	**FaTPS26**	**FaTPS24**	**Fxa6Bg100956.1**	**Fxa4Bg102623.1**	**Fxa4Ag102687.1**	***FaTPS1**
**FaTPS49**		97.3	85.2	95.7	49.3	68.1	61.4	43.9	96.3
**FaTPS47**	97.3		84.6	95.7	49.2	68.3	61.3	44.1	96.1
**FaTPS38**	85.2	84.6		83.8	49.7	59.9	62	38.5	84.4
**FaTPS26**	95.7	95.7	83.8		49.7	67	62.5	43.2	97.9
**FaTPS24**	49.3	49.2	49.7	49.7		36	64.6	23.2	50.2
**Fxa6Bg100956.1**	68.1	68.3	59.9	67	36		42.9	61.5	67
**Fxa4Bg102623.1**	61.4	61.3	62	62.5	64.6	42.9		27.6	63.5
**Fxa4Ag102687.1**	43.9	44.1	38.5	43.2	23.2	61.5	27.6		43.2
***FaTPS1**	96.3	96.1	84.4	97.9	50.2	67	63.5	43.2	

Supplementary Table S7. Volatile compound names, CAS numbers, SPME GC-MS retention times, and compound classifiers for metabolites analyzed in this study.

Compound	Retention Time	CAS	Compound Type
(-)-Myrtenol	21.7496	515-00-4	Terpene
1R-(-)-Myrtenal	18.6072	18486-69-6	Terpene
4,11-Selinadiene	19.5932	28290-20-2	Terpene
α-Farnesene	20.8517	502-61-4	Terpene
α-Guaiene	17.9	3691-12-1	Terpene
Allo-Ocimene	13.1479	7216-56-0	Terpene
α-Muurolene	20.4108	10208-80-7	Terpene
α-Pinene	4.442	80-56-8	Terpene
α-Terpineol	19.9054	10482-56-1	Terpene
β-Myrcene	7.5282	123-35-3	Terpene
β-Phellandrene	8.6986	555-10-2	Terpene
β-Pinene	6.1892	18172-67-3	Terpene
β-Selinene	20.3466	17066-67-0	Terpene
Caryophyllene	18.038	87-44-5	Terpene
Cis-1-p-Menthanol	14.1981	3901-95-9	Terpene
Cis-Linalool Oxide	14.751	5989-33-3	Terpene
7-epi-α-Selinene	21.2205	106-22-9	Terpene
δ-guaiene	20.3466	3691-11-0	Terpene
D-Limonene	8.3857	5989-27-5	Terpene
E- β -Farnesene	19.3607	18794-84-8	Terpene
Eremophilene	20.1864	10219-75-7	Terpene
Geraniol	22.6234	106-24-1	Terpene
Humulene	19.4489	6753-98-6	Terpene
Isopulegol	17.6773	7786-67-6	Terpene

Linalool	17.028	78-70-6	Terpene
Myrtenyl Acetate	19.6894	1079-01-2	Terpene
Nerolidol	25.9102	7212-44-4	Terpene
Selinane	20.5391	30824-81-8	Terpene
Terpinen-4-ol	18.1422	562-74-3	Terpene
Trans-Linalool Oxide	15.3843	34995-77-2	Terpene
Trans-Myrtanyl Acatate	20.9315	90934-53-5	Terpene
Trans-Pinocarvyl Acetate	19.04	1686-15-3	Terpene
1-Hexanol	12.7151	111-27-3	Alcohol
1-Octanol	17.2844	111-87-5	Alcohol
1-Octen-3-ol	14.9677	3391-86-4	Alcohol
1-Octen-3-yl Acetate	16.7392	2442-10-6	MCAAE
1-Pentanol	10.0695	71-41-0	Alcohol
2,4-Heptadienal E,E	15.8094	4313-03-5	Aldehyde
2,4-Hexadienal E,E	13.7011	142-83-6	Aldehyde
2,6-Nonadienal E,Z	17.8056	557-48-2	Aldehyde
2(3H)-Furanone, dihydro-5-methyl-5-(2-methylpropyl)-	28.4514	10200-21-2	Phenolic
2-Adamantanol-2-Bromomethyl	21.6294	1000142-34-3	Misc
2-Butenoic Acid Methyl Ester, E	6.1496	4358-59-2	SCBAE
2-Butyl Furan	6.7267	4466-24-4	Furan
2-Decenal, E	18.9038	3913-81-3	Aldehyde
2-Ethyl Furan	3.464	3208-16-0	Furan
2-Heptanol	11.8893	543-49-7	Alcohol
2-Heptanone	8.1056	110-43-0	Ketone
2-Heptenal,E	11.7771	18829-55-5	Aldehyde
2-Heptyl Butryrate	13.8133	39026-94-3	MCBAE
2-Hexen-1-ol,E	13.9977	928-95-0	Alcohol

2-Hexenal	9.0114	505-57-7	Aldehyde
2-Hexenal,E	8.5625	6728-26-3	Aldehyde
2-Hexenyl Butyrate,E	15.4326	53398-83-7	MCBAE
2-Methyl-but-2-En-1-yl Acetate	9.9413	33425-30-8	MCAAE
2-Methylbutyric Acid	19.4489	116-53-0	Acid
2-Nonanol	16.4588	628-99-9	Alcohol
2-Nonanol Acetate	15.144	14936-66-4	MCAAE
2-Octenal, E	14.3584	2548-87-0	Aldehyde
2-Penten-1-ol Acetate, Z	6.2137	42125-10-0	SCAAE
2-Pentenal, E	6.6626	1576-87-0	Aldehyde
2-Pentyl Furan	9.3802	3777-69-3	Furan
3-Ethyl-4-Methyl pentan-1-ol	8.851	38514-13-5	Alcohol
3-Formylamino-Succinimide	24.1865	99417-77-3	Misc
3-Hexen-1-ol	12.9555	928-96-1	Alcohol
3-Hexen-1-ol Acetate, E	11.5687	3681-82-1	MCAAE
3-Hexen-1-ol Acetate, Z	11.697	3681-71-8	MCAAE
3-Hexen-1-ol, E	13.44	928-97-2	Alcohol
3-Hexenal	6.8791	4440-65-7	Aldehyde
3-Hexenyl Butyrate	15.144	16491-36-4	MCBAE
3-Pentanol	6.3417	584-02-1	Alcohol
4,4-Dimethylpent-2-enal	8.0334	22597-46-2	Aldehyde
4-Hexenoic Acid	24.2824	1577-20-4	Acid
4-Methyl-5-nonanone	21.461	35900-26-6	Ketone
4-Methyl-Pentanoic Acid	21.926	646-07-1	Acid
5-Methyl-Hexanoic Acid	23.6494	628-46-6	Acid
Benzaldehyde	16.2984	100-52-7	Phenolic
Benzyl Acetate	20.5391	140-11-4	Phenolic
Benzyl Butyrate	23.0163	103-37-7	Phenolic
Butanethioic Acid-S-Methyl Ester	8.53	2432-51-1	Sulfur

Butanoic Acid	18.6072	107-92-6	Acid
Butyl Acetate	5.6203	123-86-4	SCAAE
Butyl Butyrate	9.0676	109-21-7	SCBAE
Butyl Hexanoate	13.9977	626-82-4	SCHAE
Cinnamyl Acetate	27.5451	103-54-8	Phenolic
Cis-3-Hexenyl Hexanoate	19.2405	31501-11-8	MCHAE
Decanal	15.9938	112-31-2	Aldehyde
Decyl Acetate	19.6894	112-17-4	MCAAE
Decyl Butyrate	22.2866	5454-09-1	MCBAE
Decyl Isobutyrate	22.1184	5454-22-8	MCAAE
Ethyl-(Z)-Cinnamate	27.2565	4610-69-9	Phenolic
Ethyl-2-Methyl Butyrate	5.0673	7452-79-1	SCBAE
Ethyl Benzoate	19.3607	93-89-0	Phenolic
Ethyl Butyrate	4.74	105-54-4	SCBAE
Ethyl Caprate	18.8156	110-38-3	SCDAE
Ethylene Glycol Dibutyrate	20.9315	105-72-6	Misc
Ethyl Hexanoate	9.5085	123-66-0	SCHAE
Ethyl Isobutyrate	20.1864	97-62-1	SCBAE
Ethyl Isocaproate	8.4258	25415-67-2	SCVAE
Ethyl Isovalerate	5.4	108-64-5	SCBAE
Ethyl Octanoate	14.5508	106-32-1	SCOAE
Ethyl Pentanoate	6.8791	539-82-2	SCVAE
Ethyl Propinoate	3.5122	105-37-3	SCPAE
Eugenol	27.7539	97-53-0	Phenolic
Furaneol	25.7574	3658-77-3	Furan
Heptanal	8.1377	111-71-7	Aldehyde
Heptanoic Acid	24.4511	111-14-8	Acid
Heptyl Acetate	13.1479	112-06-1	MCAAE
Hexanal	5.5964	66-25-1	Aldehyde

Hexane-3-Ethyl-4-Methyl	10.1097	3074-77-9	Misc
Hexanoic Acid	22.7597	142-62-1	Acid
Hexyl-2-Methylbutyrate	14.4624	10032-15-2	MCBAE
Hexyl Acetate	10.6628	142-92-7	MCAAE
Hexyl Butyrate	14.1981	626-82-4	MCBAE
Hexyl Hexanoate	18.3506	6378-65-0	MCHAE
Hexyl Isovalerate	14.8635	10032-13-0	MCBAE
Hexyl Propanoate	12.2742	2445-76-3	MCPAE
Hydrocinnamyl Isobutyrate	24.2824	103-58-2	Phenolic
Isoamyl Acetate	6.5018	123-92-2	SCAAE
Isoamyl Alcohol	8.8991	123-51-3	Alcohol
Isoamyl Butyrate	10.3662	106-27-4	SCBAE
Isobutyl Butyrate	7.4643	539-90-2	SCBAE
Isobutyl Hexanoate	12.6429	105-79-3	SCHAE
Isobutyric Acid	17.4127	79-31-2	Acid
Isopentyl Hexanoate	15.088	2198-61-0	SCHAE
Isopentyl Isovalerate	11.1679	659-70-1	SCBAE
Isopropyl Butyrate	4.8429	638-11-9	SCBAE
Isovaleric Acid	19.5932	503-74-2	Acid
Mesifurane	17.9418	4077-47-8	Furan
Methyl-2-Hydroxybutyrate	13.2681	29674-47-3	SCBAE
Methyl-2-Methyl Butyrate	4.2895	868-57-5	SCBAE
Methyl-3-Methylthio-Propionate	16.4588	13532-18-8	Sulfur
Methyl Anthranilate	28.5476	134-20-3	Phenolic
Methyl Butyrate	3.9	623-42-7	SCBAE
Methyl Cinnamate	26.3912	103-26-4	Phenolic
Methyl Decanoate	17.9418	110-42-9	SCDAE
Methyl Hexanoate	8.1773	106-70-7	SCHAE
Methyl Isobutyl Ketone	4.2095	108-10-1	Ketone

Methyl Isohexanoate	7.0074	2177-83-5	SCVAE
Methyl Isovalerate	4.426	556-24-1	SCBAE
Methyl Nonanoate	15.8896	1731-84-6	SCNAE
Methyl Octanoate	13.4204	111-11-5	SCOAE
Methyl Salicylate	21.3568	119-36-8	Phenolic
Methyl Thiolacetate	4.9631	1534-08-3	Sulfur
Methyl Valerate	5.6846	624-24-8	SCVAE
Nonanal	13.4766	124-19-6	Aldehyde
Nonanoic Acid	27.7539	112-05-0	Acid
Octan-8-o-l2,5-Diaza-2,5-Dimethyl	10.8312	26439-05-4	Misc
Octanal	10.9754	124-13-0	Aldehyde
Octanoic Acid	26.1827	124-07-2	Acid
Octyl-2-Methyl Butyrate	18.7034	29811-50-5	MCBAE
Octyl Acetate	15.4326	112-14-1	MCAAE
Octyl Butyrate	18.4228	110-39-4	MCBAE
Octyl Hexanoate	22.0383	4887-30-3	MCHAE
Octyl Isovalerate	19.04	7786-58-5	MCBAE
Octyl Propionate	16.8676	142-60-9	MCPAE
Pent-1-en-3-ol	7.6005	616-25-1	Alcohol
Pentanoic acid, 2,2,4-trimethyl-3-carboxyisopropyl, isobutyl ester	23.2086	1000140-77-5	Misc
Pentanoic acid, 2-Methyl Anhydride	11.9534	63169-61-9	MCHAE
Pentanoic Acid	20.7152	109-52-4	Acid
Pentyl Acetate	7.849	628-63-7	SCAAE
Phenethyl Acetate	22.1184	103-45-7	Phenolic
S-Methyl Isovalerate	9.2038	23747-45-7	Sulfur
Styrene	10.0376	100-42-5	Misc
Trans-2-Hexenyl Acetate,E	12.2261	2497-18-9	MCAAE
Trans-2-Hexenyl Isovalerate	15.9938	68698-59-9	MCBAE

γ-Decalactone	27.4413	706-14-9	Lactone
γ-Dodecalactone	29.8943	2305-05-7	Lactone
γ-Octalactone	23.8099	104-50-7	Lactone
γ-Pentalactone	21.926	108-29-2	Lactone
γ-Undecalactone Standard	28.8201	104-67-6	Standard
Z-Pent-2-enyl Butyrate	13.0117	42125-13-3	SCVAE

Supplementary Table S8: Reagents and authentic standards used in this study. Equations listed for standard calibration curves of eight terpenes run and reported as linear regressions.

Standard	CAS #	Curve R2	Range	Equation
Linalool	78-70-6	.991	2 ng/mL - 1110 ng/mL	4345.18x
(1R)-(-)-Myrtenal	18486-69-6	.988	0.1 ng/mL - 5 ng/mL	59902.65x
(-)-Myrtenol	19894-97-4	.957	1 ng/mL - 400 ng/mL	2532.21x
(-)-Myrtenyl Acetate	36203-31-3	.98	0.2 ng/mL - 150 ng/mL	7014.49x
Nerolidol	7212-44-4	.971	0.2 ng/mL - 120 ng/mL	1423.46x
α-Pinene	80-56-8	.943	0.04 ng/mL - 2 ng/mL	1622687x
(-)-β-Pinene	18172-67-3	.967	0.04 ng/mL - 2 ng/mL	1438854x
Terpineol	8000-41-7	.99	1 ng/mL - 200 ng/mL	397.25x
γ-Undecalactone Internal Standard	104-67-6	.987	0.2 ng/mL - 120 ng/mL	753.85x
Citronellol	106-22-9	NA	NA	NA
Farnesene	502-61-4	NA	NA	NA
Farnesyl Pyrophosphate	13059-04-3	NA	NA	NA
Geranyl Pyrophosphate	763-10-0	NA	NA	NA
Borneol	464-45-9	NA	NA	NA
Caryophyllene	87-44-5	NA	NA	NA
Germacrene D	23986-74-5	NA	NA	NA
(R)-(+)- Limonene	5989-27-5	NA	NA	NA
(S)-(-)-Limonene	5989-54-8	NA	NA	NA
Isobutyl Benzene Internal Standard	538-93-2	NA	NA	NA
Linalool oxide	60047-17-8	NA	NA	NA
β-Elemene	515-13-9	NA	NA	NA
Humulene	6753-98-6	NA	NA	NA

Supplementary Table S9: Kovats Indices calculated for selected terpene products.			
**Gene ID**	**Calculated KI**	**Predicted compound**	**Compound Number**
FaTPS10	1046	(*E*)-β-Ocimene	5
FcTPS52	1035	(*Z*)-β-Ocimene	6
FvTPS50	1075	*Trans*-thujanol	4
FvTPS37	1051	(*E*)-β-Ocimene	5
FvTPS37	1106	*trans*-thujanol	4
FvTPS6	1670	Bulnesol	14
FaTPS10	1763	β-acoradienol	15
FcTPS52	1743	Khusimol	18
FaTPS10	1882	8,13Cedranediol	16
FcTPS52	1861	α-Chenopodiol	19
FcTPS52	1868	8,13-Cedranediol	16
FaTPS36	1558	*trans*-Dauca-4(11),7-diene	ii
FaTPS35	1397	β-Elemene	21
FaTPS36	1505	β-Bisabolene	23
FaTPS36	1397	Cyperene	34
FaTPS36	1471	β-Acoradiene	24
FaTPS36	1715	Longifolol	35
FaTPS36	1837	Eudesm-7(11)-en-4-ol acetate	36
FaTPS35	1769	Hinesol acetate	37
FcTPS58	1495	Viridiflorene	29
FcTPS58	1508	Germacrene A	30
FcTPS58	1527	Macrocarpene	31
FcTPS58	1533	γ-Cuprenene	38
FaTPS55	1557	*cis*-Cadinene ether	39
FaTPS55	1591	Globulol	33
FaTPS55	1626	Eremoligenol	32
FaTPS55	1635	α-Acorenol	40
FvrTPS55	1495	γ-Amorphene	41
FaTPS55	1591	Globulol	33

Supplementary Table S10. Harvest and accession information for this study.

	Alias	RNA	UC_ID	2021 Reps	2021 Harvests	2022 Reps	2022 Harvests	Species	Subspecies	Breeding Date	Origin
**1**	WLSP-08		PI551453	5	2	6	2	*F. chiloensis*	lucinda	NA	WA
**2**	LCM-10		PI551468	4	2	0	0	*F. chiloensis*	lucinda	NA	OR, Pacific coast
**3**	Ambato	**	PI551736	4	2	7	3	*F. chiloensis*	chiloensis	NA	Ecuador
**4**	Isle of Lemuy (02A White)	**	PI552038	7	3	0	0	*F. chiloensis*	NA	NA	Chile
**5**	CFRA 688		PI612487	8	3	0	0	*F. chiloensis*	pacifica	NA	BC, Pacific coast + Alaska
**6**	CFRA 1267		PI612488	6	2	4	2	*F. chiloensis*	pacifica	NA	BC, Pacific coast + Alaska
**7**	UC04	**	PI551498	7	4	0	0	*F. vesca*	NA	NA	CA bred; Asia-Temperate, Europe
**8**	UC06	**	PI551514	9	5	7	3	*F. vesca*	NA	NA	CA West coast, south/central states/Mexico
**9**	Harris Springs	**	17X004P001	4	2	0	0	*F. virginiana*	platypetala	NA	CA
**10**	LH_18-2		PI551876	4	2	0	0	*F. virginiana*	glauca	NA	Wyoming. Pacific Coast and Midwest
**11**	Hinesburg		PI552277	6	2	3	3	*F. virginiana*	virginiana	NA	Vermont
**12**	JP_95-9-6		PI612320	6	3	6	2	*F. virginiana*	grayana	NA	Georgia, East and central States
**13**	NC_96-35-2	**	PI612323	7	3	3	1	*F. virginiana*	virginiana	NA	Alabama North and East North America
**14**	Fredrk._9		PI612493	6	4	4	2	*F. virginiana*	NA	NA	Ontario, Canada
**15**	RH_30		PI612499	3	2	3	1	*F. virginiana*	virginiana	NA	Minnesota, North and East North America
**16**	KY-17		PI616574	3	2	3	1	*F. virginiana*	grayana	NA	Kentucky, East and central States

**17**	NC_95-11-1		PI616691	8	4	5	2	*F. virginiana*	virginiana	NA	South Carolina North and East North America
**18**	NC_95-21-5		PI616720	5	3	3	1	*F. virginiana*	grayana	NA	Mississippi, East and central States
**19**	NC_96-20-3		PI616789	8	3	0	0	*F. virginiana*	virginiana	NA	Alabama North and East North America
**20**	NC_96-33-1		PI616815	3	3	0	0	*F. virginiana*	virginiana	NA	Alabama North and East North America
**21**	NC_96-14-1		PI616902	9	3	11	4	*F. virginiana*	virginiana	NA	North Carolina North and East North America
**22**	UC11		PI551495	8	3	0	0	*F. virginiana*	NA	NA	CA North and East North America
**23**	UC12		PI551497	3	2	0	0	*F. virginiana*	NA	NA	CA North and East North America
**24**	Jucunda		PI551623	5	3	6	4	*F. x ananassa*	NA	1854	England
**25**	Weisse Anasa		PI270464	5	2	4	4	*F. x ananassa*	NA	1867	Germany
**26**	Sitka		PI616777	14	6	4	2	*F. x ananassa*	NA	1905	Alaska
**27**	Howard 17		PI551593	5	2	5	2	*F. x ananassa*	NA	1907	Massachusetts
**28**	Ettersburg 121		PI551904	8	3	4	2	*F. x ananassa*	NA	1907	CA
**29**	Madame Moutot	**	PI551632	7	3	5	2	*F. x ananassa*	NA	1910	France
**30**	Kaiser Samling		PI270471	7	4	4	2	*F. x ananassa*	NA	1912	Germany
**31**	Aberdeen		PI551630	6	3	5	3	*F. x ananassa*	NA	1917	New Jersey
**32**	Blakemore		PI551421	13	5	9	3	*F. x ananassa*	NA	1929	Maryland
**33**	Sparkle		PI551559	8	3	3	1	*F. x ananassa*	NA	1942	New Jersey
**34**	Shasta		35C035P008	8	4	6	2	*F. x ananassa*	NA	1945	UC Berkeley (Davis)

**35**	Morioka 17		PI551428	9	4	9	3	*F. x ananassa*	NA	1945	Japan
**36**	Freja		PI551628	5	2	3	1	*F. x ananassa*	NA	1948	Denmark
**37**	Albritton		PI551435	9	3	10	4	*F. x ananassa*	NA	1951	North Carolina
**38**	Empire		PI551569	15	6	9	3	*F. x ananassa*	NA	1951	New York
**39**	Wiltguard		52C016P007	8	4	6	2	*F. x ananassa*	NA	1952	UC Davis
**40**	Direktor Paul Wallbaum	**	PI551436	12	4	4	2	*F. x ananassa*	NA	1953	Germany
**41**	Senga Sengana		PI264680	6	2	6	2	*F. x ananassa*	NA	1954	Germany
**42**	EarliMiss	**	PI551862	12	5	15	5	*F. x ananassa*	NA	1955	Mississippi
**43**	K1_1953		PI616778	8	3	12	4	*F. x ananassa*	NA	1955	Alaska
**44**	Red Gauntlet		PI551530	6	2	9	3	*F. x ananassa*	NA	1957	Scotland
**45**	Headliner	**	PI551652	9	3	9	3	*F. x ananassa*	NA	1957	Louisiana
**46**	Tioga		53C009P002	8	4	10	4	*F. x ananassa*	NA	1964	UC Davis
**47**	Hood		PI551502	4	2	7	3	*F. x ananassa*	NA	1965	Oregon
**48**	Linn	**	PI551500	9	3	9	4	*F. x ananassa*	NA	1967	Oregon
**49**	Guardian		PI551407	6	2	6	3	*F. x ananassa*	NA	1969	Maryland
**50**	Primella	**	PI551422	9	4	6	2	*F. x ananassa*	NA	1969	Netherlands
**51**	Douglas		70C003P108	6	2	11	5	*F. x ananassa*	NA	1970	UC Davis
**52**	Titan		PI551398	5	2	5	2	*F. x ananassa*	NA	1971	North Carolina
**53**	Totem		PI551501	8	3	6	3	*F. x ananassa*	NA	1971	British Colombia, Canada
**54**	Tufts		63C120P011	5	2	11	4	*F. x ananassa*	NA	1972	UC Davis
**55**	EarliGlow		PI551394	8	3	6	2	*F. x ananassa*	NA	1975	Maryland

**56**	Florida Belle		PI551396	9	4	6	2	*F. x ananassa*	NA	1975	Florida
**57**	Tangi	**	PI551481	6	3	9	3	*F. x ananassa*	NA	1975	Louisiana
**58**	Kaoling		PI551537	7	3	8	4	*F. x ananassa*	NA	1975	Taiwan
**59**	ORUS_4816_ORUSM_173		PI551858	7	3	6	2	*F. x ananassa*	NA	1975	Oregon
**60**	Chandler		77C032P103	9	3	8	4	*F. x ananassa*	NA	1977	UC Davis
**61**	Hecker		69C141P101	6	3	12	4	*F. x ananassa*	NA	1979	UC Davis
**62**	Brighton		PI551494	6	3	5	2	*F. x ananassa*	NA	1979	UC Davis
**63**	Elsanta		PI551579	9	3	6	3	*F. x ananassa*	NA	1981	Netherlands
**64**	MDUS 5130	**	PI551946	6	2	6	2	*F. x ananassa*	NA	1981	Maryland
**65**	Selva		75C071P107	5	2	11	4	*F. x ananassa*	NA	1982	UC Davis
**66**	Glooscap		PI551580	10	4	8	3	*F. x ananassa*	NA	1983	Nova Scotia
**67**	Tillikum		PI551832	7	3	10	4	*F. x ananassa*	NA	1983	Washington
**68**	Seascape		83C049P001	6	3	6	3	*F. x ananassa*	NA	1984	UC Davis
**69**	Camarosa		88C029P603	7	3	7	3	*F. x ananassa*	NA	1988	UC Davis
**70**	Beaver Belle	**	PI551839	6	2	8	4	*F. x ananassa*	NA	1989	Canada
**71**	Pelican		PI637960	8	4	8	4	*F. x ananassa*	NA	1989	Maryland
**72**	Mara des Bois	**	17Z001P001	6	3	9	3	*F. x ananassa*	NA	1991	France
**73**	Diamante		91C248P006	7	3	9	4	*F. x ananassa*	NA	1991	UC Davis
**74**	Camino Real		94C003P011	7	3	5	2	*F. x ananassa*	NA	1994	UC Davis
**75**	Puget Reliance		PI664321	6	2	3	1	*F. x ananassa*	NA	1995	Washington
**76**	Ventana		96C042P601	9	3	9	5	*F. x ananassa*	NA	1996	UC Davis
**77**	Albion		97C117P003	8	4	5	3	*F. x ananassa*	NA	1997	UC Davis

**78**	Monterey		01C132P003	7	3	3	1	*F. x ananassa*	NA	2001	UC Davis
**79**	San Andreas		01C139P002	9	3	0	0	*F. x ananassa*	NA	2001	UC Davis
**80**	Portola		01C206P005	7	3	9	4	*F. x ananassa*	NA	2001	UC Davis
**81**	Petaluma		08C020P602	8	4	6	3	*F. x ananassa*	NA	2008	UC Davis
**82**	Grenada		08C055P002	6	3	4	2	*F. x ananassa*	NA	2008	UC Davis
**83**	Fronteras		08C132P608	7	3	4	2	*F. x ananassa*	NA	2008	UC Davis
**84**	UCD_Warrior		08C138P003	7	3	6	3	*F. x ananassa*	NA	2008	UC Davis
**85**	Cabrillo		08C181P001	5	2	8	4	*F. x ananassa*	NA	2008	UC Davis
**86**	11-58_FVC		FVC_11-58	9	6	5	4	*F. x ananassa*	NA	2010	Michigan
**87**	UCD_Victor		11C057P001	5	2	3	1	*F. x ananassa*	NA	2011	UC Davis
**88**	UCD_Valiant		11C103P001	7	3	5	2	*F. x ananassa*	NA	2011	UC Davis
**89**	UCD_Moxie		11C141P001	6	2	8	3	*F. x ananassa*	NA	2011	UC Davis
**90**	UCD_Royal_Royce (Royal Royce)	**	08C123P001	6	2	3	1	*F. x ananassa*	NA	2008	UC Davis
**91**	UCD_Finn		12C112P004	7	3	3	1	*F. x ananassa*	NA	2012	UC Davis
**92**	UCD_Mojo		12C166P002	5	2	9	3	*F. x ananassa*	NA	2012	UC Davis
**93**	16C111P068		16C111P068	11	5	13	5	*F. x ananassa*	NA	2016	UC Davis
**94**	17EDN012		12C104P004	6	3	8	4	*F. x ananassa*	NA	2017	UC Davis
**95**	17EDN013		12C104P005	8	3	3	2	*F. x ananassa*	NA	2017	UC Davis
**96**	17C224P011	**	17C224P011	14	6	10	4	*F. x ananassa*	NA	2017	UC Davis
